# Global, regional, and national burden of chronic kidney disease among adolescents and emerging adults from 1990 to 2021

**DOI:** 10.1080/0886022X.2025.2508296

**Published:** 2025-05-22

**Authors:** Zhi Wang, Qingqing You, Yuxuan Wang, Jufei Wang, Leping Shao

**Affiliations:** a The Graduate School of Fujian Medical University, Fujian Medical University, Fuzhou, China; b Department of Nephrology, Qingdao Municipal Hospital (Group), Qingdao Hospital of University of Health and Rehabilitation Sciences, Qingdao, China; c Department of Emergency, Qingdao Municipal Hospital (Group), Qingdao Hospital of University of Health and Rehabilitation Sciences, Qingdao, China

**Keywords:** Adolescents, emerging adults, CKD, GBD, incidence

## Abstract

**Background and Aims:**

There are limited studies on the epidemiology of chronic kidney disease (CKD) burden among adolescents and emerging adults. We aimed to assess the global, regional, and national trends in CKD burden among adolescents and emerging adults.

**Methods:**

The Global Burden of Disease 2021 study was utilized to evaluate the incidence, prevalence, mortality, disability-adjusted life years (DALYs) and average annual percentage changes (AAPC) in CKD among populations aged 15 to 29 years from 1990 to 2021.

**Results:**

From 1990 to 2021, age-standardized incidence (AAPC: 0.85%, 95% uncertainty interval [95% UI]: 0.81%-0.88%), prevalence (AAPC: 0.22%, 95% UI: 0.19%–0.25%), and mortality (AAPC: 0.18%, 95% UI: 0.04%–0.32%) rates of CKD have risen globally among adolescents and emerging adults. In 2021, Southeast Asia had the highest age-standardized prevalence (5370.39 [95% UI: 4060.97–6929.79] per 100,000 population), while Central sub-Saharan Africa had the highest mortality rate (5.05 [95% UI: 3.49–7] per 100,000 population). In 2021, glomerulonephritis and ‘other and unspecified causes’ accounted for 94% of new cases, 83% of prevalent cases, and 92% of mortality cases. Frontier analyses suggest that regions at varying stages of development still hold substantial potential for further improvements in addressing CKD.

**Conclusion:**

Globally, the burden of CKD among adolescents and emerging adults continues to rise, with Southeast Asia and sub-Saharan Africa bearing a disproportionate burden. Nevertheless, there remain substantial opportunities across all levels of the development spectrum to alleviate the CKD burden through enhanced health interventions and resource allocation.

## KEY LEARNING POINTS

**What was known:**

CKD presents a significant public health problem that affects people of all ages. In the age group of 15–29 years, referred to as adolescents and emerging adults, the epidemiological study of CKD in this demographic has been limited.

**This study adds:**

This was the first study to comprehensively analyze the trends in the burden of CKD among adolescents and emerging adults at global, regional, and national levels.

**Potential impact:**

The burden of CKD among adolescents and emerging adults requires attention. The results of this study provide a basis for formulating subsequent disease prevention and control policies.

## Introduction

Chronic kidney disease (CKD) is characterized as sustained damage to the kidney parenchyma that leads to impaired kidney functions and ultimately progresses to end-stage kidney disease [1]. In 2019, an estimated 697 million cases of CKD globally marked it as a serious public health problem [2].

Those aged 15–29 years, often referred to as adolescents and emerging adults, represent critical periods of development, including physical and psychological maturation and transitions in social roles [3–4]. This age group faces challenges in health literacy [5], particularly in the self-management of CKD, where there are gaps in both understanding and practical barriers [6]. Despite this group accounting for only a small share of end-stage kidney disease cases, their mortality rate significantly exceeds that of their healthy peers, resulting in a significant loss of life [7–8].

Limited data exists on the epidemiology of CKD among adolescents and emerging adults, with significant global variation attributed to disparities in diagnostic capabilities. Therefore, it is imperative to investigate the evolving epidemiology of CKD in this vulnerable population. In this study, we summarized and analyzed the age-standardized incidence (ASIR), prevalence (ASPR), mortality (ASMR), and disability-adjusted life years (DALYs)(ASDR) related to CKD in individuals aged 15 to 29 years at global, regional, and national level during 1990–2021, considering sex, age, cause and social development levels. Our research findings will provide a critical foundation for government agencies in formulating effective prevention and control strategies for chronic kidney disease in this population.

## Materials and method

### Date source

The CKD data analyzed in this study are derived from the GBD 2021, which provides the latest estimates of epidemiological data on the burden of 371 diseases and injuries across 21 GBD regions and 204 countries and territories from 1990 to 2021 [9]. In GBD 2021, CKD is defined as an estimated glomerular filtration rate of less than 60 mL/min/1.73 m^2^ or an albumin-to-creatinine ratio greater than 30 mg/g. We extracted the data on the number of cases and the incidence, prevalence, mortality, and DALYs of CKD among populations aged 15–29 years from 1990 to 2021. This comprehensive dataset encompassed variables including age, sex, cause, sociodemographic index (SDI), region, and country. DALYs represent the years of healthy life lost, calculated using the formula: DALYs = years of life lost (YLLs) + years lived with disability (YLDs). YLLs is the product of the number of deaths and the difference between the age at death and the standard life expectancy. YLDs refers to the years of life lost due to any short- or long-term health loss, weighted by disability severity using disability weights. These are estimated by multiplying the prevalence by corresponding disability weights. Additionally, we extracted the DALYs attributed to each risk factor to quantify the contribution of specific risk factors to the disease burden.

### Statistical analysis

Based on data obtained from the CKD 2021, we calculated age-standardized rates and corresponding 95% uncertainty intervals (95% UI) using the world standard population reported in the GBD 2017 [10]. This allowed for the comparison of CKD burdens across different age groups, sexes, causes, regions, and countries. Specifically, this includes ASIR, ASPR, ASMR, and ASDR (per 100,000 population, respectively). The formula for age-standardized rate is as follows:

$$\text{Age}-\text{standardized rate}=\frac{\sum_{i=1}^{A}a_{i}w_{i}}{\sum_{i=1}^{A}w_{i}}$$

For the formula: where a_i_ is the age-specific rate, w_i_ is the weight in the same age subgroup of the chosen reference standard population (in which i denotes the i^th^ age class), and A is the upper age limit.

The temporal trends in the burden of CKD among global adolescents and emerging adults were analyzed using Joinpoint regression [11]. This model divides the overall trend into multiple segments based on joinpoints and calculates the annual percentage change (APC) along with its 95% UI for each segment. The average annual percentage change (AAPC) summarizes the trend over the entire period, representing the weighted average of the APCs across all segments. In this study, AAPC is used to describe the average annual rate of change from 1990 to 2021. The formula for calculating AAPC is as follows:

$$\text{AAPC}=\{\text{exp}[\frac{\sum w_{i}b_{i}}{\sum w_{i}}]-1\}$$

For the formula: b_i_ is the slope coefficient for the i^th^ segment with i indexing the segments in the desired range of years and w_i_ is the length of each segment in the range of years.

The Slope Index of Inequality (SII) and the Relative Concentration Index (RCI) were employed to assess cross-national inequalities in the burden of adolescents and emerging adults CKD^11^. The SII serves as an absolute measure of inequality, representing the absolute difference in DALYs between the countries with the highest and lowest levels of the SDI. This index utilizes an appropriate regression model to account for the entire distribution of SDI. An SII value of zero indicates no inequality. A larger absolute value of the SII reflects a higher degree of inequality. A positive SII indicates that DALYs are concentrated in countries with higher SDI levels, while a negative SII suggests that DALYs are concentrated in countries with lower SDI levels. The RCI is a relative measure of inequality, quantifying the health gradient across countries with different SDI levels. It indicates the concentration of DALYs in countries with either low or high SDI levels. The RCI ranges from −1 to +1, with a value of zero indicating no inequality. A positive RCI suggests that DALYs is concentrated in countries with higher SDI, while a negative RCI indicates that DALYs is concentrated in countries with lower SDI. The greater the absolute value of the RCI, the higher the degree of inequality.

To assess the relationship between the burden of CKD and demographic development, we applied a frontier model of ASDR constructed using the SDI framework [12]. The focus of the frontier analysis is to determine the theoretically achievable minimum ASDR (i.e., frontier) for each country, based on its current level of development. Data Envelopment Analysis (DEA) was employed for this purpose. To ensure the robustness of our findings, we conducted 1,000 bootstrap samples and computed the average ASDR for each SDI value. LOESS regression with a local polynomial degree of 1 and span of 0.5 was applied to generate a smoothed frontier. The improvement potential for each country was quantified by measuring the absolute distance (i.e., effective gap) between the 2021 ASDR and the frontier.

All statistical analyses were conducted using R (version 4.3.3), GraphPad Prism (version 10.0), and the Joinpoint Regression Program (version 5.0).

## Results

### Global trends

In 2021, the ASIR, ASPR, ASMR, and ASDR for CKD among adolescents and emerging adults globally were 23.13 (95% UI: 12.92–35.6), 4041.58 (95% UI: 3086.92–5179.46), 2.04 (95% UI: 1.82–2.27), and 165.14 (95% UI: 144.82–187.02) per 100,000 population, respectively (Table 1, Supplementary Table S1). From 1990 to 2021, ASIR, ASPR, and ASMR increased at varying rates. The ASIR rose by 30% (AAPC: 0.85%, 95% UI: 0.81%–0.88%), the ASPR increased by 7% (AAPC: 0.22%, 95% UI: 0.19%–0.25%), and the ASMR increased by 6% (AAPC: 0.18%, 95% UI: 0.04%–0.32%). Although the ASDR showed no significant change over the entire period (AAPC: 0.1%, 95% UI: −0.04%–0.24%), a notable upward trend has emerged since 2013 (APC: 0.44%, 95% UI: 0.33%–0.54%) (Supplementary Table S2–3, Supplementary Figure S1). Similarly, the ASMR showed a more pronounced increase after 2013 (APC: 0.61%, 95% UI: 0.47%–0.76%).

**Table 1.** Global incidence of CKD and their AAPCs from 1990 to 2021 by sex, age, cause, SDI, and region in adolescents and emerging adults.

Measure	Variable	1990		2021		AAPC (95% UI)
		Number (95% UI)	ASR (95% UI)	Number (95% UI)	ASR (95% UI)	
Incidence	Global	258,180.10 (123,472.90 to 425,874.01)	17.84 (8.53 to 29.44)	418,865.95 (233,830.97 to 644,908.73)	23.13 (12.92 to 35.60)	0.85 (0.81 to 0.88)
	**Sex**					
Incidence	Male	121,819.94 (59,107.22 to 200,767.31)	16.66 (8.08 to 27.49)	216,495.27 (123,589.32 to 324,680.86)	23.51 (13.43 to 35.24)	1.12 (1.10 to 1.14)
Incidence	Female	136,360.16 (63,597.03 to 224,283.78)	19.03 (8.87 to 31.32)	202,370.68 (107,283.17 to 318,975.70)	22.73 (12.06 to 35.81)	0.58 (0.56 to 0.60)
	**Age**					
Incidence	15–19	79,997.95 (39,867.68 to 131,184.84)	15.40 (7.68 to 25.26)	138,648.14 (82,838.22 to 205,213.23)	22.22 (13.28 to 32.89)	1.19 (1.15 to 1.23)
Incidence	20–24	83,727.96 (39,380.33 to 135,022.26)	17.01 (8.00 to 27.44)	131,944.03 (74,021.78 to 197,404.46)	22.10 (12.40 to 33.06)	0.85 (0.79 to 0.91)
Incidence	25–29	94,454.19 (44,224.88 to 159,666.91)	21.34 (9.99 to 36.07)	148,273.78 (76,970.96 to 242,291.03)	25.20 (13.08 to 41.18)	0.54 (0.50 to 0.58)
	**Cause**					
Incidence	Diabetes Mellitus Type 1	3,426.91 (1,659.42 to 5,747.99)	0.23 (0.11 to 0.39)	8,904.04 (4,892.25 to 13,813.87)	0.49 (0.27 to 0.77)	2.44 (2.35 to 2.54)
Incidence	Diabetes Mellitus Type 2	5,219.76 (2,455.76 to 8,765.43)	0.37 (0.17 to 0.61)	5,105.33 (2,778.49 to 8,060.94)	0.28 (0.15 to 0.44)	−0.85 (−0.88 to −0.82)
Incidence	Hypertension	7,184.78 (3,411.56 to 12,025.44)	0.50 (0.24 to 0.83)	11,719.14 (6,415.70 to 18,246.04)	0.65 (0.35 to 1.01)	0.85 (0.80 to 0.89)
Incidence	Glomerulonephritis	28,540.65 (14,022.10 to 46,947.04)	1.96 (0.96 to 3.22)	48,647.84 (27,975.40 to 73,126.19)	2.70 (1.55 to 4.05)	1.03 (0.96 to 1.10)
Incidence	Other and Unspecified Causes	213,808.00 (102,550.81 to 353,084.76)	14.78 (7.09 to 24.43)	344,489.59 (191,443.92 to 532,727.09)	19.02 (10.58 to 29.40)	0.82 (0.78 to 0.86)
	**SDI**					
Incidence	High SDI	21,351.98 (8,408.65 to 39,948.03)	10.17 (3.98 to 18.99)	21,714.60 (9,523.09 to 39,304.15)	10.81 (4.73 to 19.51)	0.20 (0.14 to 0.26)
Incidence	High-middle SDI	45,698.10 (20,989.12 to 76,748.84)	15.87 (7.29 to 26.65)	42,592.01 (20,928.58 to 69,808.24)	18.08 (8.90 to 29.55)	0.43 (0.37 to 0.49)
Incidence	Middle SDI	102,708.52 (48,728.91 to 168,855.14)	20.07 (9.52 to 33.02)	148,600.15 (83,664.78 to 226,289.68)	27.28 (15.40 to 41.46)	1.00 (0.97 to 1.04)
Incidence	Low-middle SDI	64,835.99 (31,690.18 to 104,849.45)	20.94 (10.20 to 33.90)	136,768.19 (77,096.80 to 208,117.52)	26.27 (14.79 to 40.02)	0.73 (0.71 to 0.75)
Incidence	Low SDI	23,335.72 (11,749.85 to 37,442.52)	18.13 (9.07 to 29.19)	68,803.00 (38,352.71 to 103,803.73)	21.85 (12.05 to 33.15)	0.59 (0.57 to 0.62)
	**Region**					
Incidence	Andean Latin America	1,609.22 (673.17 to 2,783.66)	15.02 (6.24 to 26.05)	3,882.31 (1,883.66 to 6,340.62)	22.68 (11.07 to 36.98)	1.35 (1.31 to 1.39)
Incidence	Australasia	335.60 (90.03 to 697.85)	6.73 (1.80 to 13.99)	523.26 (163.38 to 1,028.25)	8.57 (2.65 to 16.86)	0.78 (0.69 to 0.87)
Incidence	Caribbean	2,345.71 (1,077.19 to 3,857.03)	23.06 (10.58 to 37.93)	3,954.50 (2,182.94 to 6,051.68)	35.05 (19.37 to 53.61)	1.41 (1.38 to 1.44)
Incidence	Central Asia	9,113.73 (5,373.76 to 13,391.95)	48.42 (28.60 to 71.07)	13,612.80 (8,724.50 to 19,312.79)	63.63 (41.20 to 89.54)	0.90 (0.85 to 0.95)
Incidence	Central Europe	4,035.11 (1,589.35 to 7,162.52)	14.89 (5.87 to 26.42)	3,658.02 (1,698.21 to 5,985.10)	19.31 (8.99 to 31.55)	0.84 (0.81 to 0.88)
Incidence	Central Latin America	12,635.32 (6,060.04 to 20,418.85)	26.87 (12.88 to 43.47)	29,693.71 (18,376.85 to 43,099.69)	46.60 (28.85 to 67.61)	1.80 (1.76 to 1.83)
Incidence	Central Sub-Saharan Africa	2,005.72 (904.06 to 3,364.14)	13.56 (6.02 to 22.87)	6,428.55 (2,996.98 to 10,344.78)	16.81 (7.69 to 27.26)	0.69 (0.64 to 0.73)
Incidence	East Asia	52,810.70 (21,856.56 to 92,904.79)	13.99 (5.81 to 24.62)	31,700.40 (13,399.96 to 55,002.12)	12.70 (5.35 to 22.04)	−0.29 (−0.36 to −0.23)
Incidence	Eastern Europe	12,698.46 (5,991.05 to 21,112.98)	25.55 (12.04 to 42.49)	11,542.54 (5,840.30 to 18,381.76)	35.42 (17.91 to 56.30)	1.05 (1.00 to 1.11)
Incidence	Eastern Sub-Saharan Africa	6,518.59 (3,052.23 to 10,858.28)	12.72 (5.85 to 21.33)	17,406.10 (8,761.42 to 26,891.88)	13.83 (6.81 to 21.56)	0.26 (0.19 to 0.32)
Incidence	High-income Asia Pacific	4,830.47 (1,673.87 to 8,897.42)	11.54 (4.01 to 21.25)	3,023.82 (1,084.51 to 5,544.56)	10.46 (3.72 to 19.18)	−0.36 (−0.51 to −0.21)
Incidence	High-income North America	6,905.21 (2,400.42 to 13,593.96)	10.32 (3.55 to 20.30)	6,462.40 (2,401.89 to 12,648.45)	8.75 (3.23 to 17.12)	−0.53 (−0.56 to −0.49)
Incidence	North Africa and Middle East	16,545.11 (7,431.15 to 27,874.90)	17.97 (8.04 to 30.35)	40,499.42 (19,982.91 to 66,219.19)	26.48 (13.07 to 43.29)	1.25 (1.21 to 1.29)
Incidence	Oceania	610.38 (338.69 to 918.32)	33.46 (18.53 to 50.39)	1,405.63 (846.61 to 2,051.24)	37.93 (22.84 to 55.37)	0.41 (0.34 to 0.48)
Incidence	South Asia	62,285.30 (30,111.94 to 100,360.76)	21.42 (10.35 to 34.54)	128,148.50 (70,152.98 to 195,246.27)	25.24 (13.81 to 38.48)	0.51 (0.39 to 0.63)
Incidence	Southeast Asia	33,069.26 (16,431.22 to 52,345.15)	24.77 (12.28 to 39.25)	57,422.66 (33,334.68 to 85,735.91)	33.82 (19.69 to 50.42)	1.01 (0.98 to 1.04)
Incidence	Southern Latin America	904.50 (238.89 to 1,925.93)	7.40 (1.96 to 15.75)	1,337.93 (407.58 to 2,512.58)	8.40 (2.55 to 15.78)	0.42 (0.31 to 0.53)
Incidence	Southern Sub-Saharan Africa	3,933.15 (2,004.63 to 6,144.18)	26.79 (13.72 to 41.79)	5,694.50 (3,045.66 to 8,708.37)	27.14 (14.50 to 41.52)	0.03 (−0.04 to 0.10)
Incidence	Tropical Latin America	7,182.17 (2,981.71 to 12,623.47)	16.84 (7.01 to 29.58)	9,329.16 (3,943.04 to 16,022.44)	17.49 (7.35 to 30.05)	0.12 (0.09 to 0.15)
Incidence	Western Europe	5,769.44 (1,665.97 to 12,018.16)	6.40 (1.83 to 13.32)	4,745.61 (1,477.67 to 9,630.00)	6.33 (1.96 to 12.84)	−0.03 (−0.08 to 0.01)
Incidence	Western Sub-Saharan Africa	12,036.95 (6,138.25 to 19,033.26)	23.88 (12.09 to 37.89)	38,394.13 (21,473.92 to 58,175.86)	28.39 (15.67 to 43.26)	0.55 (0.52 to 0.57)

### Global trends by sex

From 1990 to 2021, the ASIR and ASPR of CKD among adolescents and emerging adults increased globally in both males and females, with faster increases observed in males (incidence: AAPC 1.12% vs. 0.58%; prevalence: AAPC 0.27% vs. 0.18%) (Table 1, Supplementary Table S1). In contrast, females exhibited stable trends in ASMR and ASDR during the same period (mortality: AAPC −0.15%, 95% UI: −0.35%–0.06%; DALYs: AAPC −0.17%, 95% UI: −0.38%–0.04%), whereas males experienced continued increases in ASMR and ASDR (mortality: AAPC 0.4%, 95% UI: 0.34%–0.46%; DALYs: AAPC 0.32%, 95% UI: 0.28%–0.36%) (Supplementary Table S2–3). By 2021, the global CKD burden in males had significantly surpassed that in females, and the sex disparity has been widening.

### Global trends by age

During the period from 1990 to 2021, the ASIR of CKD increased globally across all adolescent and emerging adult age subgroups, particularly in the 15–19 age group (AAPC 1.19%) (Table 1). It rose from 15.4 per 100,000 people in 1990 to 22.22 per 100,000 people in 2021. Although still lower than in the 25–29 age group, the incidence among those aged 15–19 has surpassed that of the 20–24 age group. ASPR similarly increased across all age subgroups, with the greatest increase observed in the 20–24 age group (AAPC 0.26%) (Supplementary Table S1).

From 1990 to 2021, ASMR and ASDR showed the most significant rise in the 25–29 age group (mortality: AAPC 0.25%, DALYs: AAPC 0.18%). In 2021, the 25–29 age group exhibited the highest ASIR, ASPR, ASMR, and ASDR, with values of 25.2 (95% UI: 13.08–41.18), 6221.49 (95% UI: 4746.15–7877.27), 2.54 (95% UI: 2.3–2.8), and 201.5 (95% UI: 177.1–229.15) per 100,000 population, respectively (Supplementary Table S2–3).

### Global trends by cause

The GBD 2021 classified CKD into five etiological categories: diabetes mellitus type 1, diabetes mellitus type 2, hypertension, glomerulonephritis, and ‘other and unspecified causes’.

In 2021, CKD due to ‘other and unspecified causes’ exhibited the highest ASIR and ASPR (incidence: 19.02 per 100,000 people; prevalence: 3371.41 per 100,000 people), followed by CKD due to glomerulonephritis and diabetes mellitus type 2 (Table 1, Supplementary Table S1). Notably, while CKD due to diabetes mellitus type 2 showed the largest decrease in both ASIR and ASPR (incidence: AAPC −0.85%; prevalence: AAPC −1.03%), CKD due to diabetes mellitus type 1 demonstrated the greatest increase (incidence: AAPC 1.19%; prevalence: AAPC 1.54%). As regards mortality and DALYs, CKD due to glomerulonephritis led to the highest ASMR and ASDR (mortality: 0.85 per 100,000 people; DALYs: 61.84 per 100,000 people), significantly surpassing those caused by diabetes mellitus (Supplementary Table S2–3). Furthermore, CKD due to glomerulonephritis experienced the largest increase in these metrics (mortality: AAPC 0.66%; DALYs: AAPC 0.58%), whereas CKD due to diabetes mellitus saw the greatest reduction. Overall, in 2021, glomerulonephritis and ‘other and unspecified causes’ contributed 93.9% of incident cases, 83% of prevalent cases, 92% of deaths and 90% of DALYs.

### SDI trends

Between 1990 and 2021, the ASIR of CKD increased across all adolescent and emerging adult subgroups categorized by the SDI. Similarly, the ASPR of CKD showed an increase in all SDI subgroups except for high SDI regions (Table 1, Supplementary Table S1). Notably, the middle SDI region exhibited the most significant upward trends in both ASIR and ASPR, with AAPC values of 1.0% and 0.29%, respectively. By 2021, the middle SDI region reported the highest ASIR, at 27.28 (95% UI: 15.4–41.46) per 100,000 population, while low-middle SDI regions reported the highest ASPR, at 4742.06 (95% UI: 3618.72–6077.74) per 100,000 population.

Regarding mortality and DALYs, in 2021, low SDI regions had the highest ASMR and ASDR (Supplementary Table S2–3), with values of 3.46 and 259.52 per 100,000 population, respectively. However, long-term trends indicate a decline in both ASMR and ASDR in low SDI regions, with AAPC values of −0.22% and −0.24%, respectively. Overall, middle SDI, low-middle SDI, and low SDI regions accounted for 85% of incident cases, 83% of prevalent cases, 92% of deaths, and 90% of DALYs in 2021.

### Region trends

Most regions experienced an increase in ASIR from CKD among adolescents and emerging adults at various rates during 1990–2021. However, a large reduction in ASIR was found in high-income North America (AAPC −0.53%). The largest increase in ASIR was in Central Latin America (AAPC 1.80%). In 2021, the highest ASIR was in Central Asia (63.63 per 100,000 population). The lowest ASIR was in Western Europe (6.33 per 100,000 people). The ASPR of CKD among adolescents and emerging adults was also different between regions. Southeast Asia, South Asia, and North Africa and the Middle East were the top three regions with the highest ASPR, with rates of 5,370.39, 5,351.64, and 4,734.96 per 100,000 people (Table 1, Supplementary Table S1).

From 1990 to 2021, the ASMR decreased in 14 regions, and the ASDR declined in 12 regions. In the high-income Asia Pacific region, a significant downward trend was observed in both ASMR and ASDR (mortality: AAPC −3.43%; DALYs: AAPC −2.13%), while high-income North America continued to observe the highest increases in ASPR and ASDR (mortality: AAPC 1.63%; DALYs: AAPC 1.08%). In 2021, Central Sub-Saharan Africa, Eastern Sub-Saharan Africa, and Western Sub-Saharan Africa were the top three regions with the highest ASMR at 5.05, 4.57, and 4.09 per 100,000 people, respectively. Similarly, these three regions also reported the highest ASDR, with rates of 368.11, 325.21, and 304.76 per 100,000 people (Supplementary Table S2–3).

### Country trends

**Figure 1.**
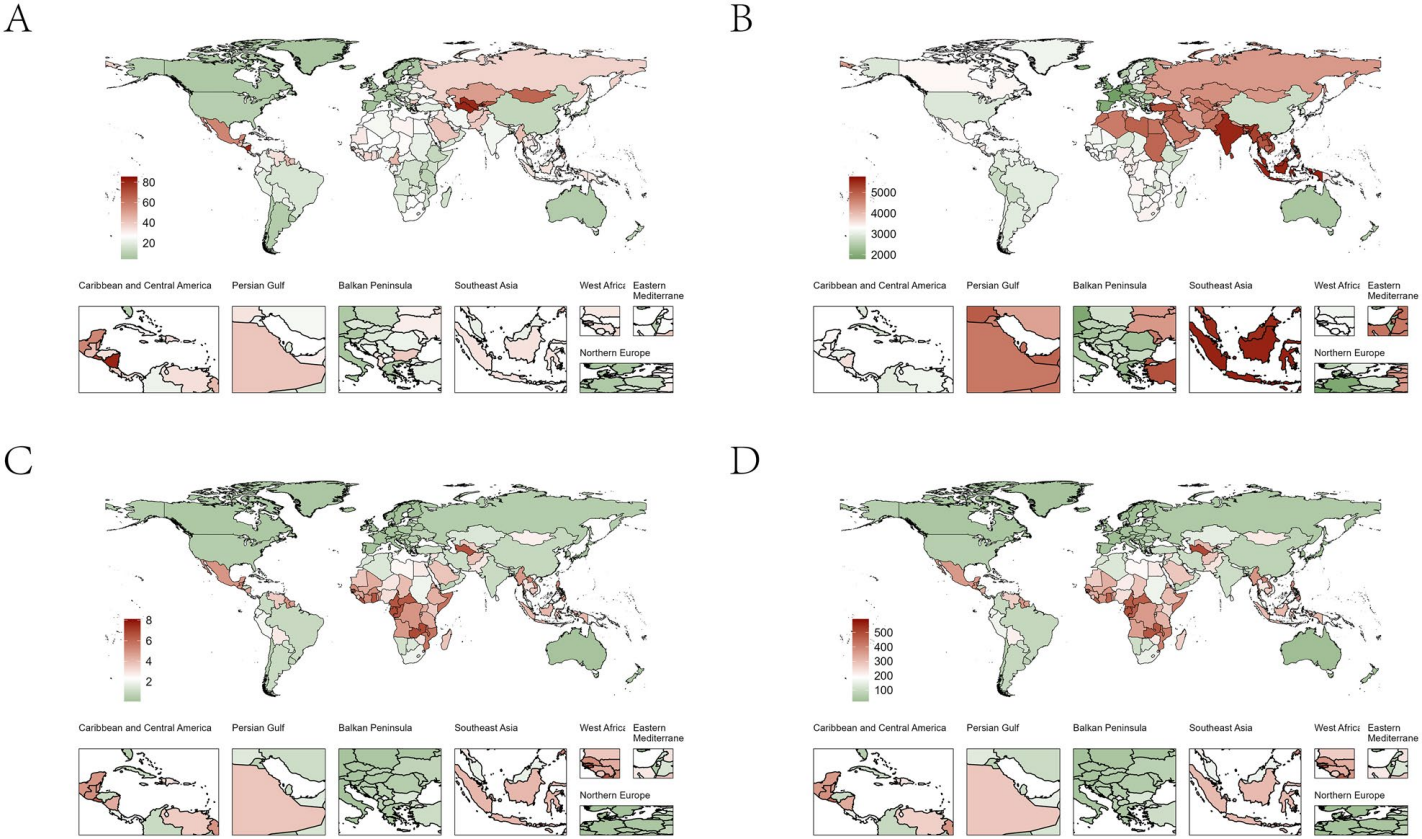
Global map of age-standardized rates for chronic kidney Disease among adolescents and emerging adults in 2021. (A) ASIR. (B) ASPR. (C) ASMR. (D) ASDR.

At the national level, the burden of CKD among adolescents and emerging adults was increased in most countries. From 1990 to 2021, about 90% of countries saw an increase in the ASIR, approximately 63% of countries experienced an increase in ASPR, and around 34% of countries observed increasing trends in both ASMR and ASDR. El Salvador had the highest increase in ASIR (AAPC 2.87%). Guatemala had the highest increase in ASPR (AAPC 0.43%) (Figure 1, Supplementary Tables S1 and S4, Supplementary Figure S2). In 2021, Palau had the highest ASIR of CKD among adolescents and emerging adults (85.45 per 100,000 people), while Mauritius had the highest ASPR (5,758.15 per 100,000 people). During the same period, Ukraine showed the most pronounced increase in ASMR and ASDR of CKD among adolescents and emerging adults (mortality: AAPC 8.88%; DALYs: AAPC 4.07%). In 2021, Niue had the highest ASMR and ASDR of CKD among adolescents and emerging adults (mortality: 8.11 per 100,000 people; DALYs: 592.72 per 100,000 people) (Supplementary Tables S2–S3).

### Cross-country inequality analysis

**Figure 2.**
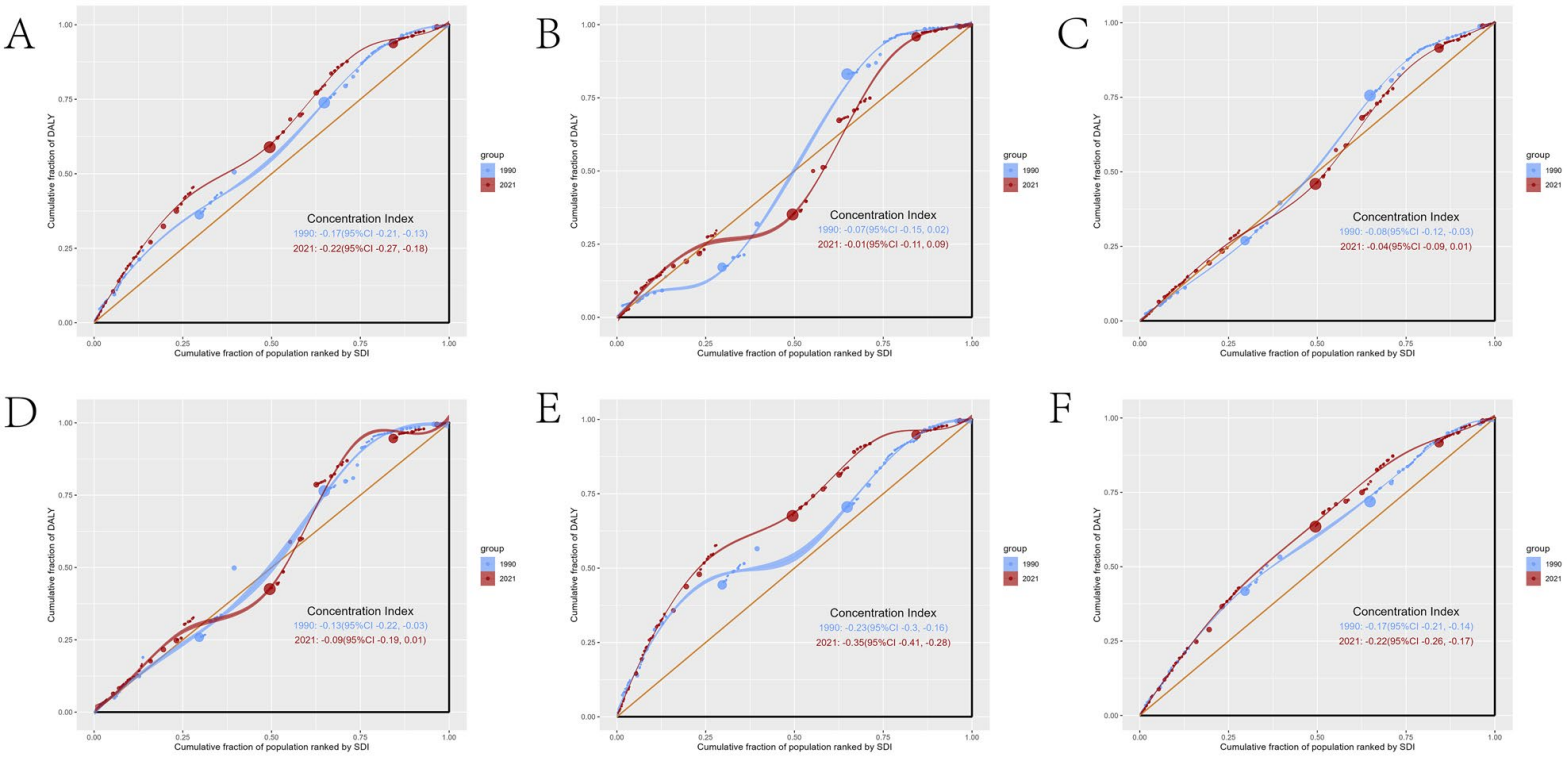
Relative concentration Index (RCI) for health inequality analysis of chronic kidney Disease in adolescents and emerging adults. A: Chronic kidney Disease including all causes; B: Chronic kidney Disease caused by diabetes mellitus type 1; C: Chronic kidney Disease caused by diabetes mellitus type 2; D: Chronic kidney Disease caused by hypertension; E: Chronic kidney Disease caused by glomerulonephritis; F: Chronic kidney Disease caused by other and unspecified causes.

We first assessed health inequalities in the burden of all-cause CKD among adolescents and emerging adults. Subsequently, we evaluated inequalities in CKD burden attributable to different etiologies (Figure 2, Supplementary Figure S3).

On a global scale, significant health inequalities in CKD burden were observed among adolescents and emerging adults in 2021, with these inequalities predominantly concentrated in countries with low SDI. Compared to 1990, health inequalities have increased, as evidenced by the rise in the SII from −212 in 1990 to −230 in 2021, and the RCI from −0.13 to −0.19. The health inequality trends varied across different etiologies of CKD. The RCI for CKD due to type 1 diabetes decreased from −0.07 in 1990 to −0.01 in 2021, while for type 2 diabetes, it decreased from −0.08 to −0.04, and for hypertension, from −0.17 to −0.08. These trends suggest a reduction in health inequality associated with CKD burden due to diabetes and hypertension. In contrast, the RCI for CKD due to glomerulonephritis increased from −0.22 in 1990 to −0.35 in 2021, and for CKD of ‘other and unspecified causes’, it increased from −0.18 to −0.22. These findings indicate an increase in health inequality related to CKD burden due to glomerulonephritis and ‘other and unspecified causes’, with both etiologies surpassing an RCI of −0.2 in 2021, reflecting a significant degree of health inequality.

### Frontier analysis

**Figure 3.**
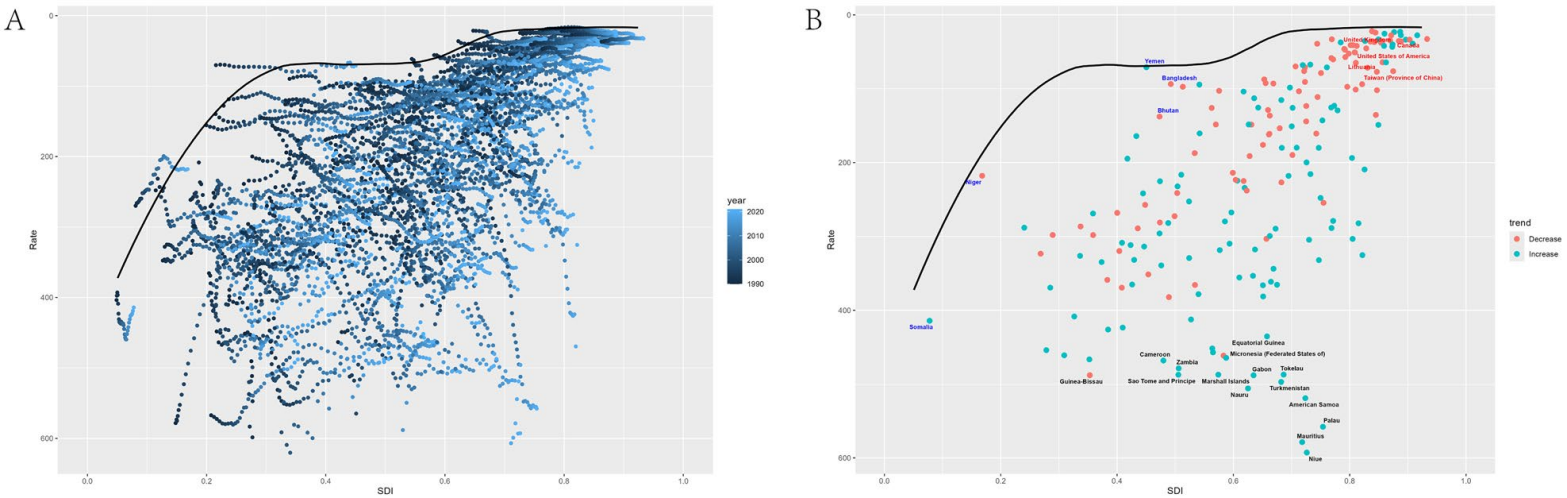
Frontier analysis of chronic kidney Disease in adolescents and emerging adults. (A) Frontier analysis based on SDI and ASDR from 1990 to 2021. Color scale represents the years from 1990 depicted in light blue to 2021 depicted in deep blue. The frontier is delineated in solid black color. (B) Frontier analysis based on SDI and ASDR in 2021. The frontier is delineated in solid black color; countries and territories are represented as dots. The top 15 countries with the largest effective difference (largest Chronic Kidney Disease ASDR gap from the frontier) are labeled in black; examples of frontier countries with low SDI (<0.5) and low effective difference are labeled in blue (e.g., Yemen, Somalia, Niger, Bangladesh, and Bhutan), and examples of countries and territories with high SDI (>0.85) and relatively high effective difference for their level of development are labeled in red (e.g., United States of America, Taiwan (Province of China), United Kingdom, Canada, and Lithuania). Red dots indicate an increase in ASDR from 1990 to 2021; blue dots indicate a decrease in ASDR between 1990 and 2021.

To better understand the potential improvement in CKD DALYs that are potentially achievable given a country’s development status, we built a frontier analysis based on ASDR and SDI using data from 1990 to 2021. The boundary represents the minimum achievable ASDR across various SDI levels (Figure 3, Supplementary Table S5). The distance from this boundary is referred to as the ‘effective difference’, which reflects the gap between the observed DALYs and the potentially achievable DALYs in a country; this gap may be reduced or eliminated depending on the socio-demographic resources available to the country or region. Overall, as SDI increases, the effective difference for a given SDI tends to become smaller. The ten countries with the largest effective differences from the frontier (range: 419.58–570.63) include Niue, Mauritius, Palau, American Samoa, Turkmenistan, Tokelau, Nauru, Gabon, Marshall Islands, and São Tomé and Príncipe. The top eight of these are either high-middle or middle SDI countries. In contrast, the ten countries with the smallest DALYs effective differences (range: 3.8–11.18) are Yemen, France, Sweden, Iceland, Republic of Korea, Australia, Finland, San Marino, Norway, and Japan, with all but Yemen being high SDI countries.

When stratified by SDI, in high SDI regions, the 3 countries with the smallest effective DALY differences are Iceland, Sweden, and France, while the 3 countries with the largest effective DALY differences are the United States Virgin Islands, Saudi Arabia, and Puerto Rico. In high-middle SDI regions, the 3 countries with the smallest effective DALY differences are Belarus, Portugal, and Spain, while the 3 countries with the largest effective DALY differences are Palau, Mauritius, and Niue. In middle SDI regions, the 3 countries with the smallest effective DALY differences are Albania, Brazil, and Colombia, while the 3 countries with the largest effective DALY differences are Nauru, Tokelau, and Turkmenistan. In low-middle SDI regions, the 3 countries with the smallest effective DALY differences are Honduras, Tajikistan, and Bangladesh, while the 3 countries with the largest effective DALY differences are Belarus, Sao Tome and Principe, and Zambia. In low SDI regions, the 3 countries with the smallest effective DALY differences are Yemen, Niger, and Somalia, while the 3 countries with the largest effective DALY differences are Guinea-Bissau, Liberia, and the Central African Republic.

### Risk factor

**Figure 4.**
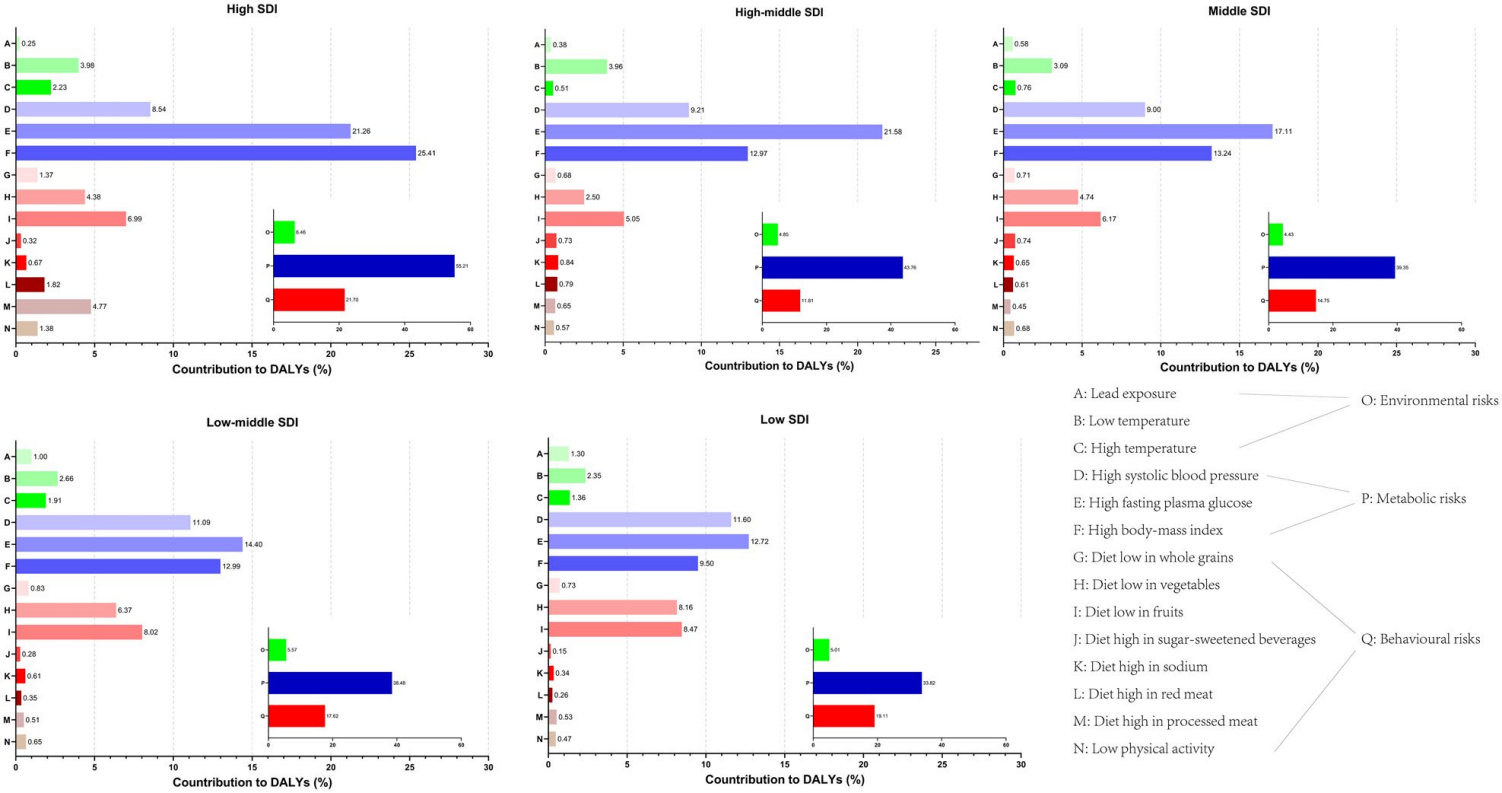
Percentage contribution of risk factors to DALYs of chronic kidney Disease among aged 25 to 29 in 2021 by SDI.

The GBD study classified all risk factors into four hierarchical levels, with the first level comprising environmental, behavioral, and metabolic risks. These first-level risk factors are further divided into second, third, and fourth levels. In this study, for the 15–19 and 20–24 age groups, GBD 2021 provides DALYs data only for risks associated with high and low temperatures. In contrast, for the 25–29 age group, data are available for 14 risk factors, including 8 behavioral risks, 3 metabolic risks, and 3 environmental risks. We therefore focused on the 25–29-year age group. Figure 4 illustrates the contribution of distinct risk factors to CKD-related DALYs across five SDI regions within this age group.

In the 25–29 age group, metabolic risk factors, including high BMI, hyperglycemia, and hypertension, are the primary contributors to CKD-related DALYs across all regions. In high-SDI regions, high BMI is the leading contributor to CKD DALYs, accounting for 25.41%. Conversely, in the other four regions, hyperglycemia contributes the most to CKD DALYs. Behavioral risks are secondary risk factors, with low intake of vegetables and fruits being the primary behavioral risk factor across all regions. In high-SDI regions, processed meat consumption also contributes significantly to CKD DALYs, accounting for 4.77% (Figure 4). Moreover, we observed a significant regional differential effect of risk factors on CKD DALYs. In low-SDI regions, hypertension, low vegetable intake, and low fruit intake contribute more prominently to CKD burden, while in high-SDI regions, high BMI, red meat consumption, and processed meat intake have a stronger impact. Of these, high BMI, red meat consumption, and processed meat intake contribute 2.67, 7, and 9 times more to CKD DALYs in high-SDI regions than in low-SDI regions, respectively.

## Discussion

This study provides a comprehensive assessment of the burden of CKD in adolescents and emerging adults. The primary findings are as follows: First, from 1990 to 2021, there was a significant increase in the ASIR, ASPR, and ASMR of CKD among adolescents and emerging adults worldwide. However, the overall ASDR remained stable during this period. In 2021, the prevalence of CKD among adolescents and emerging adults was primarily concentrated in regions such as Southeast Asia, South Asia, and the Middle East and North Africa, whereas mortality rates were highest in Sub-Saharan Africa. Second, glomerulonephritis and ‘other and unspecified causes’ were identified as the primary drivers of CKD burden in these populations. Third, the burden is more disproportionally borne by regions with low, low-middle, and middle SDI. Further analysis reveals that these inequalities are primarily driven by glomerulonephritis and ‘other and unspecified causes’. Fourth, significant potential remains for reducing CKD burden in most countries, especially in middle and high-middle SDI countries. Frontier analysis provides exemplars to identify the drivers of success at specific stages of development. Fifth, in regions with different SDI levels, there are significant regional variations in the attributable risk factors for CKD patients. In high-SDI regions, high BMI is the primary risk factor for CKD patients aged 25–29, whereas in the other four regions, hyperglycemia is considered the leading risk factor.

Although some findings of this study align with previous research [13] (e.g., increase in incidence, stable DALYs, and consistent sex- and age-specific patterns), this study provides novel perspectives. Regarding mortality, although the 2019 study reported a downward trend (Estimated Annual Percentage Change [EAPC]: −0.4%), this study found an increase in mortality (AAPC: 0.18%). This discrepancy may stem from several factors. First, differences in the modeling approach. The aforementioned study used the ‘Estimated Annual Percentage Change’ (EAPC) model, whereas this study employs the ‘average annual percentage change’ (AAPC) model. While both methods are used to describe trends in time-series data, they differ in how they handle annual fluctuations and trend stability. EAPC typically applies linear trend analysis within a single time interval, whereas AAPC accommodates multi-period data. This makes AAPC more robust for long-term trend analysis, particularly for health indicators with significant annual fluctuations [14–16]. For CKD mortality specifically, AAPC captures the upward trend in mortality since 2013 more accurately, whereas EAPC may underestimate short-term fluctuations in mortality trends. Therefore, the choice of calculation model directly impacts mortality change interpretations, with AAPC yielding more reliable results that reflect authentic mortality increase patterns. Another key factor is the estimation methodology of GBD data. GBD data are derived from statistical models and estimation methods rather than entirely raw observational data [9]. In remote regions (e.g., the Sahara Desert), GBD data often rely on indirect estimates. This means that as time progresses and new data are incorporated, the estimates in GBD may be updated and refined, leading to potential discrepancies between years.

This global study reveals CKD prevalence patterns among adolescents and emerging adults, with hyperendemic regions clustered in South/Southeast Asia (India, Sri Lanka, Malaysia, Indonesia) and select Middle Eastern/North African countries. This geographic distribution pattern is similar to that of CKD prevalence across all age groups [17]. Despite observed improvements in incidence rates in countries such as the Maldives and Thailand, the burden of CKD as an irreversible disease is projected to continue to increase across nations. In Southeast and South Asia, adolescents and emerging adults are also at risk of chronic kidney disease of undetermined etiology (CKDu). This condition has been reported in various regions under different names [18–20], including Mesoamerican Nephropathy, Sri Lankan Nephropathy, and Uddanam Nephro­pathy, all of which share similar characteristics: hot climates and long-term and high-intensity agricultural practices. Distinct from conventional CKD etiologies (diabetes, hypertension, glomerulopathies), CKDu pathogenesis remains unclear. Current hypotheses suggest that heat stress, dehydration, heavy metals, and agricultural chemicals may be potential causes [21–23]. Adolescents and emerging adults, whose physical development is not yet complete, may be more susceptible to these nephrotoxic effects. Thus, in these high-risk regions, the focus of primary and secondary prevention strategies should be on mitigating heat-related health risks and reducing exposure to nephrotoxic agents. For instance, reducing exposure to high temperatures and sunlight, ensuring safe water sources, and improving hydration to restore electrolytes can effectively slow the decline of eGFR during the harvest period [24]. Additionally, government policies that impose bans on nephrotoxic pesticides can play a significant role in mitigating the spread of CKDu.

Our study reveals that glomerulonephritis and ‘other and unspecified causes’ are the leading contributors to the burden of CKD in adolescents and emerging adults, and they are also key drivers of health inequalities. Based on DALYs, from 1990 to 2021, health inequities related to CKD among adolescents and emerging adults have intensified. Notably, health inequities driven by CKD related to diabetes and hypertension have shown signs of narrowing. However, inequities caused by glomerulonephritis and ‘other and unspecified causes’ of CKD have increased, and by 2021, the RCI for CKD burden from these two etiologies exceeded 0.2. In practice, an absolute value of the RCI exceeding 0.2 is considered to represent a relatively high level of relative inequality. A key factor contributing to this phenomenon may be the disparity in access to treatment modalities for different underlying causes of CKD. Diagnosis of glomerular diseases typically relies on kidney biopsy. However, in resource-limited regions, kidney pathology services are severely limited, particularly for immunofluorescence staining and electron microscopy [25]. The lack of specialized diagnostic techniques makes it difficult to diagnose glomerular diseases, thereby affecting subsequent treatment and management. In contrast, hypertensive nephropathy or diabetic nephropathy is less challenging to diagnose and treat, as it depends on the management of the underlying conditions, for which treatment options are more readily available [26–27]. Furthermore, as diabetes and hypertension are key global health priorities, especially under the Sustainable Development Goals (SDGs) framework [28], these conditions are more likely to benefit from policy interventions. Although these policies primarily target adults aged 30 to 70, their implementation undoubtedly exerts a positive influence on the management of diabetes and hypertension across broader populations, indirectly contributing to CKD prevention. Therefore, integrating renal biopsy into national healthcare initiatives or international programs would undeniably serve as an invaluable asset in the effective management of kidney diseases.

We conducted a frontier analysis to assess the DALYs associated with CKD among adolescents and emerging adults in countries with similar resources. This analysis quantitatively compared each country’s potential for mitigating the burden of CKD under varying socio-economic conditions. Our findings revealed that adolescent and emerging adult populations with CKD in high-SDI regions exhibited favorable outcomes in terms of DALYs. In contrast, a significant gap between ideal DALYs and actual DALYs was observed in regions with middle SDI or high-middle SDI, suggesting considerable potential for improvement. Notably, countries such as Niue, Mauritius, Palau, American Samoa, and Turkmenistan, which have made some progress in economic development, demonstrated a relatively large gap from the ideal DALYs, with underutilization of medical resources potentially contributing to this significant disparity. Some countries with similar SDI levels displayed leading frontier performance in this analysis. Albania, Brazil, Cuba, and Colombia, for instance, were among the countries with the smallest effective gap in the middle SDI group. These nations can serve as exemplars for optimizing health outcomes in resource-scarce settings. For example, Brazil and Cuba have developed national healthcare models specifically designed for the prevention and management of chronic kidney disease [29–31]. These include initiatives such as enhancing CKD education for primary care physicians, strengthening the linkage between primary healthcare providers and nephrologists, and establishing CKD registries to track related complications. The healthcare policies in these countries provide a framework for how health outcomes can be optimized through innovative and effective policy interventions in resource-constrained environments.

Risk factors for CKD patients show significant regional differences across different SDI levels. High BMI is the most important factor contributing to CKD DALYs in high-SDI regions, while hyperglycemia is the main contributor to CKD DALYs in the other four regions. In terms of diet, red meat and processed meat are key risk factors in high-SDI regions, whereas low intake of fruits and vegetables is a significant contributing factor to CKD DALYs in low-SDI regions. Numerous studies have shown that individuals who are overweight or obese have a significantly higher risk of developing CKD compared to those with a normal BMI [32–34]. This risk remains evident even in patients with the ‘metabolically healthy’ obesity phenotype [35–36]. Research indicates that approximately 14% of men and 25% of women in industrialized countries develop CKD due to the clinical consequences of being overweight or obese [37]. This underscores the necessity of controlling obesity rates and promoting weight management in high-SDI regions. Additionally, the consumption of red meat and processed meat is higher in high-SDI regions [38]. Several observational studies have shown that the intake of red meat and processed meats is associated with an increased risk of CKD [39–40], and replacing red and/or processed meat has been linked with reductions in CKD risk [41]. Although the specific mechanisms remain incompletely understood, this suggests that adopting a dietary pattern focused on reducing red meat and processed meats intake may serve as a primary and secondary preventive measure for CKD in high-SDI regions. Overall, health policies need to be tailored to the specific conditions of different regions in order to more effectively reduce the disease burden caused by CKD.

There are several limitations to this study. First, this study is based on the GBD 2021 database, which makes it hard to guarantee that our results are absolutely accurate, as some values are estimates rather than direct measurements, despite using more advanced statistical methods in GBD 2021. In some low-income and middle-income countries, the estimates from GBD rely heavily on modeling processes, predictive covariates, and trends from past data or neighboring countries. Second, due to the lack of data, we were unable to analyze the relationship between CKD stage and age-standardized rate. Third, for other common causes of CKD, such as congenital anomalies of the kidney and urinary tract and hereditary kidney diseases, GBD 2021 did not provide detailed differentiation. Therefore, further high-quality clinical research are required to validate the results of this study.

## Conclusion

There still remain large disease burdens of CKD among adolescents and emerging adults worldwide. The burden varies significantly by region, particularly in sub-Saharan Africa and Southeast Asia, where glomerulonephritis is the primary driver of health inequities. However, our analysis reveals that several resource-limited countries have a leading performance for CKD DALYs, potentially serving as models for other nations. While metabolic and behavioral factors remain the primary risk factors globally, the distribution and patterns of these risks exhibit significant regional variation. The findings underscore the urgent need for governments to develop targeted intervention strategies to address the growing burden of CKD among adolescents and emerging adults.

## Supplementary Material

Supplementary Table S2.docx

F1 A.tif

S3.jpg

S2 A.tif

S2.tif

F2 C.tif

Supplementary Table S3.docx

S2 C.tif

F3 B.tif

S3 B.tif

S1 B.tif

F1 B.tif

F2 D.tif

F3 A.tif

S3 C.tif

S1 D.tif

F2 E.tif

F1 C.tif

S2 D.tif

F1 D.tif

S1 A.tif

Supplementary Table S5.docx

S3 F.tif

S2 B.tif

F2 F.tif

S3 A.tif

Supplementary Table S1.docx

F2 B.tif

Supplementary Table S4.docx

S1 C.tif

S3 D.tif

F2 A.tif

S1.tif

S3 E.tif

## Data Availability

The datasets analyzed during the current study are available in the GBD Results Tool repository, https://ghdx.healthdata.org/gbd-2021.

## List of Abbreviations

CKD: Chronic kidney disease
ASIR: Age-standardized incidence
ASPR: Age-standardized prevalence
ASMR: Age-standardized mortality
DALYs: Disability-adjusted life years
ASDR: Age-standardized DALYs
SDI: Sociodemographic index
YLL: Years of life lost
YLDs: Years lived with disability
95% UI: 95% uncertainty intervals
APC: Annual percentage change
AAPC: Average annual percentage change
SII: Slope Index of Inequality
RCI: Relative Concentration Index
DEA: Data Envelopment Analysis
EAPC: Estimated Annual Percentage Change
