# Supplementary material for: Global, regional, and national burden of chronic kidney disease among adolescents and emerging adults from 1990 to 2021
Source: Ren Fail. 2025 May 22;47(1):2508296. doi: 10.1080/0886022X.2025.2508296 (PMC12101043; doi:10.1080/0886022X.2025.2508296)
Supplement: Supplementary Table S2.docx [file IRNF_A_2508296_SM3083.docx]

Supplementary Table S2 Global Deaths of CKD and Their AAPCs From 1990 to 2021 by Sex, Age, Cause, SDI, Region and Country in Adolescents and Emerging adults

| **Measure** | **Variable** | 1990 | | 2021 | | AAPC (95% UI) |
| --- | --- | --- | --- | --- | --- | --- |
|  |  | Number (95% UI) | ASR (95% UI) | Number (95% UI) | ASR (95% UI) |  |
| Deaths | Global | 28,000.35 (24,519.69 to 31,454.59) | 1.93 (1.70 to 2.17) | 37,052.19 (33,022.23 to 41,245.56) | 2.04 (1.82 to 2.27) | 0.18 (0.04 to 0.32) |
|  | **Sex** | | | | | |
| Deaths | Male | 15,560.73 (12,412.93 to 18,075.80) | 2.13 (1.70 to 2.47) | 22,181.99 (18,758.98 to 25,393.35) | 2.41 (2.03 to 2.75) | 0.40 (0.34 to 0.46) |
| Deaths | Female | 12,439.62 (11,040.69 to 14,200.94) | 1.74 (1.54 to 1.98) | 14,870.20 (13,017.30 to 16,988.38) | 1.67 (1.46 to 1.90) | -0.15 (-0.35 to 0.06) |
|  | **Age** | | | | | |
| Deaths | 15-19 | 7,881.48 (6,750.49 to 8,845.87) | 1.52 (1.30 to 1.70) | 9,513.75 (8,291.82 to 10,782.62) | 1.52 (1.33 to 1.73) | -0.01 (-0.09 to 0.07) |
| Deaths | 20-24 | 9,725.36 (8,492.76 to 10,885.32) | 1.98 (1.73 to 2.21) | 12,580.68 (11,185.82 to 13,994.04) | 2.11 (1.87 to 2.34) | 0.20 (0.09 to 0.30) |
| Deaths | 25-29 | 10,393.52 (9,276.44 to 11,723.41) | 2.35 (2.10 to 2.65) | 14,957.76 (13,544.58 to 16,468.90) | 2.54 (2.30 to 2.80) | 0.25 (0.07 to 0.44) |
|  | **Cause** | | | | | |
| Deaths | Diabetes mellitus type 1 | 4,777.95 (2,938.60 to 6,933.91) | 0.33 (0.21 to 0.48) | 4,228.42 (2,498.04 to 6,330.23) | 0.23 (0.14 to 0.35) | -1.17 (-1.44 to -0.89) |
| Deaths | Diabetes mellitus type 2 | 122.31 (39.71 to 286.73) | 0.01 (0.00 to 0.02) | 123.64 (39.63 to 293.02) | 0.01 (0.00 to 0.02) | -0.88 (-1.02 to -0.74) |
| Deaths | Hypertension | 5,035.35 (3,015.01 to 7,619.16) | 0.35 (0.21 to 0.53) | 5,879.16 (3,530.69 to 8,760.18) | 0.32 (0.19 to 0.48) | -0.26 (-0.37 to -0.15) |
| Deaths | Glomerulonephritis | 9,783.21 (6,842.05 to 13,277.26) | 0.67 (0.47 to 0.91) | 14,954.08 (10,630.72 to 20,053.63) | 0.83 (0.59 to 1.11) | 0.66 (0.51 to 0.82) |
| Deaths | Other and unspecified causes | 8,281.54 (5,403.99 to 11,336.97) | 0.57 (0.37 to 0.78) | 11,866.89 (8,153.42 to 16,201.87) | 0.66 (0.45 to 0.89) | 0.46 (0.32 to 0.60) |
|  | **SDI** | | | | | |
| Deaths | High SDI | 1,042.82 (976.34 to 1,123.16) | 0.49 (0.46 to 0.53) | 1,066.67 (901.96 to 1,253.02) | 0.52 (0.44 to 0.61) | 0.22 (-0.15 to 0.59) |
| Deaths | High-middle SDI | 3,873.44 (3,484.00 to 4,420.63) | 1.34 (1.21 to 1.53) | 2,020.85 (1,796.08 to 2,337.71) | 0.85 (0.75 to 0.98) | -1.48 (-1.69 to -1.28) |
| Deaths | Middle SDI | 11,759.19 (9,884.30 to 13,241.44) | 2.30 (1.94 to 2.59) | 11,543.75 (10,186.22 to 12,591.55) | 2.10 (1.85 to 2.29) | -0.30 (-0.49 to -0.10) |
| Deaths | Low-middle SDI | 6,574.36 (5,607.27 to 7,742.66) | 2.14 (1.83 to 2.52) | 11,681.94 (10,139.21 to 13,349.51) | 2.25 (1.95 to 2.57) | 0.20 (0.03 to 0.36) |
| Deaths | Low SDI | 4,725.81 (3,895.89 to 5,510.99) | 3.69 (3.05 to 4.31) | 10,705.06 (8,815.53 to 13,057.91) | 3.46 (2.85 to 4.22) | -0.22 (-0.31 to -0.12) |
|  | **Region** | | | | | |
| Deaths | Andean Latin America | 298.67 (253.19 to 355.53) | 2.83 (2.39 to 3.36) | 384.81 (302.44 to 481.09) | 2.22 (1.75 to 2.78) | -0.85 (-1.72 to 0.03) |
| Deaths | Australasia | 9.90 (9.10 to 10.70) | 0.20 (0.18 to 0.21) | 11.28 (10.21 to 12.27) | 0.18 (0.16 to 0.20) | -0.30 (-0.97 to 0.37) |
| Deaths | Caribbean | 208.37 (181.59 to 249.74) | 2.06 (1.79 to 2.46) | 319.03 (249.33 to 448.05) | 2.81 (2.20 to 3.96) | 1.08 (0.42 to 1.74) |
| Deaths | Central Asia | 335.29 (305.77 to 370.66) | 1.77 (1.61 to 1.96) | 604.62 (525.66 to 687.83) | 2.70 (2.35 to 3.08) | 1.18 (0.30 to 2.07) |
| Deaths | Central Europe | 273.07 (260.51 to 287.74) | 1.01 (0.96 to 1.06) | 91.45 (78.25 to 110.04) | 0.48 (0.41 to 0.57) | -2.39 (-2.74 to -2.03) |
| Deaths | Central Latin America | 1,291.69 (1,249.71 to 1,346.09) | 2.77 (2.68 to 2.88) | 2,404.29 (2,156.07 to 2,696.22) | 3.76 (3.37 to 4.21) | 0.97 (0.78 to 1.16) |
| Deaths | Central Sub-Saharan Africa | 742.00 (539.30 to 984.32) | 5.06 (3.69 to 6.71) | 1,884.73 (1,305.27 to 2,611.15) | 5.05 (3.49 to 7.00) | -0.04 (-0.27 to 0.19) |
| Deaths | East Asia | 6,897.80 (5,767.88 to 8,025.70) | 1.82 (1.52 to 2.12) | 2,367.22 (1,839.91 to 2,920.26) | 0.94 (0.74 to 1.16) | -2.10 (-2.45 to -1.76) |
| Deaths | Eastern Europe | 632.28 (616.82 to 653.21) | 1.26 (1.23 to 1.30) | 185.66 (161.75 to 218.89) | 0.56 (0.49 to 0.66) | -2.61 (-3.20 to -2.02) |
| Deaths | Eastern Sub-Saharan Africa | 2,602.03 (2,091.79 to 3,066.75) | 5.15 (4.15 to 6.08) | 5,605.66 (4,569.50 to 6,900.15) | 4.57 (3.73 to 5.62) | -0.37 (-0.46 to -0.29) |
| Deaths | High-income Asia Pacific | 227.99 (190.53 to 258.94) | 0.55 (0.46 to 0.62) | 52.84 (48.39 to 61.37) | 0.18 (0.17 to 0.21) | -3.43 (-3.87 to -2.99) |
| Deaths | High-income North America | 210.26 (204.64 to 216.59) | 0.30 (0.30 to 0.31) | 365.72 (322.71 to 394.03) | 0.49 (0.43 to 0.52) | 1.63 (1.05 to 2.22) |
| Deaths | North Africa and Middle East | 1,725.87 (1,375.25 to 2,347.43) | 1.88 (1.50 to 2.55) | 2,975.31 (2,463.22 to 3,586.96) | 1.94 (1.61 to 2.34) | 0.14 (-0.10 to 0.39) |
| Deaths | Oceania | 40.09 (20.65 to 59.88) | 2.21 (1.14 to 3.30) | 104.89 (72.84 to 141.65) | 2.83 (1.97 to 3.82) | 0.87 (0.69 to 1.04) |
| Deaths | South Asia | 3,839.97 (3,286.77 to 4,689.59) | 1.33 (1.14 to 1.62) | 6,448.36 (5,332.75 to 8,116.89) | 1.27 (1.05 to 1.60) | -0.15 (-0.52 to 0.22) |
| Deaths | Southeast Asia | 5,321.04 (3,951.87 to 6,262.73) | 4.01 (2.99 to 4.72) | 6,629.54 (5,132.86 to 7,773.66) | 3.87 (2.99 to 4.54) | -0.12 (-0.22 to -0.02) |
| Deaths | Southern Latin America | 161.43 (149.66 to 175.18) | 1.32 (1.23 to 1.44) | 142.65 (129.28 to 154.42) | 0.89 (0.81 to 0.97) | -1.35 (-1.71 to -0.98) |
| Deaths | Southern Sub-Saharan Africa | 296.09 (247.68 to 378.71) | 2.05 (1.72 to 2.63) | 455.04 (375.12 to 574.05) | 2.17 (1.79 to 2.74) | -0.01 (-0.59 to 0.58) |
| Deaths | Tropical Latin America | 682.63 (650.48 to 722.01) | 1.60 (1.53 to 1.69) | 524.77 (490.29 to 553.74) | 0.98 (0.92 to 1.04) | -1.50 (-2.26 to -0.73) |
| Deaths | Western Europe | 201.66 (194.92 to 208.92) | 0.22 (0.21 to 0.23) | 117.87 (111.16 to 129.98) | 0.16 (0.15 to 0.17) | -1.06 (-1.83 to -0.29) |
| Deaths | Western Sub-Saharan Africa | 2,002.21 (1,528.54 to 2,554.78) | 4.04 (3.09 to 5.17) | 5,376.45 (3,944.66 to 7,088.78) | 4.09 (3.01 to 5.41) | 0.04 (-0.06 to 0.15) |
|  | **Country** | | | | | |
| Deaths | Afghanistan | 101.47 (64.24 to 163.11) | 4.51 (2.83 to 7.14) | 342.56 (188.16 to 590.50) | 3.80 (2.09 to 6.53) | -0.54 (-0.98 to -0.10) |
| Deaths | Albania | 11.69 (9.03 to 14.89) | 1.24 (0.96 to 1.58) | 3.90 (2.69 to 6.14) | 0.64 (0.44 to 1.01) | -2.31 (-3.23 to -1.38) |
| Deaths | Algeria | 107.33 (72.09 to 168.95) | 1.51 (1.01 to 2.38) | 146.23 (101.29 to 200.27) | 1.48 (1.02 to 2.03) | 0.03 (-0.30 to 0.37) |
| Deaths | American Samoa | 0.37 (0.23 to 0.54) | 2.64 (1.65 to 3.92) | 0.75 (0.50 to 1.03) | 7.03 (4.69 to 9.69) | 3.32 (2.96 to 3.69) |
| Deaths | Andorra | 0.04 (0.02 to 0.05) | 0.24 (0.16 to 0.34) | 0.03 (0.02 to 0.04) | 0.18 (0.12 to 0.26) | -0.99 (-1.37 to -0.62) |
| Deaths | Angola | 132.25 (86.38 to 193.82) | 4.86 (3.18 to 7.11) | 404.22 (260.59 to 609.62) | 4.80 (3.10 to 7.22) | 0.06 (-0.62 to 0.74) |
| Deaths | Antigua and Barbuda | 0.41 (0.36 to 0.46) | 2.38 (2.08 to 2.70) | 0.66 (0.57 to 0.77) | 3.17 (2.73 to 3.66) | 0.87 (-0.24 to 1.99) |
| Deaths | Argentina | 120.89 (109.96 to 133.63) | 1.57 (1.43 to 1.73) | 111.73 (100.09 to 123.28) | 1.03 (0.92 to 1.14) | -1.46 (-1.87 to -1.04) |
| Deaths | Armenia | 2.81 (2.36 to 3.23) | 0.31 (0.26 to 0.36) | 7.74 (6.22 to 9.97) | 1.35 (1.08 to 1.75) | 4.81 (2.47 to 7.21) |
| Deaths | Australia | 7.52 (6.74 to 8.31) | 0.18 (0.16 to 0.20) | 8.07 (7.09 to 9.03) | 0.16 (0.14 to 0.18) | -0.23 (-1.44 to 0.99) |
| Deaths | Austria | 4.01 (3.60 to 4.49) | 0.21 (0.18 to 0.23) | 3.21 (2.79 to 3.85) | 0.19 (0.17 to 0.23) | -0.12 (-1.14 to 0.91) |
| Deaths | Azerbaijan | 51.09 (36.87 to 68.00) | 2.37 (1.71 to 3.16) | 57.70 (40.89 to 79.33) | 2.41 (1.70 to 3.31) | 0.00 (-0.79 to 0.80) |
| Deaths | Bahamas | 2.21 (1.92 to 2.56) | 2.78 (2.41 to 3.21) | 3.90 (2.97 to 5.06) | 4.06 (3.09 to 5.26) | 1.18 (-0.05 to 2.43) |
| Deaths | Bahrain | 2.06 (1.40 to 3.01) | 1.39 (0.94 to 2.03) | 6.67 (4.87 to 9.43) | 1.66 (1.21 to 2.33) | 0.47 (-0.46 to 1.40) |
| Deaths | Bangladesh | 425.78 (297.95 to 602.78) | 1.47 (1.03 to 2.07) | 448.95 (302.89 to 689.36) | 1.02 (0.69 to 1.56) | -1.01 (-1.89 to -0.12) |
| Deaths | Barbados | 1.40 (1.25 to 1.56) | 2.04 (1.82 to 2.27) | 1.33 (1.00 to 1.75) | 2.26 (1.69 to 2.96) | 0.46 (-0.63 to 1.57) |
| Deaths | Belarus | 5.76 (5.14 to 6.44) | 0.24 (0.22 to 0.27) | 4.02 (3.24 to 4.84) | 0.27 (0.22 to 0.32) | -0.04 (-0.83 to 0.76) |
| Deaths | Belgium | 4.22 (3.79 to 4.73) | 0.19 (0.17 to 0.21) | 3.36 (2.97 to 3.98) | 0.16 (0.14 to 0.19) | -0.57 (-1.72 to 0.58) |
| Deaths | Belize | 1.27 (1.12 to 1.47) | 2.51 (2.21 to 2.91) | 5.89 (4.95 to 7.09) | 4.81 (4.04 to 5.78) | 2.11 (1.33 to 2.91) |
| Deaths | Benin | 52.88 (36.96 to 73.21) | 4.48 (3.14 to 6.20) | 165.69 (109.60 to 242.99) | 4.55 (3.01 to 6.67) | 0.06 (-0.22 to 0.35) |
| Deaths | Bermuda | 0.18 (0.15 to 0.20) | 1.13 (0.98 to 1.28) | 0.10 (0.08 to 0.13) | 1.09 (0.89 to 1.37) | -0.25 (-1.54 to 1.06) |
| Deaths | Bhutan | 2.95 (1.58 to 4.74) | 1.55 (0.83 to 2.48) | 3.39 (2.04 to 5.31) | 1.55 (0.93 to 2.43) | -0.02 (-0.27 to 0.22) |
| Deaths | Bolivia (Plurinational State of) | 59.65 (41.30 to 89.10) | 3.59 (2.48 to 5.36) | 90.13 (55.90 to 138.90) | 2.84 (1.76 to 4.38) | -0.75 (-0.93 to -0.57) |
| Deaths | Bosnia and Herzegovina | 10.78 (8.54 to 13.46) | 0.90 (0.72 to 1.13) | 4.04 (2.96 to 5.57) | 0.69 (0.50 to 0.95) | -0.94 (-1.76 to -0.11) |
| Deaths | Botswana | 5.34 (3.07 to 10.09) | 1.50 (0.86 to 2.88) | 9.68 (5.73 to 18.08) | 1.49 (0.88 to 2.78) | -0.05 (-0.83 to 0.73) |
| Deaths | Brazil | 670.79 (639.31 to 709.22) | 1.61 (1.54 to 1.70) | 498.84 (465.55 to 525.52) | 0.97 (0.90 to 1.02) | -1.53 (-1.81 to -1.25) |
| Deaths | Brunei Darussalam | 1.47 (1.01 to 2.01) | 1.85 (1.26 to 2.53) | 1.22 (0.85 to 1.64) | 0.99 (0.69 to 1.34) | -1.90 (-2.45 to -1.34) |
| Deaths | Bulgaria | 19.60 (17.52 to 21.56) | 1.11 (0.99 to 1.22) | 13.94 (10.80 to 18.53) | 1.41 (1.09 to 1.86) | 0.69 (-0.11 to 1.50) |
| Deaths | Burkina Faso | 100.53 (68.64 to 144.34) | 4.50 (3.09 to 6.46) | 300.32 (202.14 to 436.83) | 5.10 (3.44 to 7.41) | 0.45 (0.13 to 0.76) |
| Deaths | Burundi | 78.78 (52.02 to 114.64) | 5.51 (3.64 to 8.02) | 147.83 (96.22 to 235.41) | 4.18 (2.72 to 6.67) | -0.95 (-1.39 to -0.51) |
| Deaths | Cabo Verde | 2.48 (1.71 to 3.44) | 2.64 (1.82 to 3.65) | 3.72 (2.35 to 5.43) | 2.45 (1.54 to 3.56) | -0.26 (-0.54 to 0.03) |
| Deaths | Cambodia | 126.47 (79.58 to 176.59) | 4.72 (2.97 to 6.59) | 169.83 (102.26 to 267.79) | 3.74 (2.25 to 5.90) | -0.73 (-0.89 to -0.56) |
| Deaths | Cameroon | 168.27 (111.90 to 241.06) | 6.39 (4.25 to 9.13) | 562.05 (345.48 to 861.56) | 6.49 (4.00 to 9.93) | 0.05 (-0.07 to 0.17) |
| Deaths | Canada | 9.32 (8.44 to 10.45) | 0.14 (0.13 to 0.15) | 22.82 (20.12 to 25.57) | 0.32 (0.28 to 0.36) | 2.75 (1.82 to 3.69) |
| Deaths | Central African Republic | 45.68 (31.45 to 63.84) | 6.19 (4.27 to 8.65) | 97.30 (61.94 to 156.59) | 6.40 (4.07 to 10.30) | -0.02 (-0.42 to 0.38) |
| Deaths | Chad | 48.52 (31.70 to 78.62) | 3.32 (2.17 to 5.39) | 174.60 (111.90 to 278.49) | 3.90 (2.51 to 6.23) | 0.62 (0.18 to 1.06) |
| Deaths | Chile | 36.34 (32.74 to 40.21) | 0.96 (0.87 to 1.06) | 25.43 (22.04 to 28.33) | 0.58 (0.51 to 0.65) | -1.53 (-2.52 to -0.54) |
| Deaths | China | 6,681.93 (5,566.18 to 7,802.06) | 1.82 (1.51 to 2.12) | 2,219.98 (1,699.63 to 2,767.87) | 0.92 (0.71 to 1.15) | -2.17 (-2.49 to -1.85) |
| Deaths | Colombia | 220.50 (199.85 to 243.95) | 2.32 (2.10 to 2.56) | 131.19 (109.27 to 153.95) | 1.02 (0.85 to 1.20) | -2.68 (-3.59 to -1.77) |
| Deaths | Comoros | 5.80 (2.12 to 9.01) | 4.68 (1.76 to 7.24) | 9.76 (6.63 to 14.18) | 4.78 (3.25 to 6.94) | -0.16 (-3.86 to 3.68) |
| Deaths | Congo | 43.36 (27.19 to 61.52) | 6.41 (4.02 to 9.07) | 92.64 (60.00 to 134.93) | 6.45 (4.17 to 9.41) | 0.26 (-0.34 to 0.87) |
| Deaths | Cook Islands | 0.06 (0.04 to 0.09) | 1.18 (0.78 to 1.76) | 0.06 (0.04 to 0.08) | 1.48 (0.99 to 2.17) | 0.75 (0.48 to 1.02) |
| Deaths | Costa Rica | 8.78 (7.81 to 9.86) | 1.02 (0.91 to 1.15) | 20.97 (18.30 to 23.94) | 1.80 (1.57 to 2.06) | 1.77 (0.93 to 2.62) |
| Deaths | Coted'Ivoire | 155.80 (99.48 to 226.38) | 4.71 (3.01 to 6.84) | 371.17 (232.58 to 544.36) | 5.05 (3.17 to 7.41) | 0.23 (-0.27 to 0.73) |
| Deaths | Croatia | 8.47 (7.61 to 9.32) | 0.79 (0.71 to 0.88) | 3.45 (2.71 to 4.71) | 0.48 (0.38 to 0.65) | -1.66 (-2.20 to -1.12) |
| Deaths | Cuba | 38.78 (34.85 to 42.92) | 1.14 (1.02 to 1.26) | 22.02 (18.42 to 25.68) | 1.05 (0.88 to 1.22) | -0.21 (-1.03 to 0.61) |
| Deaths | Cyprus | 0.60 (0.43 to 0.89) | 0.31 (0.22 to 0.46) | 0.53 (0.39 to 0.76) | 0.19 (0.14 to 0.27) | -1.57 (-2.07 to -1.06) |
| Deaths | Czechia | 13.99 (12.72 to 15.41) | 0.65 (0.59 to 0.71) | 5.53 (4.50 to 7.24) | 0.36 (0.29 to 0.47) | -2.03 (-2.79 to -1.26) |
| Deaths | Democratic People's Republic of Korea | 118.62 (74.84 to 184.10) | 2.11 (1.33 to 3.27) | 114.36 (76.54 to 172.38) | 1.86 (1.25 to 2.80) | -0.40 (-0.46 to -0.33) |
| Deaths | Democratic Republic of the Congo | 500.41 (337.11 to 703.40) | 4.93 (3.32 to 6.93) | 1,227.21 (764.88 to 1,815.88) | 4.91 (3.07 to 7.26) | -0.04 (-0.39 to 0.31) |
| Deaths | Denmark | 1.45 (1.30 to 1.64) | 0.12 (0.11 to 0.14) | 1.11 (0.90 to 1.29) | 0.10 (0.08 to 0.11) | -0.43 (-1.53 to 0.68) |
| Deaths | Djibouti | 3.53 (2.21 to 5.35) | 2.81 (1.76 to 4.27) | 13.05 (7.88 to 20.71) | 3.91 (2.36 to 6.21) | 1.16 (0.49 to 1.83) |
| Deaths | Dominica | 0.43 (0.34 to 0.55) | 2.15 (1.67 to 2.73) | 0.67 (0.47 to 0.93) | 4.21 (2.92 to 5.81) | 2.17 (2.03 to 2.31) |
| Deaths | Dominican Republic | 44.54 (34.62 to 56.86) | 2.09 (1.63 to 2.67) | 90.36 (58.99 to 122.04) | 3.11 (2.03 to 4.20) | 1.23 (0.43 to 2.03) |
| Deaths | Ecuador | 75.63 (67.99 to 85.30) | 2.68 (2.41 to 3.02) | 97.93 (75.78 to 126.26) | 2.07 (1.60 to 2.67) | -0.86 (-2.55 to 0.86) |
| Deaths | Egypt | 347.27 (238.67 to 522.28) | 2.35 (1.62 to 3.53) | 762.44 (566.72 to 988.80) | 2.85 (2.12 to 3.70) | 0.69 (-0.02 to 1.41) |
| Deaths | El Salvador | 48.28 (37.53 to 64.84) | 3.33 (2.59 to 4.47) | 111.21 (78.79 to 151.19) | 6.32 (4.48 to 8.60) | 1.86 (0.35 to 3.39) |
| Deaths | Equatorial Guinea | 6.05 (3.95 to 8.71) | 5.65 (3.71 to 8.11) | 29.35 (15.03 to 50.42) | 6.06 (3.10 to 10.40) | 0.26 (-0.49 to 1.02) |
| Deaths | Eritrea | 41.54 (25.34 to 66.58) | 4.46 (2.72 to 7.17) | 84.43 (49.47 to 140.14) | 4.49 (2.63 to 7.46) | -0.03 (-0.36 to 0.30) |
| Deaths | Estonia | 6.78 (5.99 to 7.55) | 2.01 (1.78 to 2.24) | 3.41 (2.73 to 4.58) | 1.67 (1.34 to 2.23) | -1.25 (-3.29 to 0.83) |
| Deaths | Eswatini | 4.75 (3.20 to 6.99) | 2.24 (1.51 to 3.30) | 12.63 (6.69 to 19.82) | 3.77 (2.00 to 5.92) | 1.68 (1.38 to 1.98) |
| Deaths | Ethiopia | 1,061.90 (760.68 to 1,330.32) | 8.22 (5.91 to 10.28) | 1,375.49 (1,057.42 to 1,746.16) | 4.22 (3.24 to 5.36) | -2.14 (-2.27 to -2.01) |
| Deaths | Fiji | 6.59 (4.16 to 9.92) | 3.08 (1.94 to 4.63) | 10.55 (7.06 to 14.87) | 4.76 (3.18 to 6.71) | 1.44 (1.26 to 1.62) |
| Deaths | Finland | 0.85 (0.75 to 0.96) | 0.08 (0.07 to 0.09) | 0.80 (0.65 to 0.92) | 0.08 (0.07 to 0.10) | 0.25 (-1.04 to 1.56) |
| Deaths | France | 23.42 (21.07 to 25.93) | 0.18 (0.16 to 0.19) | 14.97 (13.34 to 16.72) | 0.13 (0.11 to 0.14) | -0.89 (-1.42 to -0.36) |
| Deaths | Gabon | 14.25 (9.74 to 19.80) | 5.34 (3.65 to 7.42) | 34.01 (17.27 to 54.17) | 6.74 (3.41 to 10.74) | 0.79 (0.59 to 0.99) |
| Deaths | Gambia | 11.60 (7.38 to 17.08) | 4.36 (2.78 to 6.42) | 41.07 (26.33 to 61.00) | 5.95 (3.83 to 8.84) | 0.99 (-0.96 to 2.97) |
| Deaths | Georgia | 17.92 (13.82 to 21.76) | 1.33 (1.03 to 1.62) | 16.44 (13.43 to 20.21) | 2.62 (2.14 to 3.22) | 2.22 (0.29 to 4.18) |
| Deaths | Germany | 44.37 (40.07 to 48.84) | 0.24 (0.21 to 0.26) | 21.69 (19.37 to 25.31) | 0.15 (0.14 to 0.18) | -1.33 (-2.04 to -0.61) |
| Deaths | Ghana | 185.84 (122.12 to 284.52) | 4.73 (3.11 to 7.22) | 620.93 (407.02 to 920.11) | 6.55 (4.30 to 9.70) | 1.08 (0.87 to 1.29) |
| Deaths | Greece | 7.63 (6.77 to 8.51) | 0.33 (0.29 to 0.36) | 5.01 (4.45 to 5.82) | 0.32 (0.29 to 0.37) | 0.13 (-0.45 to 0.71) |
| Deaths | Greenland | 0.05 (0.03 to 0.08) | 0.26 (0.16 to 0.45) | 0.03 (0.02 to 0.05) | 0.27 (0.17 to 0.42) | 0.15 (-0.19 to 0.48) |
| Deaths | Grenada | 0.97 (0.84 to 1.10) | 4.40 (3.81 to 5.01) | 1.25 (1.03 to 1.50) | 4.59 (3.78 to 5.53) | 0.01 (-1.50 to 1.53) |
| Deaths | Guam | 0.67 (0.52 to 0.94) | 1.61 (1.24 to 2.24) | 0.82 (0.62 to 1.07) | 2.30 (1.72 to 3.00) | 0.98 (0.04 to 1.93) |
| Deaths | Guatemala | 72.45 (65.44 to 80.77) | 3.60 (3.26 to 4.00) | 235.51 (196.91 to 277.07) | 5.08 (4.25 to 5.98) | 1.33 (-0.87 to 3.58) |
| Deaths | Guinea | 55.23 (37.41 to 82.76) | 4.03 (2.73 to 6.04) | 156.50 (101.96 to 240.05) | 4.46 (2.90 to 6.82) | 0.30 (0.08 to 0.53) |
| Deaths | Guinea-Bissau | 20.56 (13.69 to 28.96) | 7.93 (5.31 to 11.15) | 38.87 (25.74 to 56.40) | 6.84 (4.53 to 9.94) | -0.49 (-0.61 to -0.37) |
| Deaths | Guyana | 6.76 (5.31 to 8.11) | 2.91 (2.29 to 3.49) | 10.67 (7.21 to 14.58) | 4.95 (3.35 to 6.75) | 2.02 (1.17 to 2.88) |
| Deaths | Haiti | 57.64 (36.26 to 96.08) | 3.57 (2.25 to 5.96) | 116.25 (58.23 to 263.52) | 3.35 (1.68 to 7.59) | 0.07 (-0.63 to 0.77) |
| Deaths | Honduras | 16.32 (11.79 to 21.96) | 1.41 (1.02 to 1.89) | 30.86 (16.34 to 51.18) | 1.04 (0.55 to 1.72) | -1.03 (-1.53 to -0.54) |
| Deaths | Hungary | 14.15 (12.55 to 15.70) | 0.70 (0.62 to 0.77) | 5.66 (4.70 to 6.90) | 0.36 (0.30 to 0.44) | -2.38 (-2.77 to -1.99) |
| Deaths | Iceland | 0.06 (0.05 to 0.06) | 0.09 (0.08 to 0.10) | 0.08 (0.07 to 0.09) | 0.11 (0.09 to 0.12) | 0.49 (-0.55 to 1.55) |
| Deaths | India | 2,824.71 (2,401.78 to 3,592.18) | 1.25 (1.06 to 1.59) | 3,970.00 (3,204.02 to 5,121.66) | 1.02 (0.83 to 1.32) | -0.67 (-1.18 to -0.15) |
| Deaths | Indonesia | 2,101.88 (1,399.68 to 2,613.43) | 4.01 (2.67 to 4.97) | 2,841.27 (1,989.14 to 3,823.15) | 4.05 (2.83 to 5.44) | 0.04 (-0.10 to 0.17) |
| Deaths | Iran (Islamic Republic of) | 148.62 (118.65 to 209.06) | 0.98 (0.78 to 1.37) | 193.90 (164.79 to 221.41) | 1.10 (0.93 to 1.25) | 0.34 (0.05 to 0.63) |
| Deaths | Iraq | 124.92 (87.09 to 175.35) | 2.49 (1.74 to 3.49) | 177.73 (89.71 to 275.17) | 1.54 (0.78 to 2.38) | -1.60 (-1.96 to -1.24) |
| Deaths | Ireland | 1.23 (1.08 to 1.37) | 0.14 (0.13 to 0.16) | 1.07 (0.91 to 1.30) | 0.12 (0.10 to 0.15) | -0.31 (-0.92 to 0.30) |
| Deaths | Israel | 4.22 (3.76 to 4.72) | 0.36 (0.32 to 0.40) | 6.62 (5.88 to 7.71) | 0.32 (0.29 to 0.37) | -0.20 (-1.42 to 1.04) |
| Deaths | Italy | 29.95 (28.87 to 30.98) | 0.22 (0.21 to 0.23) | 11.44 (10.05 to 14.30) | 0.13 (0.11 to 0.16) | -1.90 (-2.94 to -0.85) |
| Deaths | Jamaica | 10.87 (9.67 to 12.52) | 1.59 (1.42 to 1.83) | 17.65 (11.85 to 24.18) | 2.26 (1.52 to 3.09) | 1.09 (-0.49 to 2.70) |
| Deaths | Japan | 91.06 (88.04 to 93.91) | 0.34 (0.32 to 0.35) | 30.08 (28.84 to 31.24) | 0.16 (0.15 to 0.17) | -2.27 (-2.88 to -1.64) |
| Deaths | Jordan | 19.71 (14.30 to 27.28) | 1.73 (1.25 to 2.40) | 47.99 (35.13 to 62.61) | 1.37 (1.00 to 1.79) | -0.71 (-1.42 to 0.00) |
| Deaths | Kazakhstan | 92.88 (83.38 to 106.92) | 2.16 (1.94 to 2.49) | 58.66 (45.50 to 78.28) | 1.48 (1.15 to 1.97) | -1.32 (-2.10 to -0.54) |
| Deaths | Kenya | 171.46 (128.33 to 254.26) | 2.69 (2.01 to 4.00) | 526.53 (398.88 to 724.13) | 3.55 (2.69 to 4.89) | 0.90 (0.75 to 1.06) |
| Deaths | Kiribati | 0.81 (0.59 to 1.09) | 3.87 (2.81 to 5.20) | 1.74 (1.01 to 2.88) | 5.40 (3.14 to 8.95) | 1.09 (1.00 to 1.19) |
| Deaths | Kuwait | 8.77 (7.59 to 10.18) | 1.72 (1.49 to 1.99) | 7.55 (5.98 to 9.20) | 0.74 (0.59 to 0.91) | -2.35 (-3.30 to -1.40) |
| Deaths | Kyrgyzstan | 31.88 (27.50 to 37.39) | 2.65 (2.29 to 3.11) | 45.37 (36.96 to 54.59) | 2.63 (2.14 to 3.17) | -0.22 (-1.20 to 0.78) |
| Deaths | Lao People's Democratic Republic | 63.01 (41.85 to 90.93) | 6.02 (4.00 to 8.70) | 104.92 (65.77 to 166.41) | 5.09 (3.19 to 8.07) | -0.54 (-0.73 to -0.34) |
| Deaths | Latvia | 5.18 (4.65 to 5.74) | 0.89 (0.80 to 0.99) | 2.12 (1.72 to 2.75) | 0.76 (0.61 to 0.98) | -0.67 (-1.81 to 0.48) |
| Deaths | Lebanon | 14.48 (9.60 to 22.15) | 1.84 (1.22 to 2.82) | 17.10 (12.51 to 23.23) | 1.29 (0.94 to 1.75) | -1.11 (-1.40 to -0.82) |
| Deaths | Lesotho | 3.63 (2.35 to 5.53) | 1.00 (0.64 to 1.53) | 15.91 (9.96 to 23.98) | 2.86 (1.79 to 4.31) | 3.53 (3.20 to 3.87) |
| Deaths | Liberia | 40.78 (24.21 to 74.66) | 6.50 (3.86 to 11.95) | 97.61 (63.91 to 142.76) | 6.56 (4.30 to 9.59) | 0.11 (-0.44 to 0.66) |
| Deaths | Libya | 20.44 (13.98 to 29.62) | 1.75 (1.20 to 2.53) | 44.56 (25.13 to 65.35) | 2.52 (1.42 to 3.70) | 1.23 (0.49 to 1.99) |
| Deaths | Lithuania | 6.41 (5.76 to 7.28) | 0.73 (0.66 to 0.83) | 3.22 (2.51 to 4.56) | 0.66 (0.52 to 0.93) | -0.42 (-1.51 to 0.69) |
| Deaths | Luxembourg | 0.21 (0.18 to 0.24) | 0.24 (0.21 to 0.27) | 0.17 (0.14 to 0.20) | 0.14 (0.12 to 0.17) | -1.53 (-3.13 to 0.09) |
| Deaths | Madagascar | 119.23 (84.19 to 166.84) | 3.75 (2.65 to 5.25) | 305.12 (201.78 to 439.33) | 3.74 (2.48 to 5.37) | 0.02 (-0.34 to 0.39) |
| Deaths | Malawi | 136.74 (92.36 to 191.84) | 5.06 (3.42 to 7.09) | 347.88 (233.01 to 496.65) | 6.02 (4.04 to 8.56) | 0.61 (0.37 to 0.86) |
| Deaths | Malaysia | 104.31 (77.48 to 137.60) | 2.14 (1.59 to 2.82) | 157.73 (116.82 to 206.89) | 1.82 (1.35 to 2.39) | -0.81 (-1.26 to -0.35) |
| Deaths | Maldives | 2.70 (1.95 to 3.55) | 4.65 (3.34 to 6.09) | 2.92 (2.01 to 3.95) | 2.20 (1.53 to 2.97) | -2.25 (-2.92 to -1.57) |
| Deaths | Mali | 95.07 (66.44 to 133.27) | 4.67 (3.27 to 6.55) | 270.46 (182.94 to 392.19) | 4.31 (2.91 to 6.25) | -0.25 (-0.45 to -0.05) |
| Deaths | Malta | 0.20 (0.18 to 0.23) | 0.25 (0.22 to 0.29) | 0.14 (0.12 to 0.17) | 0.21 (0.17 to 0.25) | 0.16 (-0.53 to 0.85) |
| Deaths | Marshall Islands | 0.44 (0.26 to 0.76) | 3.96 (2.35 to 6.69) | 0.98 (0.29 to 2.97) | 6.65 (1.94 to 20.00) | 1.72 (1.52 to 1.92) |
| Deaths | Mauritania | 25.88 (17.53 to 35.78) | 4.91 (3.33 to 6.78) | 43.22 (26.95 to 65.80) | 3.69 (2.30 to 5.61) | -1.00 (-1.18 to -0.81) |
| Deaths | Mauritius | 11.80 (10.56 to 13.26) | 3.66 (3.27 to 4.11) | 22.50 (19.23 to 25.51) | 7.86 (6.71 to 8.92) | 2.35 (0.70 to 4.03) |
| Deaths | Mexico | 790.99 (764.57 to 828.79) | 3.22 (3.11 to 3.37) | 1,584.58 (1,413.75 to 1,806.39) | 4.89 (4.36 to 5.57) | 1.39 (1.07 to 1.70) |
| Deaths | Micronesia (Federated States of) | 0.93 (0.57 to 1.52) | 3.62 (2.21 to 5.84) | 1.73 (1.10 to 2.67) | 6.16 (3.91 to 9.54) | 1.76 (1.65 to 1.87) |
| Deaths | Monaco | 0.01 (0.01 to 0.01) | 0.18 (0.12 to 0.25) | 0.01 (0.01 to 0.02) | 0.26 (0.17 to 0.38) | 1.27 (1.17 to 1.37) |
| Deaths | Mongolia | 16.42 (11.90 to 24.74) | 2.65 (1.92 to 3.98) | 19.47 (14.59 to 26.43) | 2.69 (2.02 to 3.65) | 0.04 (-0.54 to 0.62) |
| Deaths | Montenegro | 1.92 (1.44 to 2.50) | 1.22 (0.92 to 1.59) | 1.30 (0.97 to 1.72) | 1.07 (0.79 to 1.41) | -0.14 (-2.17 to 1.93) |
| Deaths | Morocco | 111.72 (70.57 to 172.84) | 1.57 (0.99 to 2.43) | 133.50 (86.20 to 221.75) | 1.47 (0.95 to 2.45) | -0.25 (-0.53 to 0.03) |
| Deaths | Mozambique | 116.90 (75.15 to 188.33) | 3.54 (2.28 to 5.71) | 499.23 (317.38 to 763.61) | 5.74 (3.64 to 8.77) | 1.59 (1.40 to 1.77) |
| Deaths | Myanmar | 841.04 (510.19 to 1,209.26) | 7.21 (4.38 to 10.35) | 697.81 (466.44 to 982.96) | 4.83 (3.23 to 6.81) | -1.26 (-1.43 to -1.10) |
| Deaths | Namibia | 4.51 (2.87 to 8.47) | 1.15 (0.73 to 2.18) | 8.54 (4.81 to 15.24) | 1.24 (0.70 to 2.21) | 0.34 (-0.01 to 0.70) |
| Deaths | Nauru | 0.11 (0.07 to 0.17) | 4.16 (2.50 to 6.44) | 0.21 (0.13 to 0.30) | 6.84 (4.46 to 9.90) | 1.61 (1.49 to 1.74) |
| Deaths | Nepal | 70.59 (47.09 to 103.74) | 1.43 (0.95 to 2.10) | 143.64 (88.22 to 222.65) | 1.59 (0.98 to 2.46) | 0.36 (0.13 to 0.58) |
| Deaths | Netherlands | 5.03 (4.45 to 5.70) | 0.13 (0.12 to 0.15) | 4.46 (3.89 to 5.16) | 0.14 (0.12 to 0.16) | 0.28 (-0.37 to 0.92) |
| Deaths | New Zealand | 2.38 (2.16 to 2.60) | 0.28 (0.25 to 0.30) | 3.20 (2.86 to 3.52) | 0.29 (0.26 to 0.32) | 0.08 (-1.46 to 1.65) |
| Deaths | Nicaragua | 29.24 (22.84 to 39.95) | 2.84 (2.22 to 3.89) | 79.32 (58.80 to 104.32) | 4.33 (3.21 to 5.69) | 1.19 (0.88 to 1.50) |
| Deaths | Niger | 70.31 (45.64 to 112.43) | 3.62 (2.36 to 5.80) | 187.13 (111.85 to 336.07) | 2.93 (1.74 to 5.29) | -0.67 (-0.85 to -0.48) |
| Deaths | Nigeria | 784.29 (538.67 to 1,076.79) | 3.31 (2.27 to 4.55) | 1,922.65 (1,115.36 to 3,025.40) | 3.08 (1.79 to 4.86) | -0.26 (-0.42 to -0.09) |
| Deaths | Niue | 0.01 (0.01 to 0.02) | 2.69 (1.75 to 4.09) | 0.03 (0.02 to 0.05) | 8.11 (4.42 to 13.71) | 3.85 (3.45 to 4.25) |
| Deaths | North Macedonia | 4.24 (3.28 to 5.51) | 0.85 (0.66 to 1.11) | 2.73 (1.86 to 4.55) | 0.64 (0.44 to 1.06) | -1.01 (-1.99 to -0.01) |
| Deaths | Northern Mariana Islands | 0.39 (0.25 to 0.58) | 2.62 (1.69 to 3.92) | 0.34 (0.24 to 0.45) | 3.46 (2.48 to 4.64) | 1.19 (0.84 to 1.54) |
| Deaths | Norway | 0.78 (0.73 to 0.83) | 0.08 (0.07 to 0.08) | 0.97 (0.88 to 1.06) | 0.09 (0.08 to 0.10) | 0.64 (-0.69 to 1.98) |
| Deaths | Oman | 5.47 (3.48 to 8.96) | 1.08 (0.69 to 1.75) | 15.89 (10.59 to 22.26) | 1.38 (0.94 to 1.96) | 0.87 (-0.02 to 1.76) |
| Deaths | Pakistan | 515.94 (370.97 to 693.65) | 1.83 (1.32 to 2.46) | 1,882.38 (1,330.55 to 2,584.61) | 2.87 (2.03 to 3.94) | 1.47 (1.34 to 1.59) |
| Deaths | Palau | 0.14 (0.08 to 0.20) | 3.01 (1.85 to 4.51) | 0.26 (0.13 to 0.39) | 7.55 (3.74 to 11.56) | 3.03 (2.79 to 3.27) |
| Deaths | Palestine | 11.43 (7.31 to 17.57) | 2.04 (1.30 to 3.14) | 25.71 (19.16 to 34.34) | 1.71 (1.28 to 2.29) | -0.54 (-0.93 to -0.15) |
| Deaths | Panama | 10.07 (8.99 to 11.40) | 1.47 (1.31 to 1.66) | 23.15 (18.43 to 28.07) | 2.18 (1.74 to 2.65) | 1.27 (0.45 to 2.09) |
| Deaths | Papua New Guinea | 21.66 (7.82 to 35.12) | 1.91 (0.70 to 3.09) | 69.35 (43.41 to 99.97) | 2.45 (1.54 to 3.54) | 0.89 (0.62 to 1.16) |
| Deaths | Paraguay | 11.84 (9.20 to 15.43) | 1.14 (0.88 to 1.48) | 25.93 (18.83 to 35.79) | 1.34 (0.97 to 1.84) | 0.57 (-0.56 to 1.70) |
| Deaths | Peru | 163.39 (125.88 to 206.04) | 2.68 (2.07 to 3.38) | 196.75 (133.23 to 273.52) | 2.09 (1.42 to 2.90) | -0.83 (-2.09 to 0.45) |
| Deaths | Philippines | 766.64 (672.57 to 865.53) | 4.39 (3.85 to 4.96) | 1,582.06 (1,249.79 to 1,847.95) | 5.12 (4.05 to 5.98) | 0.48 (0.27 to 0.70) |
| Deaths | Poland | 83.20 (80.72 to 86.09) | 1.05 (1.02 to 1.09) | 17.55 (15.32 to 20.62) | 0.28 (0.24 to 0.32) | -4.32 (-4.84 to -3.79) |
| Deaths | Portugal | 11.16 (10.01 to 12.43) | 0.47 (0.42 to 0.52) | 4.12 (3.66 to 4.76) | 0.24 (0.21 to 0.28) | -1.95 (-3.31 to -0.57) |
| Deaths | Puerto Rico | 22.21 (20.03 to 24.59) | 2.48 (2.24 to 2.74) | 17.04 (13.91 to 20.52) | 2.67 (2.18 to 3.22) | 0.29 (-0.87 to 1.46) |
| Deaths | Qatar | 1.47 (0.76 to 2.60) | 1.22 (0.63 to 2.14) | 7.12 (4.93 to 10.58) | 1.02 (0.71 to 1.50) | -0.72 (-1.58 to 0.14) |
| Deaths | Republic of Korea | 130.67 (93.45 to 161.01) | 0.96 (0.69 to 1.19) | 19.15 (14.86 to 27.73) | 0.20 (0.16 to 0.29) | -4.78 (-5.71 to -3.85) |
| Deaths | Republic of Moldova | 5.19 (4.67 to 5.89) | 0.51 (0.46 to 0.58) | 3.22 (2.72 to 4.03) | 0.51 (0.43 to 0.65) | -0.24 (-1.33 to 0.86) |
| Deaths | Romania | 60.74 (54.86 to 67.83) | 1.20 (1.09 to 1.34) | 15.02 (12.17 to 19.86) | 0.51 (0.41 to 0.67) | -2.72 (-3.65 to -1.79) |
| Deaths | Russian Federation | 596.66 (581.36 to 616.50) | 1.77 (1.73 to 1.83) | 113.93 (101.55 to 134.57) | 0.50 (0.44 to 0.59) | -4.15 (-4.76 to -3.55) |
| Deaths | Rwanda | 130.50 (93.60 to 179.20) | 6.94 (4.99 to 9.51) | 154.37 (100.83 to 233.02) | 4.08 (2.67 to 6.17) | -1.66 (-2.06 to -1.26) |
| Deaths | Saint Kitts and Nevis | 0.39 (0.33 to 0.45) | 3.47 (2.94 to 4.07) | 0.43 (0.33 to 0.60) | 3.24 (2.45 to 4.47) | -0.23 (-1.25 to 0.80) |
| Deaths | Saint Lucia | 1.05 (0.93 to 1.19) | 2.70 (2.38 to 3.04) | 1.56 (1.20 to 1.92) | 3.80 (2.94 to 4.69) | 1.05 (0.35 to 1.76) |
| Deaths | Saint Vincent and the Grenadines | 0.83 (0.73 to 0.95) | 2.61 (2.30 to 3.00) | 1.10 (0.91 to 1.35) | 4.24 (3.48 to 5.18) | 1.29 (-0.30 to 2.90) |
| Deaths | Samoa | 1.21 (0.75 to 1.94) | 2.58 (1.60 to 4.13) | 2.13 (1.35 to 3.15) | 4.05 (2.55 to 5.99) | 1.47 (1.32 to 1.62) |
| Deaths | San Marino | 0.01 (0.00 to 0.01) | 0.09 (0.06 to 0.12) | 0.00 (0.00 to 0.01) | 0.09 (0.06 to 0.13) | -0.15 (-0.50 to 0.21) |
| Deaths | Sao Tome and Principe | 1.28 (0.58 to 2.18) | 4.20 (1.94 to 7.07) | 4.03 (2.28 to 6.26) | 6.72 (3.77 to 10.49) | 1.39 (0.72 to 2.06) |
| Deaths | Saudi Arabia | 108.98 (69.90 to 163.94) | 2.44 (1.56 to 3.67) | 381.48 (249.77 to 542.44) | 3.73 (2.46 to 5.29) | 1.39 (1.11 to 1.67) |
| Deaths | Senegal | 107.64 (76.12 to 155.35) | 5.55 (3.92 to 8.03) | 228.86 (145.97 to 367.79) | 5.18 (3.31 to 8.34) | -0.01 (-0.22 to 0.20) |
| Deaths | Serbia | 27.57 (21.15 to 35.32) | 1.28 (0.98 to 1.64) | 10.65 (8.17 to 14.08) | 0.62 (0.48 to 0.82) | -2.31 (-2.86 to -1.75) |
| Deaths | Seychelles | 0.68 (0.53 to 0.88) | 3.22 (2.48 to 4.13) | 0.96 (0.72 to 1.23) | 4.10 (3.08 to 5.28) | 1.87 (1.57 to 2.17) |
| Deaths | Sierra Leone | 36.26 (21.24 to 57.54) | 3.33 (1.95 to 5.29) | 93.36 (58.89 to 148.64) | 3.64 (2.30 to 5.80) | 0.23 (-0.43 to 0.90) |
| Deaths | Singapore | 4.79 (4.26 to 5.41) | 0.50 (0.45 to 0.57) | 2.40 (2.07 to 2.78) | 0.27 (0.23 to 0.31) | -1.90 (-2.67 to -1.12) |
| Deaths | Slovakia | 9.80 (7.79 to 12.32) | 0.82 (0.65 to 1.03) | 5.66 (4.32 to 7.38) | 0.61 (0.47 to 0.80) | -0.93 (-1.62 to -0.24) |
| Deaths | Slovenia | 2.55 (2.29 to 2.88) | 0.56 (0.50 to 0.63) | 0.68 (0.53 to 0.96) | 0.23 (0.18 to 0.32) | -3.30 (-4.86 to -1.71) |
| Deaths | Solomon Islands | 2.97 (0.65 to 5.30) | 3.37 (0.75 to 6.01) | 7.56 (4.44 to 10.85) | 4.23 (2.48 to 6.07) | 0.75 (0.22 to 1.29) |
| Deaths | Somalia | 109.29 (65.62 to 184.71) | 5.65 (3.39 to 9.64) | 347.77 (209.11 to 620.67) | 5.83 (3.51 to 10.38) | 0.11 (0.02 to 0.19) |
| Deaths | South Africa | 246.28 (207.41 to 303.79) | 2.34 (1.97 to 2.89) | 282.37 (236.52 to 342.17) | 1.93 (1.62 to 2.34) | -0.64 (-1.48 to 0.20) |
| Deaths | South Sudan | 62.12 (40.22 to 91.10) | 3.70 (2.41 to 5.42) | 162.01 (101.18 to 254.59) | 6.44 (4.01 to 10.28) | 1.85 (0.95 to 2.76) |
| Deaths | Spain | 36.16 (32.30 to 40.00) | 0.38 (0.34 to 0.42) | 12.61 (11.09 to 14.65) | 0.18 (0.16 to 0.21) | -2.30 (-3.02 to -1.57) |
| Deaths | Sri Lanka | 154.17 (114.53 to 205.26) | 3.20 (2.38 to 4.26) | 117.16 (78.17 to 164.92) | 2.36 (1.57 to 3.32) | -1.03 (-2.93 to 0.90) |
| Deaths | Sudan | 90.60 (54.23 to 157.90) | 1.72 (1.03 to 3.00) | 245.96 (139.45 to 370.58) | 1.97 (1.11 to 2.96) | 0.43 (0.30 to 0.55) |
| Deaths | Suriname | 3.58 (2.36 to 4.64) | 3.13 (2.06 to 4.05) | 6.23 (4.53 to 8.31) | 4.68 (3.41 to 6.24) | 1.49 (0.69 to 2.31) |
| Deaths | Sweden | 0.86 (0.78 to 0.93) | 0.05 (0.04 to 0.05) | 1.60 (1.40 to 1.82) | 0.08 (0.07 to 0.10) | 1.89 (1.38 to 2.41) |
| Deaths | Switzerland | 3.37 (3.02 to 3.73) | 0.21 (0.18 to 0.23) | 2.01 (1.76 to 2.40) | 0.13 (0.11 to 0.15) | -1.58 (-2.30 to -0.85) |
| Deaths | Syrian Arab Republic | 128.70 (87.37 to 177.29) | 3.73 (2.54 to 5.12) | 100.23 (67.06 to 157.22) | 3.03 (1.95 to 5.34) | -0.70 (-1.20 to -0.20) |
| Deaths | Taiwan (Province of China) | 97.26 (88.45 to 106.72) | 1.69 (1.54 to 1.86) | 32.88 (27.59 to 37.34) | 0.76 (0.64 to 0.87) | -2.56 (-3.34 to -1.77) |
| Deaths | Tajikistan | 11.18 (8.65 to 14.69) | 0.76 (0.58 to 0.99) | 21.84 (14.39 to 34.25) | 0.82 (0.54 to 1.29) | 0.31 (-0.11 to 0.73) |
| Deaths | Thailand | 567.35 (392.37 to 831.89) | 3.29 (2.28 to 4.82) | 377.22 (257.94 to 535.55) | 2.97 (2.03 to 4.22) | -0.40 (-1.23 to 0.44) |
| Deaths | Timor-Leste | 6.66 (4.02 to 10.14) | 3.07 (1.86 to 4.68) | 12.92 (8.05 to 20.08) | 3.16 (1.96 to 4.91) | 0.10 (-1.31 to 1.53) |
| Deaths | Togo | 38.92 (26.90 to 54.52) | 4.03 (2.79 to 5.64) | 94.12 (60.40 to 139.82) | 4.23 (2.72 to 6.27) | 0.18 (-0.07 to 0.43) |
| Deaths | Tokelau | 0.01 (0.01 to 0.02) | 2.41 (1.42 to 4.15) | 0.02 (0.01 to 0.03) | 6.68 (4.59 to 9.52) | 3.68 (3.05 to 4.31) |
| Deaths | Tonga | 0.24 (0.14 to 0.40) | 0.94 (0.54 to 1.56) | 0.41 (0.25 to 0.64) | 1.61 (0.96 to 2.50) | 1.81 (1.49 to 2.14) |
| Deaths | Trinidad and Tobago | 7.31 (6.51 to 8.35) | 2.22 (1.98 to 2.53) | 10.50 (7.42 to 13.88) | 3.84 (2.72 to 5.08) | 1.96 (0.89 to 3.05) |
| Deaths | Tunisia | 25.37 (16.90 to 39.37) | 1.08 (0.72 to 1.67) | 31.90 (20.84 to 47.93) | 1.28 (0.84 to 1.93) | 0.56 (0.32 to 0.81) |
| Deaths | Turkey | 320.99 (221.24 to 455.93) | 1.98 (1.37 to 2.82) | 208.64 (150.68 to 281.09) | 1.08 (0.78 to 1.46) | -1.98 (-2.37 to -1.59) |
| Deaths | Turkmenistan | 34.78 (31.16 to 39.45) | 3.31 (2.97 to 3.76) | 86.08 (65.01 to 111.86) | 6.57 (4.96 to 8.53) | 2.40 (1.48 to 3.32) |
| Deaths | Tuvalu | 0.07 (0.05 to 0.11) | 3.08 (2.02 to 4.88) | 0.13 (0.09 to 0.21) | 4.14 (2.69 to 6.34) | 0.98 (0.85 to 1.11) |
| Deaths | Uganda | 132.48 (82.25 to 205.12) | 2.80 (1.74 to 4.34) | 540.38 (356.02 to 799.84) | 4.36 (2.87 to 6.47) | 1.33 (0.96 to 1.70) |
| Deaths | Ukraine | 6.30 (5.72 to 7.02) | 0.06 (0.05 to 0.06) | 55.73 (40.33 to 72.31) | 0.79 (0.57 to 1.02) | 8.88 (6.18 to 11.66) |
| Deaths | United Arab Emirates | 4.70 (2.90 to 7.55) | 0.96 (0.59 to 1.53) | 15.41 (7.24 to 22.39) | 1.58 (0.75 to 2.28) | 1.89 (0.21 to 3.59) |
| Deaths | United Kingdom | 21.68 (21.21 to 22.15) | 0.16 (0.16 to 0.17) | 21.74 (20.40 to 24.31) | 0.17 (0.16 to 0.19) | -0.10 (-1.87 to 1.71) |
| Deaths | United Republic of Tanzania | 294.35 (206.77 to 425.78) | 4.22 (2.97 to 6.10) | 705.08 (472.52 to 998.11) | 4.35 (2.92 to 6.16) | 0.11 (-0.11 to 0.32) |
| Deaths | United States Virgin Islands | 200.89 (195.24 to 207.19) | 0.32 (0.31 to 0.33) | 342.86 (299.92 to 370.49) | 0.50 (0.44 to 0.54) | 1.50 (0.89 to 2.12) |
| Deaths | United States of America | 0.49 (0.36 to 0.68) | 2.05 (1.49 to 2.83) | 0.61 (0.39 to 0.98) | 4.53 (2.90 to 7.32) | 2.91 (2.05 to 3.77) |
| Deaths | Uruguay | 4.19 (3.74 to 4.72) | 0.59 (0.52 to 0.66) | 5.48 (4.70 to 6.19) | 0.74 (0.63 to 0.83) | 0.62 (-0.06 to 1.30) |
| Deaths | Uzbekistan | 76.34 (61.91 to 98.41) | 1.30 (1.06 to 1.68) | 291.33 (241.14 to 346.30) | 3.48 (2.88 to 4.14) | 3.00 (2.21 to 3.80) |
| Deaths | Vanuatu | 0.84 (0.44 to 1.51) | 2.13 (1.13 to 3.82) | 3.13 (1.93 to 5.11) | 3.77 (2.32 to 6.14) | 1.91 (1.55 to 2.26) |
| Deaths | Venezuela (Bolivarian Republic of) | 95.06 (86.48 to 104.78) | 1.78 (1.62 to 1.96) | 187.48 (139.21 to 242.64) | 3.57 (2.65 to 4.61) | 2.30 (1.32 to 3.29) |
| Deaths | Viet Nam | 566.63 (292.85 to 808.23) | 2.92 (1.51 to 4.17) | 532.97 (280.32 to 751.26) | 2.48 (1.29 to 3.49) | -0.54 (-0.65 to -0.43) |
| Deaths | Yemen | 20.44 (6.95 to 50.23) | 0.68 (0.24 to 1.63) | 59.96 (26.99 to 140.03) | 0.68 (0.31 to 1.57) | -0.01 (-1.14 to 1.13) |
| Deaths | Zambia | 135.54 (92.96 to 184.86) | 6.08 (4.19 to 8.28) | 381.87 (213.17 to 713.68) | 6.79 (3.80 to 12.67) | 0.36 (0.19 to 0.53) |
| Deaths | Zimbabwe | 31.58 (20.30 to 57.66) | 1.15 (0.73 to 2.14) | 125.92 (79.78 to 203.15) | 2.99 (1.89 to 4.87) | 3.19 (2.28 to 4.10) |
