# Supplementary material for: Global, regional, and national burden of chronic kidney disease among adolescents and emerging adults from 1990 to 2021
Source: Ren Fail. 2025 May 22;47(1):2508296. doi: 10.1080/0886022X.2025.2508296 (PMC12101043; doi:10.1080/0886022X.2025.2508296)
Supplement: Supplementary Table S3.docx [file IRNF_A_2508296_SM3077.docx]

Supplementary Table S3 Global DALYs of CKD and Their AAPCs From 1990 to 2021 by Sex, Age, Cause, SDI, Region and Country in Adolescents and Emerging adults

| **Measure** | **Variable** | 1990 | | 2021 | | AAPC (95% UI) |
| --- | --- | --- | --- | --- | --- | --- |
|  |  | Number (95% UI) | ASR (95% UI) | Number (95% UI) | ASR (95% UI) |  |
| DALYs | Global | 2,303,296.57 (1,998,225.78 to 2,638,352.68) | 159.08 (138.04 to 182.27) | 2,994,742.73 (2,626,252.46 to 3,391,497.98) | 165.14 (144.82 to 187.02) | 0.10 (-0.04 to 0.24) |
|  | **Sex** | | | | | |
| DALYs | Male | 1,245,102.40 (1,026,977.78 to 1,448,314.13) | 170.27 (140.50 to 198.12) | 1,735,379.20 (1,484,249.68 to 2,004,742.11) | 188.23 (160.98 to 217.47) | 0.32 (0.28 to 0.36) |
| DALYs | Female | 1,058,194.17 (927,445.19 to 1,218,933.50) | 147.65 (129.44 to 170.12) | 1,259,363.53 (1,093,882.27 to 1,456,991.81) | 141.22 (122.68 to 163.37) | -0.17 (-0.38 to 0.04) |
| DALYs | **Age** | | | | | |
| DALYs | 15-19 | 668,428.10 (573,572.91 to 753,063.08) | 128.69 (110.42 to 144.98) | 797,195.98 (697,774.19 to 910,717.05) | 127.76 (111.83 to 145.95) | -0.03 (-0.11 to 0.06) |
| DALYs | 20-24 | 796,330.00 (691,716.96 to 915,786.40) | 161.83 (140.57 to 186.10) | 1,012,023.42 (886,500.05 to 1,132,616.07) | 169.47 (148.45 to 189.67) | 0.14 (0.03 to 0.24) |
| DALYs | 25-29 | 838,538.46 (732,935.92 to 969,503.20) | 189.45 (165.59 to 219.04) | 1,185,523.34 (1,041,978.23 to 1,348,164.87) | 201.50 (177.10 to 229.15) | 0.18 (0.03 to 0.33) |
|  | **Cause** | | | | | |
| DALYs | Diabetes mellitus type 1 | 322,677.40 (201,537.28 to 465,597.72) | 22.48 (14.08 to 32.37) | 287,436.32 (174,944.07 to 424,799.89) | 15.78 (9.60 to 23.33) | -1.18 (-1.46 to -0.91) |
| DALYs | Diabetes mellitus type 2 | 16,363.43 (9,471.36 to 27,857.78) | 1.16 (0.67 to 1.98) | 14,620.75 (8,217.06 to 25,485.19) | 0.80 (0.45 to 1.39) | -1.22 (-1.36 to -1.07) |
| DALYs | Hypertension | 399,428.15 (261,896.45 to 572,083.61) | 27.69 (18.18 to 39.59) | 469,476.44 (310,215.71 to 663,552.18) | 25.82 (17.06 to 36.51) | -0.25 (-0.34 to -0.15) |
| DALYs | Glomerulonephritis | 752,118.67 (552,973.17 to 989,582.69) | 51.79 (38.11 to 68.13) | 1,120,005.74 (830,918.49 to 1,464,986.87) | 61.84 (45.86 to 80.88) | 0.58 (0.46 to 0.70) |
| DALYs | Other and unspecified causes | 812,708.92 (595,383.56 to 1,071,872.72) | 55.96 (41.01 to 73.78) | 1,103,203.48 (816,160.82 to 1,446,617.59) | 60.90 (45.05 to 79.87) | 0.28 (0.17 to 0.38) |
|  | **SDI** | | | | | |
| DALYs | High SDI | 117,397.35 (97,987.69 to 142,068.93) | 55.37 (46.28 to 67.01) | 120,132.18 (95,871.36 to 148,357.90) | 58.53 (46.74 to 72.33) | 0.17 (0.05 to 0.29) |
| DALYs | High-middle SDI | 331,672.46 (288,765.99 to 386,074.14) | 115.09 (100.21 to 133.95) | 185,537.35 (157,807.38 to 220,935.13) | 78.00 (66.39 to 92.88) | -1.30 (-1.45 to -1.15) |
| DALYs | Middle SDI | 949,869.49 (810,200.47 to 1,084,338.05) | 185.60 (158.37 to 211.92) | 929,769.57 (812,887.64 to 1,048,603.43) | 169.39 (148.12 to 190.99) | -0.32 (-0.50 to -0.13) |
| DALYs | Low-middle SDI | 544,603.52 (469,178.82 to 638,287.45) | 177.02 (152.46 to 207.60) | 950,442.48 (829,353.64 to 1,090,013.55) | 182.99 (159.67 to 209.88) | 0.09 (-0.04 to 0.22) |
| DALYs | Low SDI | 357,728.87 (297,912.86 to 412,969.14) | 278.31 (232.20 to 321.95) | 806,197.59 (672,380.51 to 972,648.48) | 259.52 (216.72 to 312.96) | -0.24 (-0.36 to -0.11) |
|  | **Region** | | | | | |
| DALYs | Andean Latin America | 22,957.04 (19,667.41 to 26,848.77) | 216.50 (185.45 to 253.25) | 29,548.16 (24,007.77 to 36,159.34) | 170.96 (138.85 to 209.22) | -0.81 (-1.58 to -0.04) |
| DALYs | Australasia | 1,284.21 (999.59 to 1,658.48) | 25.63 (19.96 to 33.08) | 1,600.66 (1,195.20 to 2,156.17) | 25.59 (19.13 to 34.42) | 0.04 (-0.44 to 0.52) |
| DALYs | Caribbean | 16,767.37 (14,525.70 to 19,814.43) | 165.16 (143.10 to 195.14) | 24,619.12 (19,495.08 to 33,496.20) | 217.42 (172.08 to 296.10) | 0.94 (0.39 to 1.49) |
| DALYs | Central Asia | 32,661.42 (27,742.59 to 38,702.18) | 172.54 (146.55 to 204.47) | 52,323.40 (44,541.82 to 61,843.14) | 234.97 (199.87 to 277.86) | 0.88 (0.24 to 1.53) |
| DALYs | Central Europe | 25,753.71 (22,189.97 to 30,690.09) | 95.03 (81.91 to 113.15) | 10,504.16 (8,193.74 to 13,537.90) | 55.34 (43.04 to 71.59) | -1.77 (-2.06 to -1.47) |
| DALYs | Central Latin America | 101,941.08 (94,241.08 to 111,691.87) | 217.90 (201.18 to 238.90) | 181,141.43 (160,747.57 to 204,998.53) | 283.16 (251.27 to 320.44) | 0.82 (0.64 to 1.00) |
| DALYs | Central Sub-Saharan Africa | 55,040.74 (40,818.23 to 71,694.65) | 374.41 (278.13 to 487.28) | 138,042.14 (98,660.47 to 187,304.99) | 368.11 (263.03 to 499.38) | -0.09 (-0.28 to 0.11) |
| DALYs | East Asia | 555,814.31 (465,268.61 to 648,398.43) | 146.59 (122.74 to 170.94) | 195,208.81 (155,897.50 to 238,952.63) | 77.85 (62.32 to 95.21) | -2.03 (-2.28 to -1.78) |
| DALYs | Eastern Europe | 52,478.48 (47,328.81 to 59,667.50) | 105.45 (95.07 to 119.99) | 18,286.79 (14,750.18 to 22,779.08) | 55.76 (44.88 to 69.60) | -2.10 (-2.60 to -1.60) |
| DALYs | Eastern Sub-Saharan Africa | 187,194.22 (152,373.10 to 219,589.50) | 368.33 (300.95 to 432.79) | 400,937.00 (329,593.79 to 489,454.26) | 325.21 (267.72 to 396.45) | -0.39 (-0.48 to -0.31) |
| DALYs | High-income Asia Pacific | 21,868.28 (18,530.75 to 25,496.96) | 52.30 (44.32 to 60.97) | 7,863.59 (6,262.25 to 9,802.07) | 26.85 (21.41 to 33.51) | -2.13 (-2.35 to -1.91) |
| DALYs | High-income North America | 31,074.78 (24,186.90 to 39,693.56) | 44.99 (35.05 to 57.47) | 46,656.73 (36,995.43 to 58,860.31) | 62.30 (49.36 to 78.70) | 1.08 (0.85 to 1.31) |
| DALYs | North Africa and Middle East | 147,643.15 (120,809.08 to 189,631.94) | 160.37 (131.23 to 205.85) | 245,763.36 (205,312.64 to 292,496.54) | 160.67 (134.23 to 191.22) | 0.04 (-0.13 to 0.20) |
| DALYs | Oceania | 3,276.25 (1,953.15 to 4,635.30) | 180.45 (107.93 to 255.01) | 8,266.80 (5,984.05 to 10,823.60) | 223.23 (161.70 to 292.13) | 0.74 (0.59 to 0.88) |
| DALYs | South Asia | 361,562.21 (304,761.78 to 439,466.55) | 125.10 (105.37 to 152.05) | 608,231.23 (507,004.33 to 748,068.88) | 119.92 (99.96 to 147.48) | -0.10 (-0.42 to 0.21) |
| DALYs | Southeast Asia | 405,196.80 (312,372.48 to 475,405.88) | 305.23 (235.78 to 357.93) | 500,915.47 (392,567.76 to 586,405.78) | 292.66 (229.11 to 342.53) | -0.15 (-0.24 to -0.06) |
| DALYs | Southern Latin America | 13,593.84 (12,237.12 to 15,234.68) | 111.34 (100.23 to 124.79) | 12,983.18 (11,245.09 to 15,057.26) | 81.42 (70.54 to 94.40) | -0.96 (-1.20 to -0.71) |
| DALYs | Southern Sub-Saharan Africa | 23,469.89 (19,649.56 to 29,522.49) | 161.78 (135.53 to 203.36) | 35,578.21 (29,660.65 to 44,061.04) | 169.72 (141.46 to 210.20) | 0.00 (-0.47 to 0.48) |
| DALYs | Tropical Latin America | 57,604.52 (51,732.89 to 64,955.78) | 134.82 (121.08 to 152.00) | 47,006.19 (40,912.32 to 55,098.55) | 88.29 (76.85 to 103.50) | -1.28 (-1.50 to -1.07) |
| DALYs | Western Europe | 35,539.74 (25,714.64 to 47,825.97) | 38.87 (28.18 to 52.25) | 26,443.77 (17,914.92 to 36,345.45) | 34.80 (23.61 to 47.85) | -0.36 (-0.51 to -0.21) |
| DALYs | Western Sub-Saharan Africa | 150,574.52 (117,933.62 to 188,054.19) | 302.33 (236.95 to 378.40) | 402,822.54 (304,085.26 to 514,958.39) | 304.76 (230.36 to 390.25) | 0.02 (-0.08 to 0.12) |
|  | **Country** | | | | | |
| DALYs | Afghanistan | 7,722.05 (5,113.23 to 11,992.19) | 336.73 (222.39 to 516.07) | 25,915.22 (15,408.58 to 42,525.76) | 286.59 (170.55 to 468.28) | -0.52 (-0.92 to -0.11) |
| DALYs | Albania | 1,089.87 (864.12 to 1,380.70) | 115.60 (91.65 to 146.45) | 419.31 (297.92 to 599.30) | 69.80 (49.45 to 100.13) | -1.75 (-2.41 to -1.09) |
| DALYs | Algeria | 9,674.01 (6,935.18 to 13,850.99) | 135.68 (97.21 to 194.00) | 12,710.15 (9,271.12 to 16,537.66) | 128.74 (93.98 to 167.61) | -0.12 (-0.35 to 0.12) |
| DALYs | American Samoa | 30.31 (20.97 to 42.31) | 218.29 (150.94 to 304.78) | 55.64 (38.91 to 75.51) | 519.08 (362.70 to 703.78) | 2.90 (2.55 to 3.24) |
| DALYs | Andorra | 6.05 (4.04 to 8.71) | 39.14 (26.31 to 56.11) | 4.87 (3.18 to 7.06) | 34.57 (22.59 to 50.05) | -0.42 (-0.61 to -0.22) |
| DALYs | Angola | 9,805.98 (6,672.56 to 14,059.39) | 359.40 (245.45 to 514.13) | 29,724.76 (19,800.71 to 43,865.98) | 351.45 (234.60 to 517.15) | 0.01 (-0.60 to 0.62) |
| DALYs | Antigua and Barbuda | 32.48 (27.97 to 37.49) | 190.15 (163.75 to 219.51) | 51.86 (44.50 to 59.99) | 247.86 (212.58 to 286.59) | 0.79 (-0.16 to 1.75) |
| DALYs | Argentina | 9,912.10 (8,867.49 to 11,189.07) | 128.50 (114.97 to 145.05) | 9,850.94 (8,529.58 to 11,442.46) | 90.75 (78.59 to 105.41) | -1.10 (-1.36 to -0.84) |
| DALYs | Armenia | 556.46 (382.70 to 798.61) | 61.45 (42.21 to 88.44) | 719.40 (566.02 to 916.02) | 125.87 (98.97 to 160.81) | 2.38 (0.83 to 3.95) |
| DALYs | Australia | 1,015.06 (779.14 to 1,322.90) | 24.47 (18.80 to 31.87) | 1,231.15 (904.55 to 1,687.74) | 23.94 (17.60 to 32.77) | -0.01 (-0.58 to 0.57) |
| DALYs | Austria | 681.95 (478.36 to 954.54) | 34.66 (24.45 to 48.40) | 614.29 (416.60 to 859.31) | 36.69 (24.98 to 51.19) | 0.21 (-0.16 to 0.58) |
| DALYs | Azerbaijan | 4,529.89 (3,528.70 to 5,761.52) | 210.74 (163.95 to 268.42) | 5,201.69 (3,919.75 to 6,846.50) | 218.02 (164.18 to 288.14) | 0.09 (-0.50 to 0.68) |
| DALYs | Bahamas | 170.55 (147.18 to 198.86) | 214.45 (185.03 to 250.17) | 291.61 (228.40 to 370.42) | 303.42 (237.72 to 385.40) | 1.08 (0.02 to 2.16) |
| DALYs | Bahrain | 192.09 (140.51 to 262.44) | 128.81 (93.64 to 176.52) | 571.86 (435.65 to 763.40) | 142.86 (108.98 to 189.72) | 0.24 (-0.45 to 0.94) |
| DALYs | Bangladesh | 37,163.97 (27,874.38 to 49,783.56) | 127.86 (96.03 to 170.56) | 41,305.94 (30,012.36 to 58,756.90) | 93.65 (68.04 to 133.28) | -0.86 (-1.19 to -0.54) |
| DALYs | Barbados | 112.30 (98.95 to 127.91) | 163.81 (144.33 to 186.58) | 106.35 (82.27 to 136.08) | 179.99 (139.05 to 230.36) | 0.48 (-0.70 to 1.68) |
| DALYs | Belarus | 851.01 (625.41 to 1,169.17) | 36.22 (26.57 to 49.87) | 551.71 (400.27 to 755.79) | 37.25 (26.93 to 51.20) | -0.03 (-0.66 to 0.61) |
| DALYs | Belgium | 898.66 (605.01 to 1,264.77) | 39.08 (26.37 to 54.92) | 782.87 (508.91 to 1,138.19) | 37.54 (24.44 to 54.56) | -0.19 (-0.46 to 0.08) |
| DALYs | Belize | 101.09 (88.03 to 116.95) | 198.70 (173.12 to 229.77) | 437.30 (371.20 to 521.54) | 355.72 (302.07 to 423.25) | 1.89 (1.17 to 2.62) |
| DALYs | Benin | 3,890.49 (2,789.88 to 5,276.56) | 328.97 (236.35 to 445.77) | 12,264.10 (8,305.61 to 17,355.14) | 334.77 (226.69 to 473.81) | 0.07 (-0.19 to 0.34) |
| DALYs | Bermuda | 15.20 (12.97 to 17.84) | 97.98 (83.50 to 115.26) | 8.85 (7.23 to 10.88) | 93.89 (76.64 to 115.39) | -0.24 (-1.24 to 0.77) |
| DALYs | Bhutan | 266.23 (170.51 to 383.86) | 139.63 (89.71 to 200.65) | 300.93 (201.92 to 434.77) | 137.64 (92.35 to 198.77) | -0.08 (-0.24 to 0.08) |
| DALYs | Bolivia (Plurinational State of) | 4,543.09 (3,257.42 to 6,528.56) | 272.09 (194.79 to 390.74) | 6,777.36 (4,497.33 to 10,072.26) | 213.88 (141.92 to 317.74) | -0.79 (-0.96 to -0.63) |
| DALYs | Bosnia and Herzegovina | 995.53 (798.57 to 1,233.59) | 83.85 (67.17 to 104.03) | 414.24 (307.29 to 547.83) | 71.12 (52.70 to 94.30) | -0.58 (-1.21 to 0.06) |
| DALYs | Botswana | 449.63 (287.79 to 770.16) | 125.58 (79.93 to 217.76) | 812.93 (527.81 to 1,357.19) | 125.62 (81.60 to 209.28) | -0.04 (-0.69 to 0.62) |
| DALYs | Brazil | 56,581.36 (50,811.11 to 63,773.22) | 135.74 (121.90 to 152.97) | 44,814.54 (39,074.03 to 52,563.93) | 87.32 (76.14 to 102.42) | -1.34 (-1.56 to -1.13) |
| DALYs | Brunei Darussalam | 122.98 (88.92 to 159.81) | 154.98 (111.86 to 201.41) | 123.63 (93.90 to 160.77) | 101.11 (76.75 to 131.63) | -1.32 (-1.72 to -0.91) |
| DALYs | Bulgaria | 1,768.58 (1,496.36 to 2,104.18) | 100.06 (84.67 to 119.02) | 1,235.12 (954.52 to 1,600.53) | 125.48 (96.96 to 162.40) | 0.69 (0.09 to 1.29) |
| DALYs | Burkina Faso | 7,372.70 (5,161.47 to 10,392.50) | 328.03 (230.19 to 461.39) | 21,899.53 (15,106.11 to 31,178.62) | 369.43 (255.58 to 524.94) | 0.43 (0.13 to 0.72) |
| DALYs | Burundi | 5,637.26 (3,828.05 to 8,061.06) | 393.21 (267.31 to 562.53) | 10,597.17 (7,079.00 to 16,499.53) | 298.11 (199.25 to 465.07) | -0.95 (-1.36 to -0.54) |
| DALYs | Cabo Verde | 189.04 (137.66 to 256.94) | 199.93 (145.60 to 271.27) | 284.52 (191.89 to 402.77) | 187.26 (125.99 to 265.14) | -0.22 (-0.48 to 0.04) |
| DALYs | Cambodia | 9,465.99 (6,232.84 to 13,000.52) | 352.28 (232.44 to 483.62) | 12,753.11 (8,118.62 to 19,528.42) | 281.20 (178.90 to 430.87) | -0.71 (-0.86 to -0.56) |
| DALYs | Cameroon | 12,135.34 (8,313.54 to 17,168.70) | 458.50 (314.65 to 647.13) | 40,717.12 (26,230.45 to 60,708.10) | 468.13 (302.43 to 695.95) | 0.06 (-0.06 to 0.18) |
| DALYs | Canada | 1,669.72 (1,188.20 to 2,370.57) | 24.74 (17.61 to 35.04) | 3,068.58 (2,325.31 to 4,060.68) | 43.22 (32.71 to 57.34) | 1.83 (1.55 to 2.11) |
| DALYs | Central African Republic | 3,313.15 (2,327.03 to 4,580.25) | 448.22 (315.52 to 618.80) | 7,050.12 (4,635.56 to 11,091.71) | 461.04 (303.30 to 725.36) | -0.03 (-0.40 to 0.34) |
| DALYs | Chad | 3,642.66 (2,488.19 to 5,657.81) | 247.93 (169.77 to 385.80) | 12,991.46 (8,680.53 to 20,173.79) | 288.23 (192.90 to 447.29) | 0.57 (0.17 to 0.96) |
| DALYs | Chile | 3,279.64 (2,836.17 to 3,832.45) | 86.80 (75.07 to 101.43) | 2,625.17 (2,152.85 to 3,167.90) | 60.25 (49.50 to 72.69) | -1.11 (-1.85 to -0.38) |
| DALYs | China | 538,228.36 (448,809.97 to 628,942.17) | 146.31 (122.06 to 170.95) | 182,834.92 (144,950.06 to 224,769.97) | 76.10 (60.44 to 93.47) | -2.10 (-2.35 to -1.86) |
| DALYs | Colombia | 17,559.84 (15,477.27 to 19,865.51) | 184.40 (162.50 to 208.64) | 11,886.73 (9,657.02 to 14,523.48) | 92.44 (75.16 to 112.82) | -2.27 (-3.01 to -1.53) |
| DALYs | Comoros | 421.68 (168.49 to 643.79) | 337.88 (138.19 to 513.70) | 693.76 (476.53 to 992.69) | 339.32 (233.22 to 485.16) | -0.21 (-3.66 to 3.37) |
| DALYs | Congo | 3,152.73 (2,048.90 to 4,374.20) | 463.34 (301.92 to 641.66) | 6,656.00 (4,434.93 to 9,470.45) | 461.44 (306.96 to 657.78) | 0.21 (-0.35 to 0.77) |
| DALYs | Cook Islands | 5.71 (4.10 to 7.96) | 109.24 (78.36 to 152.56) | 4.89 (3.51 to 6.71) | 129.33 (92.90 to 177.49) | 0.58 (0.39 to 0.77) |
| DALYs | Costa Rica | 867.52 (703.72 to 1,081.45) | 100.83 (81.81 to 125.69) | 1,750.02 (1,480.59 to 2,084.60) | 150.92 (127.69 to 179.74) | 1.24 (0.58 to 1.90) |
| DALYs | Coted'Ivoire | 11,450.05 (7,590.20 to 16,243.34) | 345.20 (229.15 to 489.43) | 26,920.51 (17,582.62 to 38,682.54) | 365.20 (238.57 to 525.09) | 0.18 (-0.29 to 0.66) |
| DALYs | Croatia | 783.45 (664.31 to 946.38) | 73.89 (62.57 to 89.39) | 371.60 (280.55 to 502.50) | 52.47 (39.65 to 70.85) | -1.08 (-1.49 to -0.68) |
| DALYs | Cuba | 3,409.30 (2,933.16 to 4,012.95) | 99.83 (85.90 to 117.50) | 1,947.60 (1,597.54 to 2,366.68) | 92.94 (76.29 to 112.89) | -0.20 (-0.81 to 0.41) |
| DALYs | Cyprus | 89.67 (61.29 to 126.11) | 46.24 (31.62 to 65.02) | 100.26 (64.46 to 143.04) | 36.09 (23.12 to 51.76) | -0.78 (-1.09 to -0.47) |
| DALYs | Czechia | 1,460.63 (1,191.81 to 1,860.72) | 67.16 (54.90 to 85.35) | 703.24 (514.90 to 953.72) | 45.32 (33.11 to 61.61) | -1.33 (-1.91 to -0.76) |
| DALYs | Democratic People's Republic of Korea | 9,523.35 (6,508.00 to 13,928.43) | 169.52 (115.80 to 247.88) | 9,073.04 (6,408.89 to 13,006.91) | 148.38 (104.96 to 212.28) | -0.44 (-0.50 to -0.38) |
| DALYs | Democratic Republic of the Congo | 37,270.07 (25,967.95 to 51,216.97) | 366.28 (255.50 to 502.75) | 90,027.71 (58,172.64 to 130,147.28) | 358.92 (232.39 to 517.77) | -0.10 (-0.41 to 0.22) |
| DALYs | Denmark | 367.22 (236.88 to 541.40) | 30.73 (19.85 to 45.28) | 391.02 (236.12 to 599.16) | 33.48 (20.25 to 51.28) | 0.33 (0.12 to 0.55) |
| DALYs | Djibouti | 264.58 (172.78 to 392.37) | 209.77 (137.21 to 311.52) | 939.82 (595.97 to 1,463.53) | 281.87 (178.77 to 439.10) | 1.05 (0.42 to 1.68) |
| DALYs | Dominica | 37.96 (29.63 to 47.57) | 187.54 (146.34 to 235.22) | 53.30 (38.58 to 71.32) | 332.12 (240.46 to 444.49) | 1.85 (1.64 to 2.06) |
| DALYs | Dominican Republic | 3,523.55 (2,819.64 to 4,351.49) | 165.00 (132.04 to 203.72) | 6,803.48 (4,625.11 to 8,920.98) | 234.26 (159.24 to 307.41) | 1.08 (0.39 to 1.77) |
| DALYs | Ecuador | 5,738.85 (5,117.08 to 6,561.17) | 203.04 (180.90 to 232.25) | 7,653.79 (6,058.19 to 9,691.52) | 161.95 (128.28 to 205.04) | -0.74 (-2.26 to 0.81) |
| DALYs | Egypt | 28,376.96 (20,867.68 to 40,344.59) | 191.64 (141.03 to 272.26) | 60,085.27 (46,311.11 to 76,068.51) | 224.51 (173.05 to 284.20) | 0.57 (0.02 to 1.13) |
| DALYs | El Salvador | 3,583.40 (2,846.41 to 4,675.75) | 246.03 (195.49 to 320.86) | 7,934.66 (5,680.09 to 10,634.62) | 451.71 (323.21 to 605.75) | 1.81 (0.40 to 3.23) |
| DALYs | Equatorial Guinea | 446.63 (299.50 to 633.41) | 415.08 (280.29 to 586.69) | 2,115.11 (1,156.65 to 3,544.27) | 435.44 (238.14 to 728.70) | 0.18 (-0.51 to 0.88) |
| DALYs | Eritrea | 3,022.62 (1,887.62 to 4,749.13) | 322.99 (201.88 to 507.84) | 6,028.21 (3,672.24 to 9,807.27) | 319.83 (194.66 to 520.89) | -0.08 (-0.39 to 0.24) |
| DALYs | Estonia | 517.54 (454.93 to 585.35) | 154.26 (135.51 to 174.49) | 276.40 (224.97 to 357.19) | 135.50 (110.67 to 174.47) | -0.95 (-2.64 to 0.77) |
| DALYs | Eswatini | 376.48 (269.55 to 536.63) | 176.58 (126.69 to 251.69) | 937.93 (545.08 to 1,433.38) | 279.71 (162.63 to 427.77) | 1.48 (1.22 to 1.75) |
| DALYs | Ethiopia | 75,097.95 (54,199.88 to 93,406.37) | 578.29 (419.20 to 718.08) | 97,616.68 (75,865.97 to 122,392.63) | 298.24 (231.75 to 373.91) | -2.13 (-2.26 to -2.00) |
| DALYs | Fiji | 529.34 (361.21 to 766.78) | 246.96 (168.64 to 357.55) | 810.29 (577.90 to 1,099.66) | 365.48 (260.66 to 495.98) | 1.29 (1.14 to 1.45) |
| DALYs | Finland | 256.91 (158.23 to 385.42) | 23.92 (14.80 to 35.77) | 250.18 (149.62 to 382.69) | 25.43 (15.26 to 38.86) | 0.23 (-0.05 to 0.51) |
| DALYs | France | 3,492.34 (2,580.81 to 4,630.47) | 26.08 (19.28 to 34.57) | 2,597.75 (1,825.20 to 3,699.19) | 22.12 (15.53 to 31.50) | -0.52 (-0.72 to -0.33) |
| DALYs | Gabon | 1,052.17 (746.92 to 1,428.18) | 393.14 (279.42 to 533.30) | 2,468.43 (1,338.11 to 3,815.15) | 488.07 (264.10 to 754.58) | 0.73 (0.55 to 0.92) |
| DALYs | Gambia | 844.53 (557.70 to 1,209.96) | 316.37 (209.17 to 453.43) | 2,937.00 (1,916.78 to 4,298.76) | 423.57 (277.05 to 619.84) | 0.89 (-1.01 to 2.82) |
| DALYs | Georgia | 1,748.08 (1,373.43 to 2,164.90) | 130.63 (102.44 to 162.08) | 1,349.11 (1,116.25 to 1,664.86) | 215.57 (178.33 to 265.95) | 1.42 (-0.36 to 3.23) |
| DALYs | Germany | 7,243.75 (5,129.51 to 9,980.57) | 37.91 (27.05 to 52.08) | 4,891.90 (3,214.50 to 6,974.40) | 33.53 (22.15 to 47.66) | -0.36 (-0.72 to 0.01) |
| DALYs | Ghana | 13,194.44 (8,864.05 to 19,864.71) | 334.12 (224.89 to 501.93) | 43,423.39 (28,882.75 to 63,327.37) | 457.00 (304.20 to 666.16) | 1.04 (0.83 to 1.24) |
| DALYs | Greece | 1,108.78 (834.80 to 1,444.21) | 47.31 (35.62 to 61.64) | 735.42 (535.17 to 972.24) | 47.19 (34.36 to 62.39) | 0.08 (-0.15 to 0.32) |
| DALYs | Greenland | 6.30 (4.49 to 8.82) | 34.32 (24.48 to 47.89) | 4.44 (3.12 to 6.08) | 35.33 (24.90 to 48.40) | 0.10 (-0.02 to 0.23) |
| DALYs | Grenada | 72.19 (63.00 to 82.25) | 326.57 (285.21 to 371.78) | 93.25 (77.40 to 112.47) | 343.83 (285.64 to 414.45) | 0.05 (-1.28 to 1.40) |
| DALYs | Guam | 57.40 (44.98 to 75.67) | 137.24 (107.42 to 180.85) | 69.23 (53.43 to 88.49) | 193.78 (149.46 to 247.64) | 0.97 (0.22 to 1.72) |
| DALYs | Guatemala | 5,549.55 (4,973.55 to 6,267.18) | 274.45 (246.12 to 309.70) | 17,559.07 (14,841.90 to 20,594.20) | 378.23 (319.64 to 443.77) | 1.23 (-0.75 to 3.26) |
| DALYs | Guinea | 4,110.36 (2,882.17 to 5,996.58) | 298.68 (209.33 to 435.66) | 11,517.90 (7,915.84 to 17,210.90) | 326.31 (224.11 to 486.52) | 0.27 (0.06 to 0.48) |
| DALYs | Guinea-Bissau | 1,479.67 (1,009.67 to 2,048.38) | 567.19 (389.00 to 783.61) | 2,784.01 (1,897.85 to 3,962.78) | 488.05 (332.69 to 695.29) | -0.47 (-0.53 to -0.41) |
| DALYs | Guyana | 536.66 (428.79 to 638.78) | 230.37 (184.21 to 274.10) | 788.33 (559.57 to 1,059.19) | 366.18 (260.50 to 491.38) | 1.78 (1.01 to 2.54) |
| DALYs | Haiti | 4,400.45 (2,960.49 to 7,042.38) | 271.76 (182.87 to 435.06) | 8,936.58 (5,025.77 to 18,788.08) | 257.26 (144.75 to 540.57) | -0.03 (-0.63 to 0.58) |
| DALYs | Honduras | 1,467.30 (1,121.13 to 1,925.06) | 125.92 (96.26 to 165.32) | 2,891.45 (1,836.32 to 4,326.70) | 97.29 (61.73 to 145.55) | -0.88 (-1.27 to -0.48) |
| DALYs | Hungary | 1,430.25 (1,169.48 to 1,787.55) | 69.91 (57.26 to 87.14) | 725.35 (539.38 to 987.50) | 46.13 (34.10 to 63.09) | -1.50 (-1.77 to -1.23) |
| DALYs | Iceland | 13.18 (8.83 to 18.92) | 20.14 (13.51 to 28.87) | 17.33 (11.20 to 25.55) | 23.23 (14.97 to 34.32) | 0.41 (0.12 to 0.70) |
| DALYs | India | 272,413.51 (226,181.14 to 337,594.78) | 120.24 (99.79 to 149.06) | 399,192.67 (322,523.48 to 504,820.57) | 102.85 (83.10 to 130.05) | -0.48 (-0.80 to -0.17) |
| DALYs | Indonesia | 160,360.99 (111,001.60 to 194,864.32) | 305.03 (211.64 to 370.29) | 212,737.18 (152,537.95 to 277,377.15) | 303.31 (217.43 to 395.04) | -0.02 (-0.11 to 0.07) |
| DALYs | Iran (Islamic Republic of) | 14,850.57 (11,935.02 to 19,646.84) | 97.90 (78.67 to 129.57) | 17,414.76 (14,578.41 to 20,767.62) | 98.46 (82.48 to 117.19) | 0.02 (-0.21 to 0.24) |
| DALYs | Iraq | 10,321.08 (7,585.45 to 13,998.40) | 205.07 (151.01 to 277.88) | 15,755.72 (9,700.40 to 22,790.19) | 136.39 (83.95 to 197.47) | -1.36 (-1.63 to -1.09) |
| DALYs | Ireland | 306.08 (204.84 to 443.16) | 36.00 (24.08 to 52.19) | 356.01 (220.42 to 531.02) | 40.01 (24.79 to 59.67) | 0.31 (0.03 to 0.58) |
| DALYs | Israel | 652.22 (485.78 to 885.88) | 55.15 (41.00 to 74.99) | 1,043.77 (750.57 to 1,396.58) | 50.84 (36.54 to 68.05) | -0.22 (-0.77 to 0.33) |
| DALYs | Italy | 5,951.61 (4,143.42 to 8,334.53) | 43.28 (30.16 to 60.60) | 3,759.29 (2,372.69 to 5,542.81) | 40.99 (25.88 to 60.45) | -0.28 (-0.50 to -0.07) |
| DALYs | Jamaica | 932.19 (803.14 to 1,109.28) | 136.38 (117.47 to 162.35) | 1,399.02 (1,017.15 to 1,851.64) | 180.03 (131.22 to 238.04) | 0.87 (-0.37 to 2.13) |
| DALYs | Japan | 10,819.22 (9,189.26 to 12,857.20) | 39.79 (33.80 to 47.27) | 5,174.07 (4,081.89 to 6,552.76) | 27.69 (21.86 to 35.10) | -1.17 (-1.34 to -1.00) |
| DALYs | Jordan | 1,710.72 (1,292.98 to 2,247.63) | 150.04 (113.29 to 197.32) | 4,326.57 (3,293.53 to 5,535.58) | 123.39 (93.93 to 157.88) | -0.61 (-1.15 to -0.07) |
| DALYs | Kazakhstan | 8,407.89 (7,142.38 to 10,008.98) | 195.86 (166.44 to 233.27) | 5,708.66 (4,417.22 to 7,544.27) | 144.26 (111.62 to 190.34) | -1.04 (-1.63 to -0.44) |
| DALYs | Kenya | 12,432.27 (9,451.65 to 18,150.44) | 194.32 (147.54 to 284.24) | 37,620.49 (29,209.62 to 51,260.67) | 252.64 (196.09 to 344.29) | 0.86 (0.71 to 1.00) |
| DALYs | Kiribati | 64.10 (47.89 to 83.75) | 304.80 (227.65 to 398.22) | 132.55 (82.93 to 208.81) | 412.56 (258.09 to 649.74) | 0.99 (0.91 to 1.07) |
| DALYs | Kuwait | 743.98 (629.02 to 870.22) | 146.22 (123.98 to 170.60) | 785.87 (611.84 to 998.95) | 77.08 (60.37 to 97.67) | -1.78 (-2.45 to -1.09) |
| DALYs | Kyrgyzstan | 2,815.19 (2,334.70 to 3,377.42) | 233.97 (194.12 to 280.62) | 3,834.57 (3,175.50 to 4,666.12) | 223.43 (184.91 to 272.06) | -0.30 (-1.13 to 0.54) |
| DALYs | Lao People's Democratic Republic | 4,743.11 (3,265.76 to 6,651.68) | 451.30 (311.22 to 633.76) | 7,876.79 (5,204.74 to 12,027.03) | 382.26 (252.48 to 583.71) | -0.53 (-0.71 to -0.36) |
| DALYs | Latvia | 459.05 (393.25 to 541.77) | 79.30 (67.82 to 93.77) | 200.50 (158.34 to 260.04) | 71.58 (56.56 to 92.72) | -0.43 (-1.25 to 0.40) |
| DALYs | Lebanon | 1,190.78 (841.82 to 1,700.15) | 151.06 (106.79 to 215.93) | 1,474.37 (1,114.18 to 1,902.01) | 111.28 (84.02 to 143.81) | -0.96 (-1.19 to -0.73) |
| DALYs | Lesotho | 325.55 (231.56 to 462.07) | 89.65 (63.53 to 127.85) | 1,207.18 (798.43 to 1,758.81) | 216.49 (143.26 to 315.62) | 2.96 (2.70 to 3.21) |
| DALYs | Liberia | 2,931.11 (1,830.25 to 5,202.06) | 466.40 (290.80 to 830.70) | 6,978.75 (4,690.57 to 9,990.07) | 466.54 (314.25 to 667.32) | 0.26 (-0.23 to 0.75) |
| DALYs | Libya | 1,724.48 (1,257.91 to 2,360.71) | 146.75 (107.26 to 200.84) | 3,528.43 (2,294.46 to 4,898.46) | 199.81 (129.96 to 277.46) | 1.03 (0.43 to 1.62) |
| DALYs | Lithuania | 609.52 (509.30 to 739.94) | 69.77 (58.24 to 84.77) | 306.81 (233.13 to 419.81) | 64.21 (48.94 to 87.63) | -0.33 (-1.15 to 0.49) |
| DALYs | Luxembourg | 38.86 (27.04 to 55.47) | 43.31 (30.31 to 61.62) | 44.74 (28.21 to 66.17) | 35.36 (22.39 to 52.19) | -0.61 (-1.21 to 0.00) |
| DALYs | Madagascar | 8,713.09 (6,269.29 to 11,927.60) | 272.95 (196.64 to 373.49) | 21,969.02 (14,925.83 to 31,127.28) | 268.14 (182.89 to 378.74) | -0.03 (-0.36 to 0.31) |
| DALYs | Malawi | 9,916.30 (6,844.26 to 13,671.50) | 365.61 (252.87 to 503.36) | 24,857.51 (17,010.29 to 34,970.56) | 426.20 (291.92 to 597.51) | 0.55 (0.32 to 0.78) |
| DALYs | Malaysia | 8,893.11 (6,872.40 to 11,366.57) | 182.46 (140.94 to 233.15) | 13,888.89 (10,500.04 to 17,828.40) | 160.75 (121.41 to 206.53) | -0.59 (-0.93 to -0.26) |
| DALYs | Maldives | 204.34 (153.43 to 262.66) | 349.48 (261.99 to 448.02) | 231.16 (168.03 to 299.34) | 176.03 (129.27 to 227.87) | -2.08 (-2.66 to -1.49) |
| DALYs | Mali | 7,121.24 (5,201.50 to 9,709.65) | 347.59 (254.17 to 474.29) | 20,451.51 (14,530.57 to 28,805.17) | 323.39 (229.69 to 455.84) | -0.23 (-0.41 to -0.05) |
| DALYs | Malta | 34.37 (23.82 to 47.13) | 43.08 (29.88 to 59.07) | 30.39 (20.00 to 43.79) | 40.98 (27.27 to 58.83) | 0.18 (0.04 to 0.32) |
| DALYs | Marshall Islands | 34.09 (21.84 to 55.53) | 301.75 (193.78 to 488.55) | 72.30 (25.90 to 203.51) | 487.28 (174.75 to 1,366.39) | 1.59 (1.40 to 1.77) |
| DALYs | Mauritania | 1,905.94 (1,327.76 to 2,568.28) | 359.66 (251.24 to 484.22) | 3,209.75 (2,078.50 to 4,736.73) | 272.64 (176.55 to 401.42) | -0.97 (-1.14 to -0.81) |
| DALYs | Mauritius | 922.22 (807.69 to 1,049.77) | 286.51 (250.94 to 326.09) | 1,650.89 (1,425.13 to 1,874.13) | 578.67 (498.64 to 657.72) | 2.17 (0.73 to 3.64) |
| DALYs | Mexico | 61,645.96 (57,347.00 to 67,543.30) | 250.02 (232.23 to 274.15) | 117,085.92 (103,660.59 to 134,491.74) | 361.43 (320.00 to 415.10) | 1.20 (0.94 to 1.47) |
| DALYs | Micronesia (Federated States of) | 74.50 (48.67 to 113.79) | 286.24 (187.05 to 435.57) | 130.76 (87.19 to 191.57) | 464.52 (309.43 to 680.73) | 1.59 (1.50 to 1.69) |
| DALYs | Monaco | 1.76 (1.19 to 2.49) | 32.24 (21.88 to 45.31) | 2.11 (1.43 to 2.89) | 38.83 (26.33 to 53.09) | 0.63 (0.54 to 0.73) |
| DALYs | Mongolia | 1,460.59 (1,095.68 to 2,072.21) | 234.52 (176.03 to 332.40) | 1,621.33 (1,254.50 to 2,094.38) | 224.99 (173.94 to 290.60) | -0.09 (-0.97 to 0.80) |
| DALYs | Montenegro | 172.11 (132.05 to 226.75) | 110.04 (84.40 to 145.08) | 117.83 (87.72 to 153.31) | 97.31 (72.35 to 126.80) | -0.18 (-1.70 to 1.36) |
| DALYs | Morocco | 9,550.51 (6,624.26 to 13,856.50) | 133.97 (92.99 to 193.98) | 11,400.63 (8,048.72 to 16,996.29) | 125.97 (88.94 to 187.69) | -0.24 (-0.46 to -0.02) |
| DALYs | Mozambique | 8,676.15 (5,814.48 to 13,590.19) | 261.46 (175.53 to 409.39) | 35,752.82 (23,467.24 to 53,422.50) | 408.47 (267.73 to 609.64) | 1.47 (1.30 to 1.64) |
| DALYs | Myanmar | 62,112.25 (39,547.03 to 87,161.79) | 531.16 (338.98 to 744.30) | 52,861.80 (36,355.10 to 72,518.42) | 365.78 (251.86 to 501.59) | -1.21 (-1.43 to -0.98) |
| DALYs | Namibia | 409.20 (282.70 to 686.02) | 103.85 (71.46 to 175.73) | 716.22 (453.43 to 1,153.12) | 103.86 (65.73 to 167.42) | 0.07 (-0.20 to 0.33) |
| DALYs | Nauru | 8.38 (5.43 to 12.42) | 320.75 (208.13 to 475.50) | 15.31 (10.39 to 21.48) | 505.84 (342.80 to 709.37) | 1.48 (1.37 to 1.59) |
| DALYs | Nepal | 7,666.05 (5,514.16 to 10,495.08) | 155.52 (111.88 to 213.00) | 14,815.81 (10,298.21 to 21,053.66) | 164.14 (114.09 to 233.01) | 0.17 (0.08 to 0.27) |
| DALYs | Netherlands | 1,474.08 (916.77 to 2,236.42) | 38.83 (24.18 to 58.98) | 1,174.16 (720.36 to 1,733.03) | 36.07 (22.14 to 53.31) | -0.24 (-0.40 to -0.07) |
| DALYs | New Zealand | 269.15 (212.65 to 341.74) | 31.18 (24.65 to 39.57) | 369.52 (282.60 to 485.46) | 33.13 (25.38 to 43.49) | 0.20 (-0.60 to 1.00) |
| DALYs | Nicaragua | 2,375.64 (1,851.31 to 3,108.08) | 230.28 (179.29 to 301.35) | 6,035.74 (4,575.37 to 7,719.52) | 329.34 (249.73 to 421.08) | 1.02 (0.75 to 1.29) |
| DALYs | Niger | 5,274.72 (3,576.97 to 8,099.27) | 269.85 (183.42 to 415.38) | 14,068.06 (8,883.78 to 24,066.96) | 217.90 (136.89 to 374.40) | -0.68 (-0.83 to -0.52) |
| DALYs | Nigeria | 61,541.70 (44,460.61 to 81,848.32) | 258.62 (186.86 to 344.79) | 151,828.32 (95,604.28 to 227,084.09) | 241.26 (152.00 to 362.03) | -0.25 (-0.40 to -0.10) |
| DALYs | Niue | 1.17 (0.81 to 1.66) | 223.33 (155.49 to 319.35) | 2.14 (1.25 to 3.47) | 592.72 (346.32 to 962.40) | 3.42 (3.08 to 3.76) |
| DALYs | North Macedonia | 461.26 (351.16 to 600.53) | 93.17 (70.90 to 121.35) | 333.00 (234.25 to 477.85) | 78.75 (55.38 to 112.92) | -0.58 (-0.87 to -0.28) |
| DALYs | Northern Mariana Islands | 32.43 (22.68 to 45.38) | 221.06 (154.80 to 308.62) | 27.18 (20.18 to 34.87) | 278.97 (207.11 to 357.84) | 0.95 (0.69 to 1.20) |
| DALYs | Norway | 215.44 (138.64 to 312.87) | 21.70 (13.99 to 31.49) | 293.83 (182.35 to 430.42) | 27.57 (17.11 to 40.42) | 0.75 (0.41 to 1.09) |
| DALYs | Oman | 517.56 (366.20 to 766.01) | 101.86 (72.24 to 150.40) | 1,412.01 (1,039.30 to 1,880.67) | 123.03 (91.06 to 165.44) | 0.63 (0.00 to 1.27) |
| DALYs | Pakistan | 44,052.45 (33,821.33 to 56,936.65) | 155.94 (119.95 to 201.50) | 152,615.88 (113,116.88 to 197,505.47) | 232.20 (172.05 to 300.59) | 1.29 (1.15 to 1.43) |
| DALYs | Palau | 11.13 (7.44 to 15.82) | 245.82 (164.25 to 349.46) | 18.97 (10.42 to 28.02) | 557.83 (306.16 to 824.13) | 2.70 (2.47 to 2.92) |
| DALYs | Palestine | 984.54 (687.19 to 1,416.36) | 175.21 (122.73 to 251.57) | 2,230.73 (1,727.16 to 2,905.68) | 148.56 (115.05 to 193.38) | -0.52 (-0.79 to -0.25) |
| DALYs | Panama | 888.75 (756.44 to 1,062.72) | 129.48 (110.18 to 154.91) | 1,905.43 (1,541.63 to 2,348.93) | 179.82 (145.47 to 221.66) | 1.03 (0.29 to 1.78) |
| DALYs | Papua New Guinea | 1,777.62 (834.36 to 2,682.00) | 156.20 (73.73 to 235.37) | 5,500.98 (3,691.17 to 7,655.50) | 194.69 (130.74 to 270.89) | 0.77 (0.54 to 0.99) |
| DALYs | Paraguay | 1,023.16 (810.38 to 1,299.54) | 97.99 (77.65 to 124.44) | 2,191.65 (1,630.39 to 2,882.00) | 112.86 (83.96 to 148.40) | 0.49 (-0.39 to 1.38) |
| DALYs | Peru | 12,675.10 (9,979.17 to 15,721.32) | 207.52 (163.55 to 257.59) | 15,117.01 (10,900.81 to 20,383.77) | 161.02 (116.12 to 216.86) | -0.83 (-1.89 to 0.24) |
| DALYs | Philippines | 57,695.31 (50,559.42 to 65,282.12) | 329.37 (288.29 to 372.67) | 117,843.58 (95,195.96 to 136,751.90) | 381.38 (308.05 to 442.54) | 0.44 (0.25 to 0.64) |
| DALYs | Poland | 8,223.92 (6,955.64 to 10,068.02) | 103.63 (87.69 to 126.87) | 2,626.83 (1,903.34 to 3,658.39) | 42.00 (30.34 to 58.80) | -2.92 (-3.23 to -2.61) |
| DALYs | Portugal | 1,454.20 (1,113.35 to 1,901.70) | 60.96 (46.64 to 79.73) | 672.78 (494.68 to 922.26) | 39.07 (28.73 to 53.52) | -1.39 (-1.82 to -0.95) |
| DALYs | Puerto Rico | 1,762.98 (1,581.32 to 1,992.46) | 196.42 (176.24 to 221.84) | 1,333.08 (1,102.46 to 1,588.04) | 209.38 (173.08 to 249.51) | 0.25 (-0.61 to 1.11) |
| DALYs | Qatar | 145.06 (92.53 to 221.08) | 118.86 (75.30 to 181.18) | 728.10 (529.89 to 1,010.22) | 101.96 (74.00 to 141.23) | -0.59 (-1.24 to 0.06) |
| DALYs | Republic of Korea | 10,454.00 (7,845.58 to 12,650.61) | 77.20 (57.93 to 93.42) | 2,235.16 (1,719.44 to 2,918.36) | 23.37 (17.98 to 30.51) | -3.68 (-4.30 to -3.06) |
| DALYs | Republic of Moldova | 667.62 (505.02 to 883.54) | 65.79 (49.77 to 87.15) | 418.08 (302.99 to 572.37) | 67.32 (48.48 to 92.57) | -0.07 (-0.64 to 0.50) |
| DALYs | Romania | 5,438.17 (4,635.74 to 6,486.45) | 106.89 (91.23 to 127.10) | 1,730.70 (1,313.07 to 2,282.10) | 58.45 (44.35 to 77.06) | -1.90 (-2.63 to -1.16) |
| DALYs | Russian Federation | 46,987.78 (43,217.20 to 52,283.74) | 140.71 (129.39 to 156.65) | 11,558.82 (9,247.68 to 14,620.14) | 50.81 (40.60 to 64.36) | -3.30 (-3.78 to -2.82) |
| DALYs | Rwanda | 9,269.94 (6,756.56 to 12,587.82) | 490.46 (358.44 to 664.50) | 10,976.53 (7,274.14 to 16,320.02) | 289.04 (191.73 to 429.54) | -1.66 (-2.03 to -1.28) |
| DALYs | Saint Kitts and Nevis | 29.89 (25.57 to 35.48) | 266.28 (227.66 to 315.96) | 33.81 (26.15 to 45.18) | 254.49 (197.19 to 338.81) | -0.18 (-1.01 to 0.66) |
| DALYs | Saint Lucia | 83.80 (72.70 to 95.63) | 214.10 (185.80 to 244.11) | 118.06 (93.19 to 143.63) | 289.54 (228.49 to 352.49) | 0.92 (0.33 to 1.52) |
| DALYs | Saint Vincent and the Grenadines | 65.71 (57.17 to 76.55) | 206.29 (179.75 to 240.16) | 82.73 (68.59 to 99.08) | 317.72 (263.29 to 380.46) | 1.32 (-0.16 to 2.83) |
| DALYs | Samoa | 99.25 (66.01 to 149.53) | 209.56 (139.11 to 316.11) | 163.55 (110.30 to 229.05) | 309.84 (208.68 to 434.12) | 1.26 (1.15 to 1.38) |
| DALYs | San Marino | 1.67 (1.04 to 2.49) | 27.17 (16.99 to 40.47) | 1.50 (0.91 to 2.25) | 27.46 (16.70 to 41.20) | 0.03 (-0.09 to 0.14) |
| DALYs | Sao Tome and Principe | 96.70 (49.80 to 157.45) | 314.44 (164.25 to 506.15) | 293.40 (173.95 to 443.12) | 487.38 (287.62 to 738.07) | 1.31 (0.68 to 1.94) |
| DALYs | Saudi Arabia | 8,796.00 (6,102.02 to 12,547.44) | 196.59 (136.53 to 280.33) | 28,614.33 (19,597.95 to 38,927.53) | 282.26 (194.63 to 383.29) | 1.18 (0.95 to 1.41) |
| DALYs | Senegal | 7,776.85 (5,605.00 to 10,933.27) | 398.20 (287.04 to 560.36) | 16,425.38 (10,912.57 to 25,700.44) | 369.45 (245.74 to 579.77) | -0.04 (-0.24 to 0.16) |
| DALYs | Serbia | 2,253.11 (1,782.03 to 2,839.80) | 104.84 (82.89 to 132.12) | 974.73 (773.19 to 1,238.44) | 57.13 (45.22 to 72.75) | -1.91 (-2.38 to -1.44) |
| DALYs | Seychelles | 52.82 (41.82 to 65.89) | 248.44 (196.75 to 309.89) | 70.69 (54.04 to 89.51) | 304.58 (232.35 to 386.31) | 1.69 (1.40 to 1.98) |
| DALYs | Sierra Leone | 2,739.50 (1,698.40 to 4,188.22) | 251.45 (156.02 to 384.42) | 6,926.20 (4,566.56 to 10,642.60) | 268.99 (177.54 to 413.71) | 0.17 (-0.42 to 0.77) |
| DALYs | Singapore | 472.08 (399.17 to 563.44) | 49.55 (41.88 to 59.19) | 330.72 (256.99 to 425.01) | 35.83 (28.05 to 45.98) | -1.09 (-1.81 to -0.36) |
| DALYs | Slovakia | 993.08 (775.82 to 1,271.31) | 82.99 (64.80 to 106.20) | 589.19 (450.70 to 766.39) | 64.84 (49.39 to 84.81) | -0.80 (-1.28 to -0.32) |
| DALYs | Slovenia | 271.92 (219.90 to 339.35) | 60.09 (48.52 to 75.18) | 110.11 (76.94 to 156.36) | 36.82 (25.68 to 52.37) | -1.68 (-2.51 to -0.84) |
| DALYs | Solomon Islands | 239.74 (88.19 to 400.13) | 269.92 (100.03 to 449.31) | 595.16 (381.74 to 825.52) | 331.80 (212.60 to 460.87) | 0.68 (0.22 to 1.13) |
| DALYs | Somalia | 7,865.34 (4,938.63 to 13,011.72) | 403.71 (253.19 to 672.66) | 24,893.63 (15,216.88 to 43,533.73) | 414.26 (253.48 to 722.38) | 0.09 (0.00 to 0.18) |
| DALYs | South Africa | 19,082.41 (16,033.07 to 23,315.08) | 180.77 (151.98 to 220.68) | 22,375.59 (18,792.07 to 27,028.57) | 153.49 (128.86 to 185.50) | -0.55 (-1.27 to 0.17) |
| DALYs | South Sudan | 4,563.44 (3,063.91 to 6,651.40) | 270.75 (182.35 to 394.07) | 11,595.45 (7,400.93 to 17,915.21) | 454.05 (289.47 to 712.24) | 1.73 (0.89 to 2.58) |
| DALYs | Spain | 4,722.96 (3,556.78 to 6,210.74) | 49.20 (37.05 to 64.71) | 2,286.91 (1,531.76 to 3,285.29) | 32.98 (22.11 to 47.33) | -1.29 (-1.80 to -0.78) |
| DALYs | Sri Lanka | 12,052.77 (9,211.20 to 15,559.87) | 250.00 (191.08 to 322.75) | 9,438.98 (6,731.47 to 12,808.08) | 189.74 (135.28 to 257.58) | -0.95 (-2.55 to 0.69) |
| DALYs | Sudan | 7,655.46 (5,081.11 to 12,115.81) | 144.77 (96.22 to 228.98) | 20,099.80 (12,875.95 to 28,680.31) | 160.50 (102.81 to 229.03) | 0.32 (0.23 to 0.42) |
| DALYs | Suriname | 281.10 (198.16 to 354.44) | 245.02 (172.79 to 308.97) | 470.26 (351.28 to 613.02) | 353.39 (263.96 to 460.46) | 1.32 (0.66 to 1.99) |
| DALYs | Sweden | 333.07 (205.35 to 504.60) | 18.25 (11.26 to 27.67) | 433.80 (270.16 to 660.41) | 22.83 (14.23 to 34.84) | 0.73 (0.57 to 0.88) |
| DALYs | Switzerland | 613.41 (422.73 to 866.18) | 37.06 (25.51 to 52.34) | 517.02 (319.48 to 784.67) | 32.65 (20.17 to 49.64) | -0.45 (-0.61 to -0.29) |
| DALYs | Syrian Arab Republic | 10,225.56 (7,267.92 to 13,590.99) | 295.29 (210.99 to 390.99) | 7,966.07 (5,628.65 to 11,847.44) | 237.95 (163.50 to 391.40) | -0.74 (-1.15 to -0.33) |
| DALYs | Taiwan (Province of China) | 8,062.59 (7,165.46 to 9,054.98) | 140.33 (124.78 to 157.61) | 3,300.85 (2,715.21 to 3,962.14) | 76.22 (62.83 to 91.31) | -1.96 (-2.51 to -1.42) |
| DALYs | Tajikistan | 1,327.47 (1,011.00 to 1,755.71) | 89.46 (68.18 to 118.28) | 2,506.24 (1,772.26 to 3,531.34) | 94.42 (66.70 to 133.10) | 0.23 (-0.12 to 0.59) |
| DALYs | Thailand | 44,518.20 (32,634.61 to 63,013.66) | 258.42 (189.61 to 365.53) | 28,753.23 (20,920.82 to 39,525.62) | 226.78 (165.08 to 311.94) | -0.45 (-1.14 to 0.25) |
| DALYs | Timor-Leste | 517.45 (334.04 to 756.68) | 238.86 (154.24 to 349.25) | 992.37 (655.08 to 1,482.13) | 241.76 (159.37 to 361.15) | 0.05 (-1.13 to 1.25) |
| DALYs | Togo | 2,872.51 (2,044.27 to 3,941.88) | 295.73 (211.25 to 405.90) | 6,896.94 (4,582.72 to 9,923.39) | 308.64 (205.26 to 442.88) | 0.16 (-0.07 to 0.39) |
| DALYs | Tokelau | 0.75 (0.49 to 1.21) | 197.64 (127.66 to 317.70) | 1.56 (1.11 to 2.17) | 487.17 (347.69 to 677.46) | 3.26 (2.76 to 3.76) |
| DALYs | Tonga | 25.72 (16.95 to 37.23) | 100.05 (65.89 to 144.90) | 38.16 (26.06 to 53.56) | 148.38 (101.34 to 208.19) | 1.34 (1.11 to 1.57) |
| DALYs | Trinidad and Tobago | 592.51 (517.65 to 684.00) | 179.94 (157.23 to 207.78) | 786.99 (585.04 to 1,012.27) | 288.67 (214.79 to 371.23) | 1.67 (0.78 to 2.57) |
| DALYs | Tunisia | 2,446.07 (1,809.34 to 3,439.31) | 103.53 (76.67 to 145.47) | 2,861.95 (2,040.32 to 3,941.24) | 115.36 (82.20 to 159.03) | 0.34 (0.19 to 0.50) |
| DALYs | Turkey | 28,049.55 (20,807.11 to 37,130.93) | 173.25 (128.58 to 229.53) | 19,946.60 (15,301.15 to 25,754.51) | 103.40 (79.27 to 133.50) | -1.65 (-2.01 to -1.30) |
| DALYs | Turkmenistan | 2,890.94 (2,519.14 to 3,357.42) | 274.93 (239.64 to 319.16) | 6,505.44 (5,046.58 to 8,244.01) | 497.14 (385.54 to 629.83) | 2.07 (1.29 to 2.87) |
| DALYs | Tuvalu | 5.73 (4.03 to 8.52) | 247.52 (174.36 to 368.17) | 10.37 (7.11 to 15.21) | 318.59 (218.71 to 467.21) | 0.84 (0.72 to 0.96) |
| DALYs | Uganda | 9,894.45 (6,448.39 to 14,869.09) | 208.38 (135.77 to 313.06) | 38,903.52 (25,876.56 to 56,505.93) | 311.74 (207.34 to 453.68) | 1.21 (0.87 to 1.55) |
| DALYs | Ukraine | 2,385.97 (1,470.45 to 3,687.17) | 21.35 (13.13 to 33.00) | 4,974.47 (3,688.61 to 6,428.56) | 71.06 (52.73 to 92.01) | 4.07 (3.09 to 5.05) |
| DALYs | United Arab Emirates | 553.69 (385.72 to 780.40) | 109.66 (76.49 to 154.35) | 1,459.79 (846.39 to 1,982.37) | 148.92 (86.83 to 202.04) | 1.16 (0.12 to 2.22) |
| DALYs | United Kingdom | 5,552.31 (3,701.35 to 7,788.61) | 41.15 (27.52 to 57.68) | 5,418.27 (3,564.41 to 7,639.20) | 42.01 (27.66 to 59.27) | 0.05 (-0.25 to 0.35) |
| DALYs | United Republic of Tanzania | 21,576.69 (15,551.75 to 30,530.73) | 307.51 (222.11 to 434.52) | 51,123.25 (35,330.13 to 71,266.21) | 313.75 (216.82 to 437.45) | 0.07 (-0.14 to 0.27) |
| DALYs | United States Virgin Islands | 29,398.05 (22,898.91 to 37,486.26) | 47.20 (36.80 to 60.19) | 43,582.98 (34,502.41 to 54,798.79) | 64.31 (50.88 to 80.95) | 2.55 (1.80 to 3.30) |
| DALYs | United States of America | 39.69 (29.76 to 52.50) | 164.35 (123.34 to 217.63) | 43.50 (28.72 to 68.02) | 325.37 (215.05 to 507.95) | 1.02 (0.78 to 1.27) |
| DALYs | Uruguay | 401.46 (338.54 to 481.00) | 56.01 (47.23 to 67.12) | 506.36 (419.47 to 603.98) | 68.04 (56.36 to 81.15) | 0.59 (0.38 to 0.80) |
| DALYs | Uzbekistan | 8,924.91 (6,834.60 to 11,696.37) | 152.13 (116.51 to 199.34) | 24,876.96 (20,163.88 to 30,456.22) | 299.41 (242.47 to 367.17) | 2.07 (1.46 to 2.68) |
| DALYs | Vanuatu | 69.86 (43.00 to 115.81) | 176.82 (108.82 to 291.83) | 246.61 (165.88 to 381.70) | 295.97 (199.08 to 457.70) | 1.67 (1.48 to 1.87) |
| DALYs | Venezuela (Bolivarian Republic of) | 8,003.13 (6,986.80 to 9,247.26) | 149.77 (130.69 to 173.13) | 14,092.42 (10,808.62 to 17,882.03) | 267.55 (205.40 to 339.17) | 1.89 (1.02 to 2.77) |
| DALYs | Viet Nam | 43,072.08 (24,559.35 to 59,940.09) | 221.80 (126.82 to 308.54) | 41,118.14 (23,670.57 to 56,192.97) | 191.19 (108.97 to 261.18) | -0.49 (-0.59 to -0.40) |
| DALYs | Yemen | 2,131.66 (1,126.83 to 4,169.54) | 70.28 (37.56 to 135.55) | 6,245.91 (3,799.99 to 11,743.35) | 70.90 (43.50 to 131.68) | 0.06 (-0.63 to 0.76) |
| DALYs | Zambia | 9,708.66 (6,769.25 to 13,092.77) | 431.80 (302.58 to 581.42) | 27,020.08 (15,521.72 to 49,491.79) | 478.68 (275.47 to 874.07) | 0.33 (0.18 to 0.49) |
| DALYs | Zimbabwe | 2,826.63 (1,950.12 to 4,592.26) | 102.34 (70.30 to 169.10) | 9,528.36 (6,399.01 to 14,563.03) | 225.29 (150.80 to 347.23) | 2.61 (1.85 to 3.38) |
