# Supplementary figures and images for: Global, regional, and national burden of chronic kidney disease among adolescents and emerging adults from 1990 to 2021
Source: Ren Fail. 2025 May 22;47(1):2508296. doi: 10.1080/0886022X.2025.2508296 (PMC12101043; doi:10.1080/0886022X.2025.2508296)

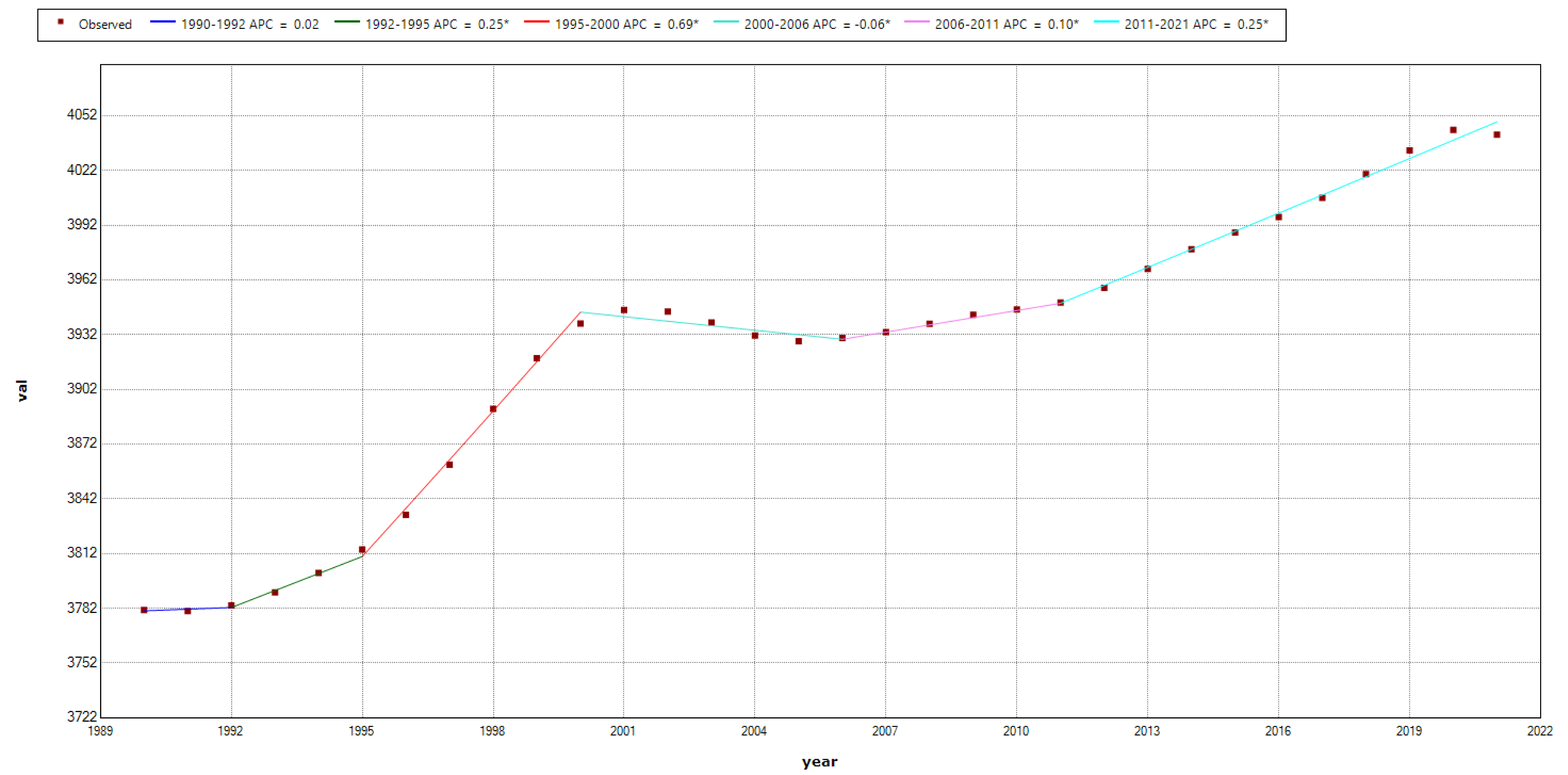

Supplement: S1 B.tif [file IRNF_A_2508296_SM3073.tif]

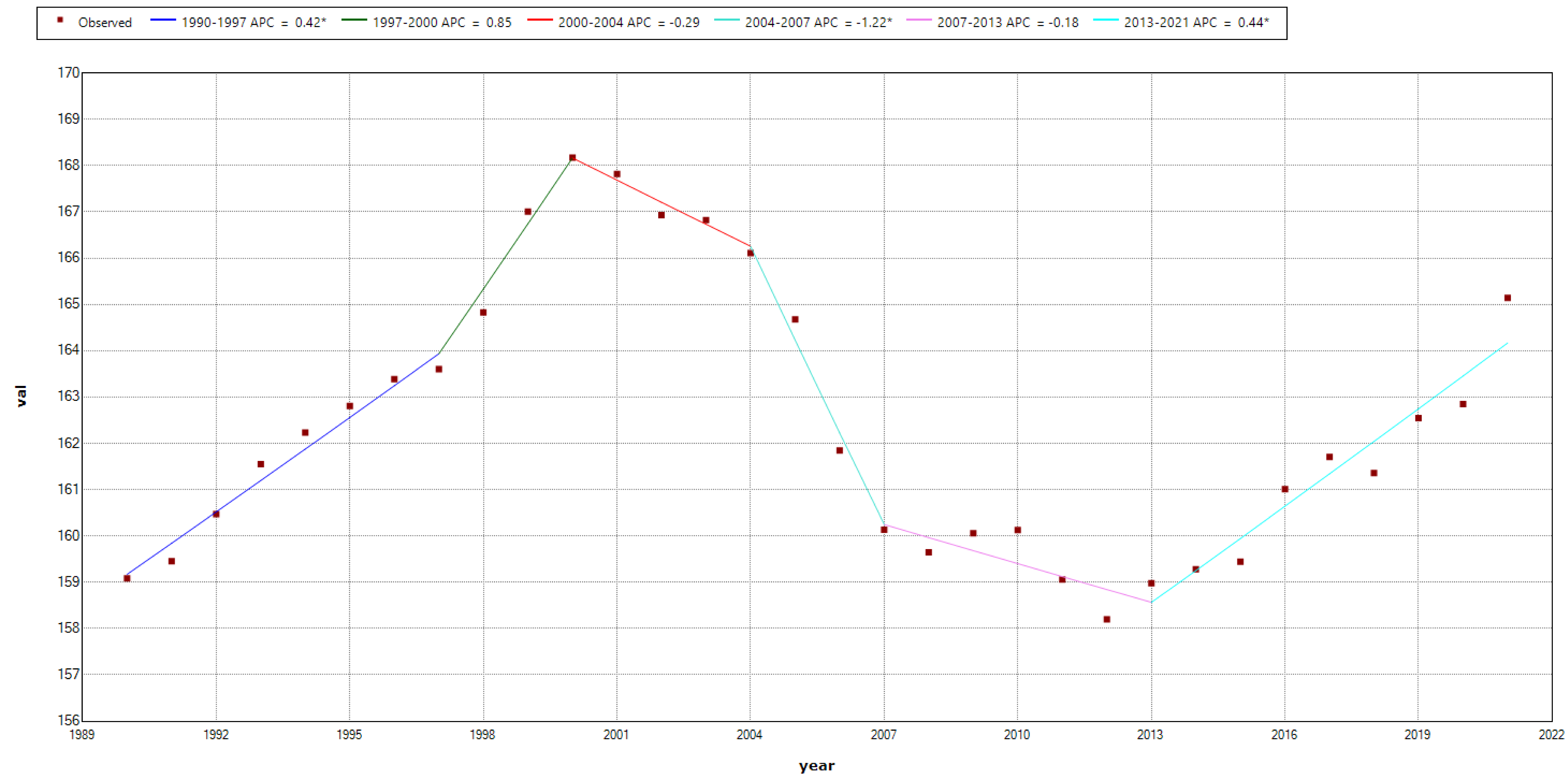

Supplement: S1 D.tif [file IRNF_A_2508296_SM3068.tif]

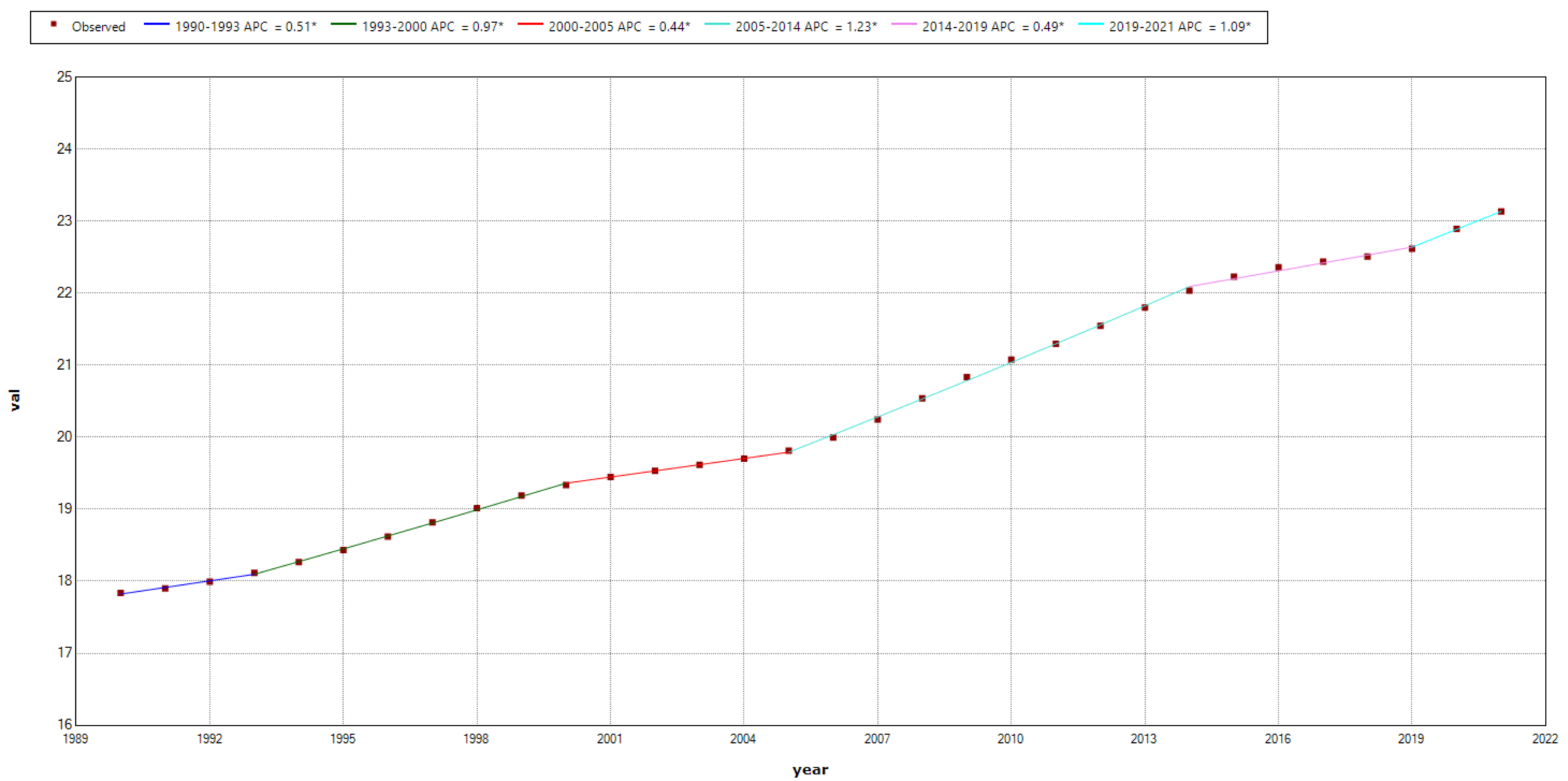

Supplement: S1 A.tif [file IRNF_A_2508296_SM3063.tif]

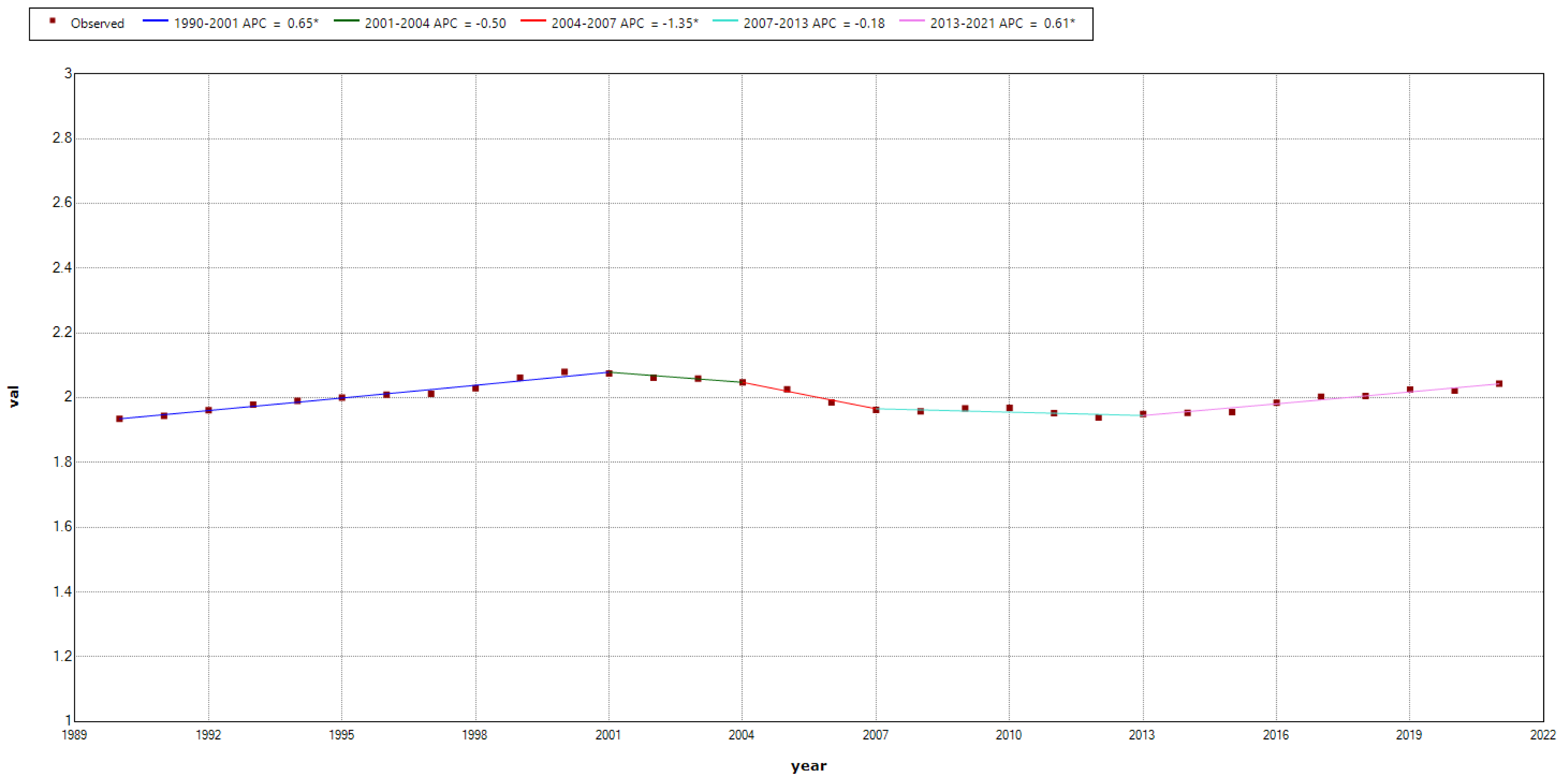

Supplement: S1 C.tif [file IRNF_A_2508296_SM3054.tif]
