# Supplementary material for: Global, regional, and national burden of chronic kidney disease among adolescents and emerging adults from 1990 to 2021
Source: Ren Fail. 2025 May 22;47(1):2508296. doi: 10.1080/0886022X.2025.2508296 (PMC12101043; doi:10.1080/0886022X.2025.2508296)
Supplement: Supplementary Table S5.docx [file IRNF_A_2508296_SM3062.docx]

Supplementary Table S5: Detailed Data for Frontier Analysis.

| Location | SDI | Rate of DALYs (95% UI) | Frontier DALYs | Effective difference | Effective difference rank (Age-standardized DALYs rank) | SDI region |
| --- | --- | --- | --- | --- | --- | --- |
| Afghanistan | 0.337199997837472 | 286.59 (170.55 to 468.28) | 71.13 | 215.46 | 134 (137) | Low SDI |
| Albania | 0.706849790878611 | 69.80 (49.45 to 100.13) | 21.6 | 48.2 | 54 (47) | Middle SDI |
| Algeria | 0.659500924324217 | 128.74 (93.98 to 167.61) | 21.5 | 107.24 | 88 (80) | Middle SDI |
| American Samoa | 0.723727533059592 | 519.08 (362.70 to 703.78) | 22.05 | 497.03 | 201 (201) | High-middle SDI |
| Andorra | 0.869444112735696 | 34.57 (22.59 to 50.05) | 16.52 | 18.05 | 17 (15) | High SDI |
| Angola | 0.453721949492174 | 351.45 (234.60 to 517.15) | 67.25 | 284.21 | 162 (165) | Low SDI |
| Antigua and Barbuda | 0.749886887369379 | 247.86 (212.58 to 286.59) | 21.72 | 226.14 | 137 (124) | High-middle SDI |
| Argentina | 0.723122972582249 | 90.75 (78.59 to 105.41) | 21.7 | 69.06 | 66 (57) | High-middle SDI |
| Armenia | 0.701833194147139 | 125.87 (98.97 to 160.81) | 21.75 | 104.11 | 86 (78) | Middle SDI |
| Australia | 0.844252813727575 | 23.94 (17.60 to 32.77) | 16.52 | 7.42 | 6 (5) | High SDI |
| Austria | 0.853837004256539 | 36.69 (24.98 to 51.19) | 16.52 | 20.17 | 26 (21) | High SDI |
| Azerbaijan | 0.694851274415291 | 218.02 (164.18 to 288.14) | 21.74 | 196.28 | 126 (113) | Middle SDI |
| Bahamas | 0.805020667882124 | 303.42 (237.72 to 385.40) | 17.22 | 286.2 | 163 (147) | High-middle SDI |
| Bahrain | 0.753043204117567 | 142.86 (108.98 to 189.72) | 21.74 | 121.11 | 93 (85) | High-middle SDI |
| Bangladesh | 0.492420884955353 | 93.65 (68.04 to 133.28) | 67.2 | 26.45 | 35 (60) | Low-middle SDI |
| Barbados | 0.746748764251474 | 179.99 (139.05 to 230.36) | 22.44 | 157.55 | 106 (100) | High-middle SDI |
| Belarus | 0.784484710906639 | 37.25 (26.93 to 51.20) | 18.43 | 18.82 | 21 (23) | High-middle SDI |
| Belgium | 0.853654015777112 | 37.54 (24.44 to 54.56) | 16.52 | 21.01 | 28 (24) | High SDI |
| Belize | 0.610229002222948 | 355.72 (302.07 to 423.25) | 58.65 | 297.08 | 166 (167) | Low-middle SDI |
| Benin | 0.373486574239249 | 334.77 (226.69 to 473.81) | 69.85 | 264.92 | 153 (162) | Low SDI |
| Bermuda | 0.821365422389484 | 93.89 (76.64 to 115.39) | 16.52 | 77.37 | 71 (61) | High SDI |
| Bhutan | 0.473062378179926 | 137.64 (92.35 to 198.77) | 67.11 | 70.53 | 68 (84) | Low-middle SDI |
| Bolivia (Plurinational State of) | 0.5990107988623 | 213.88 (141.92 to 317.74) | 56.23 | 157.66 | 107 (109) | Low-middle SDI |
| Bosnia and Herzegovina | 0.72307789321024 | 71.12 (52.70 to 94.30) | 22.37 | 48.75 | 56 (50) | High-middle SDI |
| Botswana | 0.642721628921122 | 125.62 (81.60 to 209.28) | 39.44 | 86.19 | 77 (77) | Middle SDI |
| Brazil | 0.65304388724724 | 87.32 (76.14 to 102.42) | 35.83 | 51.49 | 58 (56) | Middle SDI |
| Brunei Darussalam | 0.810234366947283 | 101.11 (76.75 to 131.63) | 17.35 | 83.76 | 75 (66) | High-middle SDI |
| Bulgaria | 0.768150939342518 | 125.48 (96.96 to 162.40) | 20.84 | 104.64 | 87 (76) | High-middle SDI |
| Burkina Faso | 0.285118401552141 | 369.43 (255.58 to 524.94) | 71.78 | 297.65 | 168 (174) | Low SDI |
| Burundi | 0.289374364823265 | 298.11 (199.25 to 465.07) | 69.86 | 228.26 | 139 (143) | Low SDI |
| Cabo Verde | 0.53353453903661 | 187.26 (125.99 to 265.14) | 69.19 | 118.07 | 91 (102) | Low-middle SDI |
| Cambodia | 0.473621490708013 | 281.20 (178.90 to 430.87) | 67.51 | 213.69 | 133 (134) | Low-middle SDI |
| Cameroon | 0.479691223419746 | 468.13 (302.43 to 695.95) | 67.24 | 400.89 | 190 (192) | Low-middle SDI |
| Canada | 0.873170679735405 | 43.22 (32.71 to 57.34) | 16.52 | 26.7 | 37 (32) | High SDI |
| Central African Republic | 0.309167689992842 | 461.04 (303.30 to 725.36) | 71.57 | 389.47 | 186 (188) | Low SDI |
| Chad | 0.240436018662427 | 288.23 (192.90 to 447.29) | 102.37 | 185.86 | 121 (138) | Low SDI |
| Chile | 0.771514715903929 | 60.25 (49.50 to 72.69) | 19.61 | 40.64 | 48 (41) | High-middle SDI |
| China | 0.721629759719777 | 76.10 (60.44 to 93.47) | 22.6 | 53.5 | 59 (52) | High-middle SDI |
| Colombia | 0.655442912568827 | 92.44 (75.16 to 112.82) | 37.21 | 55.22 | 61 (58) | Middle SDI |
| Comoros | 0.475978688247514 | 339.32 (233.22 to 485.16) | 67.44 | 271.88 | 158 (163) | Low-middle SDI |
| Congo | 0.583075236262154 | 461.44 (306.96 to 657.78) | 63.13 | 398.31 | 189 (189) | Low-middle SDI |
| Cook Islands | 0.779109954659683 | 129.33 (92.90 to 177.49) | 18.6 | 110.72 | 89 (81) | High-middle SDI |
| Costa Rica | 0.700340476999325 | 150.92 (127.69 to 179.74) | 22.27 | 128.65 | 96 (91) | Middle SDI |
| Coted'Ivoire | 0.425941883067361 | 365.20 (238.57 to 525.09) | 69.79 | 295.4 | 165 (170) | Low SDI |
| Croatia | 0.798341026936276 | 52.47 (39.65 to 70.85) | 17.82 | 34.65 | 44 (38) | High-middle SDI |
| Cuba | 0.668729864363244 | 92.94 (76.29 to 112.89) | 22.72 | 70.22 | 67 (59) | Middle SDI |
| Cyprus | 0.835630545002355 | 36.09 (23.12 to 51.76) | 16.52 | 19.57 | 25 (20) | High SDI |
| Czechia | 0.828450433122785 | 45.32 (33.11 to 61.61) | 16.52 | 28.8 | 40 (33) | High SDI |
| Democratic People's Republic of Korea | 0.569854634067777 | 148.38 (104.96 to 212.28) | 64.98 | 83.39 | 74 (87) | Low-middle SDI |
| Democratic Republic of the Congo | 0.38317984920136 | 358.92 (232.39 to 517.77) | 71.12 | 287.79 | 164 (168) | Low SDI |
| Denmark | 0.896424204101212 | 33.48 (20.25 to 51.28) | 16.52 | 16.96 | 14 (13) | High SDI |
| Djibouti | 0.4879583707169 | 281.87 (178.77 to 439.10) | 68.37 | 213.5 | 132 (135) | Low-middle SDI |
| Dominica | 0.746967184749286 | 332.12 (240.46 to 444.49) | 21.63 | 310.49 | 171 (161) | High-middle SDI |
| Dominican Republic | 0.619388201281128 | 234.26 (159.24 to 307.41) | 56.15 | 178.11 | 119 (120) | Middle SDI |
| Ecuador | 0.66101705262103 | 161.95 (128.28 to 205.04) | 26.98 | 134.98 | 100 (96) | Middle SDI |
| Egypt | 0.6067870937016 | 224.51 (173.05 to 284.20) | 59.58 | 164.92 | 110 (115) | Low-middle SDI |
| El Salvador | 0.563775187564695 | 451.71 (323.21 to 605.75) | 63.07 | 388.64 | 185 (185) | Low-middle SDI |
| Equatorial Guinea | 0.657857455522296 | 435.44 (238.14 to 728.70) | 23.89 | 411.55 | 193 (184) | Middle SDI |
| Eritrea | 0.403863942828898 | 319.83 (194.66 to 520.89) | 69.81 | 250.02 | 145 (155) | Low SDI |
| Estonia | 0.844917787360624 | 135.50 (110.67 to 174.47) | 16.52 | 118.98 | 92 (82) | High SDI |
| Eswatini | 0.58545971329224 | 279.71 (162.63 to 427.77) | 63.37 | 216.34 | 135 (133) | Low-middle SDI |
| Ethiopia | 0.358823295416803 | 298.24 (231.75 to 373.91) | 71.12 | 227.12 | 138 (144) | Low SDI |
| Fiji | 0.675051630810876 | 365.48 (260.66 to 495.98) | 22.28 | 343.2 | 179 (171) | Middle SDI |
| Finland | 0.859831367662218 | 25.43 (15.26 to 38.86) | 16.52 | 8.92 | 7 (6) | High SDI |
| France | 0.838364875196954 | 22.12 (15.53 to 31.50) | 16.52 | 5.59 | 2 (1) | High SDI |
| Gabon | 0.634691392755259 | 488.07 (264.10 to 754.58) | 39.8 | 448.27 | 197 (198) | Middle SDI |
| Gambia | 0.40971415951073 | 423.57 (277.05 to 619.84) | 69.77 | 353.81 | 182 (182) | Low SDI |
| Georgia | 0.732473604476306 | 215.57 (178.33 to 265.95) | 22.39 | 193.19 | 124 (110) | High-middle SDI |
| Germany | 0.902957091057572 | 33.53 (22.15 to 47.66) | 16.52 | 17.01 | 15 (14) | High SDI |
| Ghana | 0.564930389658009 | 457.00 (304.20 to 666.16) | 63.94 | 393.06 | 187 (187) | Low-middle SDI |
| Greece | 0.791854407912829 | 47.19 (34.36 to 62.39) | 18.49 | 28.7 | 39 (35) | High-middle SDI |
| Greenland | 0.82621033634361 | 35.33 (24.90 to 48.40) | 16.52 | 18.81 | 20 (16) | High SDI |
| Grenada | 0.668993028326993 | 343.83 (285.64 to 414.45) | 22.59 | 321.24 | 175 (164) | Middle SDI |
| Guam | 0.803982202863145 | 193.78 (149.46 to 247.64) | 17.15 | 176.63 | 117 (105) | High-middle SDI |
| Guatemala | 0.539972423611408 | 378.23 (319.64 to 443.77) | 67.6 | 310.63 | 172 (176) | Low-middle SDI |
| Guinea | 0.33640129335069 | 326.31 (224.11 to 486.52) | 71.12 | 255.19 | 149 (158) | Low SDI |
| Guinea-Bissau | 0.353109621364536 | 488.05 (332.69 to 695.29) | 71.16 | 416.89 | 194 (197) | Low SDI |
| Guyana | 0.650812335285137 | 366.18 (260.50 to 491.38) | 36.86 | 329.32 | 176 (173) | Middle SDI |
| Haiti | 0.448278284992742 | 257.26 (144.75 to 540.57) | 67.56 | 189.7 | 122 (127) | Low SDI |
| Honduras | 0.513037248307344 | 97.29 (61.73 to 145.55) | 67.82 | 29.46 | 41 (63) | Low-middle SDI |
| Hungary | 0.790754768173338 | 46.13 (34.10 to 63.09) | 18.58 | 27.55 | 38 (34) | High-middle SDI |
| Iceland | 0.876361679802766 | 23.23 (14.97 to 34.32) | 16.52 | 6.72 | 4 (3) | High SDI |
| India | 0.575401649396992 | 102.85 (83.10 to 130.05) | 63.24 | 39.61 | 47 (68) | Low-middle SDI |
| Indonesia | 0.656868336261681 | 303.31 (217.43 to 395.04) | 35.89 | 267.42 | 156 (146) | Middle SDI |
| Iran (Islamic Republic of) | 0.697207397661899 | 98.46 (82.48 to 117.19) | 22.52 | 75.94 | 70 (65) | Middle SDI |
| Iraq | 0.662626230762701 | 136.39 (83.95 to 197.47) | 24.77 | 111.62 | 90 (83) | Middle SDI |
| Ireland | 0.873753849787408 | 40.01 (24.79 to 59.67) | 16.52 | 23.49 | 31 (27) | High SDI |
| Israel | 0.809011651557631 | 50.84 (36.54 to 68.05) | 16.82 | 34.02 | 43 (37) | High-middle SDI |
| Italy | 0.805773533613512 | 40.99 (25.88 to 60.45) | 17.6 | 23.38 | 30 (29) | High-middle SDI |
| Jamaica | 0.683263063531091 | 180.03 (131.22 to 238.04) | 21.98 | 158.06 | 109 (101) | Middle SDI |
| Japan | 0.871241812572128 | 27.69 (21.86 to 35.10) | 16.52 | 11.18 | 10 (9) | High SDI |
| Jordan | 0.725307226832443 | 123.39 (93.93 to 157.88) | 21.52 | 101.86 | 84 (75) | High-middle SDI |
| Kazakhstan | 0.725144495100349 | 144.26 (111.62 to 190.34) | 21.78 | 122.48 | 94 (86) | High-middle SDI |
| Kenya | 0.523768077100117 | 252.64 (196.09 to 344.29) | 67.8 | 184.84 | 120 (125) | Low-middle SDI |
| Kiribati | 0.527186582842681 | 412.56 (258.09 to 649.74) | 67.39 | 345.17 | 181 (180) | Low-middle SDI |
| Kuwait | 0.846651054742869 | 77.08 (60.37 to 97.67) | 16.52 | 60.56 | 64 (54) | High SDI |
| Kyrgyzstan | 0.603979328412055 | 223.43 (184.91 to 272.06) | 58.15 | 165.29 | 112 (114) | Low-middle SDI |
| Lao People's Democratic Republic | 0.489136091456599 | 382.26 (252.48 to 583.71) | 68.08 | 314.18 | 173 (178) | Low-middle SDI |
| Latvia | 0.830663516299797 | 71.58 (56.56 to 92.72) | 16.52 | 55.06 | 60 (51) | High SDI |
| Lebanon | 0.74474635096338 | 111.28 (84.02 to 143.81) | 21.97 | 89.3 | 78 (71) | High-middle SDI |
| Lesotho | 0.510393065681381 | 216.49 (143.26 to 315.62) | 68.29 | 148.2 | 103 (111) | Low-middle SDI |
| Liberia | 0.352442451643965 | 466.54 (314.25 to 667.32) | 69.81 | 396.72 | 188 (191) | Low SDI |
| Libya | 0.725771398888229 | 199.81 (129.96 to 277.46) | 22.1 | 177.72 | 118 (107) | High-middle SDI |
| Lithuania | 0.856484049109405 | 64.21 (48.94 to 87.63) | 16.52 | 47.69 | 52 (42) | High SDI |
| Luxembourg | 0.884428955125693 | 35.36 (22.39 to 52.19) | 16.52 | 18.84 | 22 (17) | High SDI |
| Madagascar | 0.40024694263784 | 268.14 (182.89 to 378.74) | 69.81 | 198.33 | 128 (129) | Low SDI |
| Malawi | 0.384553633730301 | 426.20 (291.92 to 597.51) | 71.12 | 355.08 | 183 (183) | Low SDI |
| Malaysia | 0.742523828414751 | 160.75 (121.41 to 206.53) | 22.08 | 138.67 | 101 (94) | High-middle SDI |
| Maldives | 0.650886627343107 | 176.03 (129.27 to 227.87) | 36.66 | 139.37 | 102 (98) | Middle SDI |
| Mali | 0.268579940925684 | 323.39 (229.69 to 455.84) | 71.13 | 252.26 | 146 (156) | Low SDI |
| Malta | 0.801585033998971 | 40.98 (27.27 to 58.83) | 17.17 | 23.81 | 32 (28) | High-middle SDI |
| Marshall Islands | 0.574091128177309 | 487.28 (174.75 to 1,366.39) | 63.27 | 424 | 196 (195) | Low-middle SDI |
| Mauritania | 0.498945100442996 | 272.64 (176.55 to 401.42) | 67.37 | 205.27 | 130 (131) | Low-middle SDI |
| Mauritius | 0.718260445552653 | 578.67 (498.64 to 657.72) | 21.69 | 556.98 | 203 (203) | High-middle SDI |
| Mexico | 0.664575304259183 | 361.43 (320.00 to 415.10) | 24.78 | 336.65 | 177 (169) | Middle SDI |
| Micronesia (Federated States of) | 0.587534967103163 | 464.52 (309.43 to 680.73) | 56.79 | 407.73 | 191 (190) | Low-middle SDI |
| Monaco | 0.908262830989574 | 38.83 (26.33 to 53.09) | 16.52 | 22.32 | 29 (25) | High SDI |
| Mongolia | 0.617621564638127 | 224.99 (173.94 to 290.60) | 56.21 | 168.78 | 114 (116) | Low-middle SDI |
| Montenegro | 0.795800584021403 | 97.31 (72.35 to 126.80) | 17.4 | 79.91 | 72 (64) | High-middle SDI |
| Morocco | 0.562698300927195 | 125.97 (88.94 to 187.69) | 62.47 | 63.5 | 65 (79) | Low-middle SDI |
| Mozambique | 0.326462613636377 | 408.47 (267.73 to 609.64) | 69.82 | 338.65 | 178 (179) | Low SDI |
| Myanmar | 0.533900839611906 | 365.78 (251.86 to 501.59) | 68.17 | 297.61 | 167 (172) | Low-middle SDI |
| Namibia | 0.617564872149664 | 103.86 (65.73 to 167.42) | 56.81 | 47.05 | 51 (70) | Low-middle SDI |
| Nauru | 0.625177833565553 | 505.84 (342.80 to 709.37) | 46.13 | 459.72 | 198 (200) | Middle SDI |
| Nepal | 0.43317463481343 | 164.14 (114.09 to 233.01) | 69.76 | 94.38 | 82 (97) | Low SDI |
| Netherlands | 0.888464256397732 | 36.07 (22.14 to 53.31) | 16.52 | 19.55 | 24 (19) | High SDI |
| New Zealand | 0.849442498970719 | 33.13 (25.38 to 43.49) | 16.53 | 16.6 | 13 (12) | High SDI |
| Nicaragua | 0.523958472235925 | 329.34 (249.73 to 421.08) | 67.17 | 262.17 | 152 (159) | Low-middle SDI |
| Niger | 0.168072773803995 | 217.90 (136.89 to 374.40) | 199.81 | 18.1 | 18 (112) | Low SDI |
| Nigeria | 0.503390832763244 | 241.26 (152.00 to 362.03) | 67.42 | 173.84 | 115 (122) | Low-middle SDI |
| Niue | 0.726222049709539 | 592.72 (346.32 to 962.40) | 22.09 | 570.63 | 204 (204) | High-middle SDI |
| North Macedonia | 0.750629703270047 | 78.75 (55.38 to 112.92) | 21.89 | 56.86 | 62 (55) | High-middle SDI |
| Northern Mariana Islands | 0.771535213383281 | 278.97 (207.11 to 357.84) | 20.13 | 258.84 | 150 (132) | High-middle SDI |
| Norway | 0.916132810119013 | 27.57 (17.11 to 40.42) | 16.52 | 11.05 | 9 (8) | High SDI |
| Oman | 0.773391601871737 | 123.03 (91.06 to 165.44) | 19.06 | 103.97 | 85 (74) | High-middle SDI |
| Pakistan | 0.504028688613448 | 232.20 (172.05 to 300.59) | 67.17 | 165.03 | 111 (119) | Low-middle SDI |
| Palau | 0.75404693141209 | 557.83 (306.16 to 824.13) | 22.56 | 535.27 | 202 (202) | High-middle SDI |
| Palestine | 0.631011665054344 | 148.56 (115.05 to 193.38) | 53.36 | 95.2 | 83 (89) | Middle SDI |
| Panama | 0.708864827518009 | 179.82 (145.47 to 221.66) | 22.39 | 157.43 | 105 (99) | Middle SDI |
| Papua New Guinea | 0.417797443084353 | 194.69 (130.74 to 270.89) | 69.76 | 124.93 | 95 (106) | Low SDI |
| Paraguay | 0.635718098852038 | 112.86 (83.96 to 148.40) | 39.49 | 73.38 | 69 (72) | Middle SDI |
| Peru | 0.662054036755862 | 161.02 (116.12 to 216.86) | 30.63 | 130.39 | 97 (95) | Middle SDI |
| Philippines | 0.651219328702707 | 381.38 (308.05 to 442.54) | 37.45 | 343.92 | 180 (177) | Middle SDI |
| Poland | 0.812042808667712 | 42.00 (30.34 to 58.80) | 16.64 | 25.37 | 33 (30) | High SDI |
| Portugal | 0.744151850665712 | 39.07 (28.73 to 53.52) | 21.81 | 17.26 | 16 (26) | High-middle SDI |
| Puerto Rico | 0.825525846914029 | 209.38 (173.08 to 249.51) | 16.52 | 192.86 | 123 (108) | High SDI |
| Qatar | 0.846860584038945 | 101.96 (74.00 to 141.23) | 16.52 | 85.44 | 76 (67) | High SDI |
| Republic of Korea | 0.886675266756997 | 23.37 (17.98 to 30.51) | 16.52 | 6.85 | 5 (4) | High SDI |
| Republic of Moldova | 0.732214875427507 | 67.32 (48.48 to 92.57) | 22.57 | 44.75 | 49 (45) | High-middle SDI |
| Romania | 0.768453864365965 | 58.45 (44.35 to 77.06) | 21.97 | 36.48 | 45 (40) | High-middle SDI |
| Russian Federation | 0.808536004906566 | 50.81 (40.60 to 64.36) | 17.18 | 33.63 | 42 (36) | High-middle SDI |
| Rwanda | 0.435588705789772 | 289.04 (191.73 to 429.54) | 69.81 | 219.24 | 136 (140) | Low SDI |
| Saint Kitts and Nevis | 0.754987054945241 | 254.49 (197.19 to 338.81) | 22.08 | 232.41 | 141 (126) | High-middle SDI |
| Saint Lucia | 0.672509735365292 | 289.54 (228.49 to 352.49) | 22.15 | 267.39 | 155 (141) | Middle SDI |
| Saint Vincent and the Grenadines | 0.637195962512539 | 317.72 (263.29 to 380.46) | 39.95 | 277.77 | 160 (153) | Middle SDI |
| Samoa | 0.593392769469481 | 309.84 (208.68 to 434.12) | 56.48 | 253.36 | 147 (150) | Low-middle SDI |
| San Marino | 0.888005473958106 | 27.46 (16.70 to 41.20) | 16.52 | 10.95 | 8 (7) | High SDI |
| Sao Tome and Principe | 0.505413746708186 | 487.38 (287.62 to 738.07) | 67.79 | 419.58 | 195 (196) | Low-middle SDI |
| Saudi Arabia | 0.815143493214829 | 282.26 (194.63 to 383.29) | 16.64 | 265.62 | 154 (136) | High SDI |
| Senegal | 0.408054192634391 | 369.45 (245.74 to 579.77) | 69.81 | 299.65 | 169 (175) | Low SDI |
| Serbia | 0.792416293685483 | 57.13 (45.22 to 72.75) | 18.48 | 38.65 | 46 (39) | High-middle SDI |
| Seychelles | 0.730150774629382 | 304.58 (232.35 to 386.31) | 22.27 | 282.31 | 161 (148) | High-middle SDI |
| Sierra Leone | 0.3586658807038 | 268.99 (177.54 to 413.71) | 71.63 | 197.36 | 127 (130) | Low SDI |
| Singapore | 0.856097766112729 | 35.83 (28.05 to 45.98) | 16.52 | 19.31 | 23 (18) | High SDI |
| Slovakia | 0.810610529921693 | 64.84 (49.39 to 84.81) | 16.56 | 48.28 | 55 (44) | High SDI |
| Slovenia | 0.842430731281465 | 36.82 (25.68 to 52.37) | 16.52 | 20.3 | 27 (22) | High SDI |
| Solomon Islands | 0.429360316406646 | 331.80 (212.60 to 460.87) | 69.78 | 262.01 | 151 (160) | Low SDI |
| Somalia | 0.0776881089723748 | 414.26 (253.48 to 722.38) | 395.66 | 18.6 | 19 (181) | Low SDI |
| South Africa | 0.679626598156588 | 153.49 (128.86 to 185.50) | 21.49 | 132.01 | 98 (92) | Middle SDI |
| South Sudan | 0.278371125484101 | 454.05 (289.47 to 712.24) | 71.78 | 382.27 | 184 (186) | Low SDI |
| Spain | 0.769283697979998 | 32.98 (22.11 to 47.33) | 20.11 | 12.87 | 11 (11) | High-middle SDI |
| Sri Lanka | 0.70153493519423 | 189.74 (135.28 to 257.58) | 22.27 | 167.47 | 113 (103) | Middle SDI |
| Sudan | 0.54194973514933 | 160.50 (102.81 to 229.03) | 69.35 | 91.15 | 79 (93) | Low-middle SDI |
| Suriname | 0.63366573910901 | 353.39 (263.96 to 460.46) | 37.44 | 315.95 | 174 (166) | Middle SDI |
| Sweden | 0.886880298870342 | 22.83 (14.23 to 34.84) | 16.52 | 6.31 | 3 (2) | High SDI |
| Switzerland | 0.933059110986638 | 32.65 (20.17 to 49.64) | 16.52 | 16.13 | 12 (10) | High SDI |
| Syrian Arab Republic | 0.62300407463119 | 237.95 (163.50 to 391.40) | 42.81 | 195.14 | 125 (121) | Middle SDI |
| Taiwan (Province of China) | 0.874747053187929 | 76.22 (62.83 to 91.31) | 16.52 | 59.7 | 63 (53) | High SDI |
| Tajikistan | 0.541511187051998 | 94.42 (66.70 to 133.10) | 67.96 | 26.46 | 36 (62) | Low-middle SDI |
| Thailand | 0.682547932974593 | 226.78 (165.08 to 311.94) | 22.4 | 204.38 | 129 (118) | Middle SDI |
| Timor-Leste | 0.444667618881833 | 241.76 (159.37 to 361.15) | 67.49 | 174.26 | 116 (123) | Low SDI |
| Togo | 0.408533695477986 | 308.64 (205.26 to 442.88) | 69.82 | 238.82 | 142 (149) | Low SDI |
| Tokelau | 0.68642562083005 | 487.17 (347.69 to 677.46) | 22.08 | 465.09 | 199 (194) | Middle SDI |
| Tonga | 0.626349935807987 | 148.38 (101.34 to 208.19) | 55.97 | 92.41 | 80 (88) | Middle SDI |
| Trinidad and Tobago | 0.768763253980677 | 288.67 (214.79 to 371.23) | 20.71 | 267.96 | 157 (139) | High-middle SDI |
| Tunisia | 0.682432215876962 | 115.36 (82.20 to 159.03) | 22.62 | 92.74 | 81 (73) | Middle SDI |
| Turkey | 0.712692673098478 | 103.40 (79.27 to 133.50) | 21.55 | 81.85 | 73 (69) | High-middle SDI |
| Turkmenistan | 0.682160775869611 | 497.14 (385.54 to 629.83) | 22.1 | 475.04 | 200 (199) | Middle SDI |
| Tuvalu | 0.576620529344684 | 318.59 (218.71 to 467.21) | 64.47 | 254.13 | 148 (154) | Low-middle SDI |
| Uganda | 0.423261181495743 | 311.74 (207.34 to 453.68) | 69.78 | 241.96 | 143 (151) | Low SDI |
| Ukraine | 0.760773912846782 | 71.06 (52.73 to 92.01) | 22.2 | 48.86 | 57 (49) | High-middle SDI |
| United Arab Emirates | 0.849317734035032 | 148.92 (86.83 to 202.04) | 16.52 | 132.4 | 99 (90) | High SDI |
| United Kingdom | 0.859000181772086 | 42.01 (27.66 to 59.27) | 16.53 | 25.48 | 34 (31) | High SDI |
| United Republic of Tanzania | 0.446568273334685 | 313.75 (216.82 to 437.45) | 67.23 | 246.52 | 144 (152) | Low SDI |
| United States of America | 0.862448354015145 | 64.31 (50.88 to 80.95) | 16.52 | 47.79 | 53 (43) | High SDI |
| United States Virgin Islands | 0.821830853383701 | 325.37 (215.05 to 507.95) | 16.52 | 308.85 | 170 (157) | High SDI |
| Uruguay | 0.719283444839432 | 68.04 (56.36 to 81.15) | 21.71 | 46.33 | 50 (46) | High-middle SDI |
| Uzbekistan | 0.662621694181816 | 299.41 (242.47 to 367.17) | 22 | 277.41 | 159 (145) | Middle SDI |
| Vanuatu | 0.473100705820327 | 295.97 (199.08 to 457.70) | 67.21 | 228.76 | 140 (142) | Low-middle SDI |
| Venezuela (Bolivarian Republic of) | 0.596513058857335 | 267.55 (205.40 to 339.17) | 60.95 | 206.6 | 131 (128) | Low-middle SDI |
| Viet Nam | 0.627933720680671 | 191.19 (108.97 to 261.18) | 42.01 | 149.18 | 104 (104) | Middle SDI |
| Yemen | 0.450376375244811 | 70.90 (43.50 to 131.68) | 67.1 | 3.8 | 1 (48) | Low SDI |
| Zambia | 0.505948954434909 | 478.68 (275.47 to 874.07) | 67.6 | 411.08 | 192 (193) | Low-middle SDI |
| Zimbabwe | 0.473819486202707 | 225.29 (150.80 to 347.23) | 67.48 | 157.81 | 108 (117) | Low-middle SDI |
