# Supplementary material for: Global, regional, and national burden of chronic kidney disease among adolescents and emerging adults from 1990 to 2021
Source: Ren Fail. 2025 May 22;47(1):2508296. doi: 10.1080/0886022X.2025.2508296 (PMC12101043; doi:10.1080/0886022X.2025.2508296)
Supplement: Supplementary Table S1.docx [file IRNF_A_2508296_SM3057.docx]

Supplementary Table S1 Global Prevalence of CKD and Their AAPCs From 1990 to 2021 by Sex, Age, Cause, SDI, Region and Country in Adolescents and Emerging adults

| **Measure** | **Variable** | 1990 | | 2021 | | AAPC (95% UI) |
| --- | --- | --- | --- | --- | --- | --- |
|  |  | Number (95% UI) | ASR (95% UI) | Number (95% UI) | ASR (95% UI) |  |
| Prevalence | Global | 54,307,570.36 (41,928,466.44 to 69,066,851.39) | 3,781.04 (2,918.77 to 4,806.54) | 73,517,242.68 (56,149,826.55 to 94,211,056.76) | 4,041.58 (3,086.92 to 5,179.46) | 0.22 (0.19 to 0.25) |
|  | **Sex** | | | | | |
| Prevalence | Male | 27,091,376.16 (20,843,332.50 to 34,488,251.51) | 3,741.34 (2,877.82 to 4,760.65) | 37,346,627.10 (28,474,290.48 to 47,907,790.65) | 4,044.74 (3,083.88 to 5,188.72) | 0.27 (0.24 to 0.30) |
| Prevalence | Female | 27,216,194.20 (21,041,201.17 to 34,636,739.61) | 3,821.32 (2,954.11 to 4,861.22) | 36,170,615.58 (27,626,126.85 to 46,257,712.74) | 4,038.49 (3,084.69 to 5,164.99) | 0.18 (0.16 to 0.20) |
|  | **Age** | | | | | |
| Prevalence | 15-19 | 9,348,569.05 (7,309,255.33 to 11,705,929.49) | 1,799.80 (1,407.19 to 2,253.64) | 11,724,707.85 (9,043,291.56 to 14,878,161.75) | 1,879.02 (1,449.29 to 2,384.40) | 0.14 (0.12 to 0.16) |
| Prevalence | 20-24 | 19,162,023.12 (14,782,374.13 to 24,917,274.23) | 3,894.02 (3,004.01 to 5,063.58) | 25,188,821.51 (19,182,883.25 to 32,987,496.76) | 4,218.12 (3,212.36 to 5,524.08) | 0.26 (0.25 to 0.28) |
| Prevalence | 25-29 | 25,796,978.19 (19,836,836.98 to 32,443,647.67) | 5,828.25 (4,481.69 to 7,329.92) | 36,603,713.33 (27,923,651.74 to 46,345,398.25) | 6,221.49 (4,746.15 to 7,877.27) | 0.22 (0.20 to 0.25) |
|  | **Cause** | | | | | |
| Prevalence | Diabetes mellitus type 1 | 983,776.89 (717,892.86 to 1,313,220.35) | 67.95 (49.57 to 90.61) | 1,971,632.74 (1,419,252.98 to 2,662,657.65) | 108.79 (78.32 to 146.95) | 1.54 (1.44 to 1.64) |
| Prevalence | Diabetes mellitus type 2 | 7,897,833.32 (5,670,514.29 to 10,636,829.82) | 552.05 (396.36 to 743.77) | 7,298,032.07 (5,335,848.38 to 9,728,872.25) | 400.40 (292.73 to 533.84) | -1.03 (-1.08 to -0.98) |
| Prevalence | Hypertension | 557,557.22 (471,196.60 to 656,787.09) | 38.83 (32.82 to 45.75) | 710,532.65 (593,118.74 to 849,603.42) | 39.06 (32.61 to 46.71) | 0.02 (0.00 to 0.04) |
| Prevalence | Glomerulonephritis | 1,744,444.61 (1,498,128.08 to 2,026,122.44) | 120.76 (103.63 to 140.34) | 2,213,374.99 (1,863,190.85 to 2,629,268.70) | 121.92 (102.64 to 144.79) | 0.04 (0.00 to 0.07) |
| Prevalence | Other and unspecified causes | 43,123,958.33 (33,232,979.46 to 54,831,003.14) | 3,001.45 (2,312.73 to 3,814.49) | 61,323,670.23 (46,753,772.92 to 78,537,607.43) | 3,371.41 (2,570.47 to 4,318.02) | 0.38 (0.36 to 0.40) |
|  | **SDI** | | | | | |
| Prevalence | High SDI | 6,026,055.12 (4,606,337.30 to 7,713,572.78) | 2,813.39 (2,151.71 to 3,602.81) | 5,714,249.46 (4,336,822.92 to 7,330,333.32) | 2,776.12 (2,107.80 to 3,563.41) | -0.04 (-0.07 to 0.00) |
| Prevalence | High-middle SDI | 9,841,909.18 (7,597,063.16 to 12,602,393.72) | 3,396.65 (2,622.14 to 4,348.25) | 8,676,933.59 (6,562,027.37 to 11,203,455.07) | 3,568.40 (2,698.46 to 4,612.31) | 0.16 (0.14 to 0.19) |
| Prevalence | Middle SDI | 19,438,489.36 (15,090,077.10 to 24,669,005.34) | 3,850.74 (2,988.74 to 4,883.19) | 23,259,297.75 (17,786,203.33 to 29,760,030.93) | 4,205.90 (3,216.40 to 5,383.25) | 0.29 (0.26 to 0.32) |
| Prevalence | Low-middle SDI | 14,310,141.73 (10,995,228.16 to 18,174,841.84) | 4,728.17 (3,630.87 to 6,003.60) | 24,546,085.54 (18,733,924.18 to 31,466,368.89) | 4,742.06 (3,618.72 to 6,077.74) | 0.03 (-0.04 to 0.10) |
| Prevalence | Low SDI | 4,649,923.23 (3,589,285.01 to 5,912,869.68) | 3,774.07 (2,912.64 to 4,796.70) | 11,270,862.45 (8,614,292.97 to 14,425,178.89) | 3,773.06 (2,883.03 to 4,824.93) | 0.02 (-0.05 to 0.08) |
|  | **Region** | | | | | |
| Prevalence | Andean Latin America | 278,535.72 (217,737.21 to 350,430.85) | 2,689.15 (2,099.89 to 3,382.62) | 482,655.42 (370,211.79 to 618,269.17) | 2,754.72 (2,113.95 to 3,527.95) | 0.08 (0.06 to 0.10) |
| Prevalence | Australasia | 121,482.57 (91,928.68 to 157,202.73) | 2,421.05 (1,832.55 to 3,134.42) | 150,291.52 (114,020.68 to 192,677.76) | 2,401.16 (1,822.45 to 3,079.63) | -0.02 (-0.03 to -0.02) |
| Prevalence | Caribbean | 299,847.62 (230,469.19 to 382,136.66) | 2,972.29 (2,284.07 to 3,786.48) | 353,759.04 (270,223.68 to 450,608.96) | 3,104.62 (2,372.09 to 3,953.83) | 0.14 (0.12 to 0.16) |
| Prevalence | Central Asia | 841,893.54 (671,870.37 to 1,047,996.35) | 4,425.65 (3,530.98 to 5,510.74) | 1,027,795.17 (814,784.22 to 1,277,775.38) | 4,515.37 (3,578.82 to 5,614.87) | 0.06 (0.06 to 0.07) |
| Prevalence | Central Europe | 745,785.80 (585,636.03 to 937,063.72) | 2,763.92 (2,170.12 to 3,472.80) | 517,468.66 (399,874.39 to 659,614.69) | 2,668.39 (2,062.60 to 3,402.69) | -0.11 (-0.13 to -0.10) |
| Prevalence | Central Latin America | 1,459,526.67 (1,137,135.36 to 1,842,394.92) | 3,197.24 (2,490.38 to 4,034.13) | 2,086,836.52 (1,623,378.18 to 2,634,163.64) | 3,257.10 (2,533.95 to 4,110.52) | 0.06 (0.05 to 0.07) |
| Prevalence | Central Sub-Saharan Africa | 482,410.64 (372,978.66 to 614,733.81) | 3,439.16 (2,657.06 to 4,382.67) | 1,201,317.58 (923,342.21 to 1,532,477.72) | 3,357.09 (2,579.92 to 4,280.55) | -0.06 (-0.09 to -0.03) |
| Prevalence | East Asia | 10,677,119.19 (8,303,490.46 to 13,600,533.98) | 2,836.49 (2,206.61 to 3,610.08) | 7,068,782.67 (5,350,598.00 to 9,150,012.96) | 2,757.70 (2,087.31 to 3,574.82) | -0.10 (-0.24 to 0.05) |
| Prevalence | Eastern Europe | 2,235,139.56 (1,713,669.06 to 2,866,622.98) | 4,371.35 (3,351.02 to 5,614.25) | 1,451,822.63 (1,099,807.86 to 1,870,954.95) | 4,359.44 (3,302.15 to 5,626.93) | -0.01 (-0.02 to -0.01) |
| Prevalence | Eastern Sub-Saharan Africa | 1,383,490.10 (1,052,060.48 to 1,777,030.73) | 2,894.35 (2,199.85 to 3,714.39) | 3,501,997.44 (2,633,637.36 to 4,534,103.07) | 2,999.38 (2,256.21 to 3,878.75) | 0.11 (0.10 to 0.12) |
| Prevalence | High-income Asia Pacific | 1,412,207.16 (1,071,093.36 to 1,823,522.70) | 3,390.26 (2,571.16 to 4,376.89) | 950,330.58 (715,045.60 to 1,228,936.80) | 3,199.12 (2,407.04 to 4,139.90) | -0.18 (-0.22 to -0.15) |
| Prevalence | High-income North America | 2,106,896.22 (1,613,186.29 to 2,687,351.64) | 3,052.81 (2,339.30 to 3,896.46) | 2,216,044.98 (1,694,024.32 to 2,826,887.67) | 2,968.81 (2,270.39 to 3,788.18) | -0.08 (-0.12 to -0.04) |
| Prevalence | North Africa and Middle East | 4,198,977.02 (3,235,640.48 to 5,379,561.02) | 4,697.84 (3,621.17 to 6,010.95) | 7,255,333.05 (5,505,821.34 to 9,361,847.13) | 4,734.96 (3,592.98 to 6,110.48) | 0.03 (0.01 to 0.04) |
| Prevalence | Oceania | 62,511.58 (48,091.35 to 79,930.56) | 3,488.27 (2,683.69 to 4,458.29) | 133,892.71 (102,359.58 to 172,125.69) | 3,629.21 (2,774.63 to 4,663.79) | 0.12 (0.10 to 0.15) |
| Prevalence | South Asia | 15,420,290.62 (11,901,118.14 to 19,589,835.24) | 5,370.99 (4,143.07 to 6,821.00) | 27,157,604.46 (20,802,153.10 to 34,663,951.84) | 5,351.64 (4,099.16 to 6,829.52) | 0.01 (-0.10 to 0.13) |
| Prevalence | Southeast Asia | 6,879,971.89 (5,261,722.52 to 8,844,363.15) | 5,254.44 (4,018.63 to 6,751.48) | 9,283,744.50 (7,020,091.83 to 11,976,838.02) | 5,370.39 (4,060.97 to 6,929.79) | 0.07 (0.06 to 0.08) |
| Prevalence | Southern Latin America | 349,041.02 (267,352.15 to 444,012.28) | 2,872.03 (2,199.52 to 3,653.42) | 481,361.59 (361,403.95 to 621,137.12) | 2,989.33 (2,244.91 to 3,858.01) | 0.13 (0.12 to 0.14) |
| Prevalence | Southern Sub-Saharan Africa | 492,962.86 (380,542.37 to 627,904.05) | 3,395.03 (2,621.48 to 4,322.50) | 712,630.36 (545,442.22 to 910,613.64) | 3,388.89 (2,593.48 to 4,332.42) | 0.00 (-0.01 to 0.01) |
| Prevalence | Tropical Latin America | 1,347,262.66 (1,041,634.61 to 1,711,994.51) | 3,169.65 (2,450.22 to 4,026.92) | 1,625,355.14 (1,240,444.53 to 2,081,705.05) | 3,013.31 (2,300.23 to 3,858.41) | -0.16 (-0.18 to -0.14) |
| Prevalence | Western Europe | 1,983,864.55 (1,523,388.67 to 2,531,513.03) | 2,157.54 (1,657.84 to 2,754.00) | 1,616,645.65 (1,227,621.04 to 2,061,410.20) | 2,123.60 (1,613.40 to 2,709.06) | -0.05 (-0.07 to -0.03) |
| Prevalence | Western Sub-Saharan Africa | 1,528,353.36 (1,197,415.94 to 1,933,509.34) | 3,181.19 (2,491.86 to 4,024.99) | 4,241,573.01 (3,266,402.90 to 5,392,304.50) | 3,328.77 (2,562.77 to 4,231.84) | 0.15 (0.14 to 0.17) |
|  | **Country** | | | | | |
| Prevalence | Afghanistan | 92,333.58 (71,086.05 to 119,040.13) | 4,483.13 (3,459.63 to 5,741.28) | 403,277.36 (305,944.34 to 519,378.31) | 4,589.71 (3,481.70 to 5,897.99) | 0.08 (0.07 to 0.09) |
| Prevalence | Albania | 26,083.61 (20,482.00 to 32,783.65) | 2,764.65 (2,170.98 to 3,474.82) | 16,872.72 (13,105.60 to 21,419.28) | 2,752.53 (2,138.53 to 3,495.88) | -0.01 (-0.02 to -0.01) |
| Prevalence | Algeria | 327,112.23 (249,178.16 to 419,113.23) | 4,709.58 (3,591.05 to 6,024.71) | 468,760.34 (356,245.12 to 600,859.62) | 4,661.46 (3,540.08 to 5,988.09) | -0.04 (-0.05 to -0.02) |
| Prevalence | American Samoa | 564.71 (430.01 to 725.46) | 4,086.95 (3,112.03 to 5,246.26) | 458.74 (354.22 to 587.69) | 4,353.61 (3,357.97 to 5,581.24) | 0.20 (0.19 to 0.21) |
| Prevalence | Andorra | 350.57 (268.58 to 448.54) | 2,252.52 (1,729.27 to 2,886.20) | 318.69 (240.53 to 410.09) | 2,260.91 (1,707.04 to 2,909.10) | 0.01 (-0.01 to 0.04) |
| Prevalence | Angola | 87,342.04 (67,517.35 to 111,605.67) | 3,350.12 (2,587.85 to 4,280.47) | 273,277.66 (209,332.60 to 350,174.56) | 3,401.45 (2,605.82 to 4,355.93) | 0.06 (0.05 to 0.07) |
| Prevalence | Antigua and Barbuda | 527.96 (405.39 to 675.33) | 3,077.62 (2,363.81 to 3,935.81) | 689.68 (530.33 to 879.88) | 3,223.32 (2,480.41 to 4,110.55) | 0.15 (0.13 to 0.16) |
| Prevalence | Argentina | 214,519.09 (164,752.47 to 273,884.06) | 2,802.15 (2,151.69 to 3,577.58) | 325,863.39 (243,788.76 to 422,003.28) | 2,981.97 (2,231.13 to 3,861.70) | 0.20 (0.18 to 0.22) |
| Prevalence | Armenia | 39,821.22 (31,198.30 to 50,633.36) | 4,289.09 (3,359.46 to 5,457.78) | 25,327.16 (19,747.24 to 32,279.09) | 4,240.30 (3,308.71 to 5,412.06) | -0.03 (-0.05 to -0.02) |
| Prevalence | Australia | 99,790.75 (75,195.67 to 129,420.77) | 2,402.96 (1,811.38 to 3,117.90) | 122,521.75 (92,674.08 to 157,462.79) | 2,385.49 (1,805.60 to 3,066.91) | -0.02 (-0.03 to -0.01) |
| Prevalence | Austria | 43,973.99 (33,079.35 to 56,520.71) | 2,206.16 (1,659.79 to 2,837.24) | 38,910.36 (29,264.30 to 49,947.94) | 2,306.46 (1,733.87 to 2,964.78) | 0.15 (0.14 to 0.16) |
| Prevalence | Azerbaijan | 96,219.13 (76,873.12 to 119,905.91) | 4,407.95 (3,522.40 to 5,493.13) | 113,567.15 (90,185.40 to 141,748.17) | 4,510.63 (3,587.27 to 5,627.54) | 0.08 (0.07 to 0.08) |
| Prevalence | Bahamas | 2,384.49 (1,847.46 to 3,026.70) | 2,993.45 (2,319.79 to 3,798.70) | 3,045.79 (2,352.91 to 3,855.94) | 3,169.48 (2,448.36 to 4,013.69) | 0.18 (0.16 to 0.21) |
| Prevalence | Bahrain | 7,840.67 (5,941.36 to 10,117.84) | 4,762.34 (3,601.17 to 6,174.79) | 19,932.34 (14,936.91 to 25,931.11) | 4,807.02 (3,597.64 to 6,267.28) | 0.03 (0.02 to 0.04) |
| Prevalence | Bangladesh | 1,303,419.13 (992,372.61 to 1,665,483.18) | 4,505.78 (3,426.52 to 5,755.01) | 2,065,194.39 (1,567,663.57 to 2,647,250.26) | 4,680.35 (3,552.59 to 5,999.15) | 0.12 (0.11 to 0.13) |
| Prevalence | Barbados | 2,050.53 (1,557.31 to 2,607.30) | 2,968.97 (2,255.60 to 3,774.23) | 1,855.87 (1,417.28 to 2,384.29) | 3,112.89 (2,378.31 to 3,998.40) | 0.15 (0.12 to 0.18) |
| Prevalence | Belarus | 105,042.54 (79,948.18 to 136,219.41) | 4,330.45 (3,293.10 to 5,629.58) | 66,152.31 (50,092.35 to 86,290.97) | 4,349.38 (3,289.92 to 5,678.34) | 0.01 (-0.01 to 0.03) |
| Prevalence | Belgium | 50,333.17 (38,321.29 to 63,938.94) | 2,178.62 (1,660.10 to 2,770.13) | 48,190.22 (36,428.18 to 61,461.14) | 2,308.92 (1,745.95 to 2,946.37) | 0.19 (0.18 to 0.20) |
| Prevalence | Belize | 1,505.21 (1,166.13 to 1,893.99) | 3,005.85 (2,326.46 to 3,780.43) | 3,960.75 (3,061.65 to 5,041.35) | 3,224.21 (2,491.51 to 4,101.76) | 0.22 (0.19 to 0.26) |
| Prevalence | Benin | 36,205.51 (27,704.11 to 45,885.09) | 3,141.81 (2,402.55 to 3,981.18) | 116,844.35 (89,064.58 to 149,469.57) | 3,277.46 (2,499.22 to 4,188.96) | 0.15 (0.12 to 0.18) |
| Prevalence | Bermuda | 451.52 (345.62 to 575.00) | 2,860.10 (2,191.32 to 3,648.22) | 271.34 (207.18 to 348.26) | 2,841.46 (2,169.24 to 3,650.67) | -0.02 (-0.05 to 0.01) |
| Prevalence | Bhutan | 8,652.51 (6,724.48 to 10,934.17) | 4,596.78 (3,571.06 to 5,804.65) | 10,314.81 (7,924.26 to 13,131.32) | 4,684.61 (3,601.84 to 5,963.52) | 0.06 (0.05 to 0.07) |
| Prevalence | Bolivia (Plurinational State of) | 44,839.17 (35,064.49 to 56,478.82) | 2,765.77 (2,160.07 to 3,482.91) | 87,891.73 (67,638.50 to 113,174.71) | 2,751.76 (2,117.99 to 3,542.65) | -0.02 (-0.03 to -0.01) |
| Prevalence | Bosnia and Herzegovina | 30,722.01 (23,568.92 to 38,770.50) | 2,567.14 (1,969.89 to 3,241.02) | 16,073.58 (12,316.87 to 20,771.11) | 2,716.72 (2,081.16 to 3,511.57) | 0.18 (0.15 to 0.21) |
| Prevalence | Botswana | 11,182.57 (8,612.29 to 14,345.70) | 3,231.81 (2,489.57 to 4,142.25) | 21,949.12 (16,763.65 to 28,195.15) | 3,368.45 (2,571.80 to 4,330.42) | 0.15 (0.13 to 0.16) |
| Prevalence | Brazil | 1,316,731.57 (1,018,100.60 to 1,672,809.77) | 3,174.98 (2,454.50 to 4,032.72) | 1,566,338.77 (1,194,521.22 to 2,007,269.19) | 3,012.69 (2,298.06 to 3,859.81) | -0.17 (-0.19 to -0.15) |
| Prevalence | Brunei Darussalam | 3,269.82 (2,496.04 to 4,180.40) | 4,068.95 (3,109.88 to 5,206.10) | 4,976.93 (3,827.41 to 6,341.19) | 4,053.29 (3,118.37 to 5,166.17) | -0.01 (-0.03 to 0.00) |
| Prevalence | Bulgaria | 46,904.33 (36,289.83 to 59,834.92) | 2,658.53 (2,056.81 to 3,391.57) | 27,050.23 (21,277.01 to 33,977.56) | 2,726.14 (2,145.24 to 3,426.39) | 0.08 (0.07 to 0.08) |
| Prevalence | Burkina Faso | 63,376.94 (48,892.48 to 80,813.52) | 2,989.76 (2,303.08 to 3,810.56) | 175,542.89 (134,263.78 to 224,209.97) | 3,102.35 (2,371.01 to 3,962.28) | 0.13 (0.11 to 0.14) |
| Prevalence | Burundi | 41,749.33 (31,416.89 to 53,890.50) | 3,004.81 (2,260.62 to 3,877.52) | 103,713.58 (78,032.64 to 135,161.86) | 3,069.05 (2,309.67 to 3,995.87) | 0.07 (0.05 to 0.09) |
| Prevalence | Cabo Verde | 3,048.08 (2,363.23 to 3,891.37) | 3,263.35 (2,529.59 to 4,163.78) | 5,125.88 (3,885.58 to 6,574.07) | 3,305.96 (2,506.18 to 4,242.58) | 0.05 (0.03 to 0.07) |
| Prevalence | Cambodia | 125,355.86 (95,153.79 to 162,667.47) | 4,766.41 (3,616.84 to 6,185.73) | 220,264.65 (166,123.37 to 284,388.72) | 4,794.89 (3,616.58 to 6,193.34) | 0.02 (0.01 to 0.03) |
| Prevalence | Cameroon | 81,249.68 (63,211.36 to 103,556.83) | 3,166.66 (2,462.71 to 4,037.13) | 290,875.38 (224,492.96 to 370,055.09) | 3,427.07 (2,643.53 to 4,360.05) | 0.26 (0.23 to 0.29) |
| Prevalence | Canada | 223,051.85 (166,866.32 to 286,140.05) | 3,302.08 (2,472.57 to 4,244.18) | 238,012.03 (179,587.11 to 305,441.25) | 3,375.79 (2,548.03 to 4,334.82) | 0.07 (0.06 to 0.08) |
| Prevalence | Central African Republic | 24,008.13 (18,473.23 to 30,645.48) | 3,362.79 (2,587.34 to 4,291.75) | 48,586.44 (37,575.17 to 62,116.28) | 3,392.24 (2,623.91 to 4,333.52) | 0.04 (0.02 to 0.06) |
| Prevalence | Chad | 42,193.05 (31,994.23 to 53,965.25) | 3,027.29 (2,292.63 to 3,871.13) | 131,600.44 (100,368.27 to 169,808.61) | 3,105.45 (2,365.83 to 4,008.20) | 0.09 (0.08 to 0.10) |
| Prevalence | Chile | 114,062.45 (86,686.24 to 146,794.32) | 3,014.22 (2,290.97 to 3,879.15) | 133,133.06 (100,280.32 to 171,379.90) | 3,007.90 (2,267.57 to 3,874.33) | -0.01 (-0.03 to 0.01) |
| Prevalence | China | 10,361,958.34 (8,058,048.02 to 13,200,446.96) | 2,839.76 (2,209.12 to 3,614.66) | 6,784,694.69 (5,130,750.00 to 8,784,251.76) | 2,762.67 (2,088.90 to 3,582.22) | -0.10 (-0.24 to 0.05) |
| Prevalence | Colombia | 284,923.36 (220,036.45 to 362,797.01) | 3,007.97 (2,323.04 to 3,829.24) | 391,148.30 (298,185.27 to 502,008.83) | 2,986.80 (2,278.74 to 3,831.21) | -0.02 (-0.04 to 0.00) |
| Prevalence | Comoros | 3,535.59 (2,676.26 to 4,531.55) | 3,014.12 (2,280.13 to 3,858.76) | 6,114.11 (4,545.17 to 7,896.91) | 3,026.04 (2,249.24 to 3,906.58) | 0.01 (0.01 to 0.02) |
| Prevalence | Congo | 22,596.22 (17,482.42 to 28,715.21) | 3,499.76 (2,707.19 to 4,444.55) | 49,166.93 (37,562.43 to 63,022.74) | 3,507.41 (2,680.03 to 4,495.95) | 0.02 (0.00 to 0.04) |
| Prevalence | Cook Islands | 189.73 (143.30 to 245.66) | 3,667.82 (2,770.04 to 4,747.90) | 143.64 (108.98 to 186.53) | 3,813.19 (2,893.03 to 4,949.99) | 0.12 (0.09 to 0.15) |
| Prevalence | Costa Rica | 28,908.05 (22,396.96 to 36,755.81) | 3,352.68 (2,597.69 to 4,261.90) | 39,491.22 (30,439.95 to 50,440.87) | 3,363.05 (2,594.74 to 4,295.87) | 0.01 (-0.01 to 0.02) |
| Prevalence | Coted'Ivoire | 105,813.94 (81,780.38 to 134,967.47) | 3,242.91 (2,504.82 to 4,133.13) | 239,103.64 (182,261.78 to 307,016.10) | 3,313.58 (2,524.63 to 4,252.87) | 0.08 (0.06 to 0.10) |
| Prevalence | Croatia | 28,729.77 (22,076.72 to 36,488.44) | 2,674.01 (2,055.77 to 3,398.44) | 19,335.11 (14,637.63 to 24,956.17) | 2,669.72 (2,021.21 to 3,446.38) | -0.01 (-0.01 to 0.00) |
| Prevalence | Cuba | 99,975.34 (76,677.92 to 127,719.80) | 2,913.74 (2,235.13 to 3,721.26) | 60,941.57 (46,583.03 to 78,509.55) | 2,857.63 (2,185.39 to 3,681.12) | -0.06 (-0.08 to -0.05) |
| Prevalence | Cyprus | 4,480.12 (3,394.50 to 5,727.52) | 2,302.29 (1,744.47 to 2,944.65) | 6,318.18 (4,786.63 to 8,106.22) | 2,318.22 (1,757.81 to 2,977.06) | 0.03 (0.02 to 0.03) |
| Prevalence | Czechia | 57,578.80 (44,530.92 to 73,087.95) | 2,681.81 (2,074.05 to 3,405.58) | 40,806.39 (31,160.73 to 51,985.16) | 2,571.54 (1,964.71 to 3,279.74) | -0.14 (-0.19 to -0.09) |
| Prevalence | Democratic People's Republic of Korea | 154,900.28 (120,289.18 to 196,463.95) | 2,737.32 (2,126.29 to 3,470.69) | 166,055.61 (127,150.37 to 212,494.60) | 2,669.35 (2,045.22 to 3,417.74) | -0.09 (-0.10 to -0.07) |
| Prevalence | Democratic Republic of the Congo | 336,280.29 (259,944.46 to 428,210.51) | 3,466.81 (2,677.88 to 4,414.28) | 796,386.54 (609,857.64 to 1,019,729.58) | 3,324.92 (2,544.62 to 4,255.09) | -0.12 (-0.15 to -0.09) |
| Prevalence | Denmark | 26,844.60 (20,394.23 to 34,435.91) | 2,240.62 (1,703.82 to 2,874.65) | 26,983.91 (20,515.93 to 34,803.81) | 2,307.27 (1,756.56 to 2,976.21) | 0.10 (0.09 to 0.11) |
| Prevalence | Djibouti | 3,365.53 (2,549.23 to 4,368.21) | 2,841.18 (2,151.41 to 3,679.36) | 10,016.16 (7,542.74 to 12,977.96) | 3,000.50 (2,259.72 to 3,886.92) | 0.18 (0.16 to 0.20) |
| Prevalence | Dominica | 646.09 (507.89 to 810.01) | 3,283.74 (2,579.08 to 4,115.71) | 558.47 (439.68 to 700.95) | 3,491.45 (2,748.14 to 4,383.09) | 0.20 (0.19 to 0.21) |
| Prevalence | Dominican Republic | 61,208.48 (46,938.09 to 78,388.41) | 2,905.24 (2,226.27 to 3,717.31) | 90,256.12 (69,141.57 to 114,646.30) | 3,083.39 (2,362.85 to 3,916.83) | 0.19 (0.17 to 0.22) |
| Prevalence | Ecuador | 75,327.06 (58,372.56 to 96,130.86) | 2,729.15 (2,114.36 to 3,481.28) | 134,927.13 (103,263.53 to 171,258.92) | 2,831.57 (2,167.75 to 3,592.96) | 0.12 (0.11 to 0.13) |
| Prevalence | Egypt | 635,903.45 (485,922.85 to 816,607.54) | 4,383.72 (3,351.06 to 5,623.12) | 1,260,189.71 (945,958.99 to 1,627,035.49) | 4,749.48 (3,565.70 to 6,127.50) | 0.26 (0.23 to 0.28) |
| Prevalence | El Salvador | 40,150.99 (30,618.57 to 51,569.74) | 2,849.27 (2,171.65 to 3,656.42) | 55,535.01 (42,576.15 to 70,815.79) | 3,133.83 (2,403.57 to 3,994.28) | 0.31 (0.28 to 0.33) |
| Prevalence | Equatorial Guinea | 3,377.74 (2,626.86 to 4,350.62) | 3,385.24 (2,632.77 to 4,360.68) | 16,708.13 (12,774.42 to 21,464.64) | 3,557.78 (2,721.60 to 4,566.40) | 0.17 (0.15 to 0.19) |
| Prevalence | Eritrea | 24,095.34 (18,034.44 to 31,357.89) | 2,770.95 (2,073.26 to 3,606.51) | 53,227.13 (39,761.30 to 69,461.37) | 2,883.34 (2,154.22 to 3,761.67) | 0.13 (0.11 to 0.14) |
| Prevalence | Estonia | 14,704.35 (11,105.06 to 19,061.44) | 4,288.62 (3,237.15 to 5,567.35) | 8,941.67 (6,731.82 to 11,594.75) | 4,208.81 (3,168.86 to 5,464.36) | -0.06 (-0.07 to -0.05) |
| Prevalence | Eswatini | 6,545.82 (5,071.87 to 8,384.38) | 3,206.81 (2,485.05 to 4,107.33) | 11,345.28 (8,667.64 to 14,504.86) | 3,407.98 (2,603.42 to 4,354.97) | 0.21 (0.19 to 0.22) |
| Prevalence | Ethiopia | 341,830.35 (264,285.75 to 434,546.89) | 2,784.01 (2,151.57 to 3,537.07) | 891,435.82 (674,239.75 to 1,153,843.29) | 2,853.38 (2,159.31 to 3,688.88) | 0.08 (0.07 to 0.09) |
| Prevalence | Fiji | 8,403.33 (6,459.46 to 10,759.75) | 3,943.34 (3,030.83 to 5,048.16) | 9,294.62 (7,147.94 to 11,925.18) | 4,195.81 (3,226.60 to 5,383.41) | 0.19 (0.17 to 0.22) |
| Prevalence | Finland | 25,690.59 (19,401.04 to 33,133.92) | 2,383.43 (1,800.01 to 3,075.60) | 22,800.44 (17,090.38 to 29,605.86) | 2,316.51 (1,736.25 to 3,010.47) | -0.09 (-0.10 to -0.09) |
| Prevalence | France | 241,408.35 (183,141.95 to 312,627.30) | 1,800.85 (1,366.52 to 2,332.94) | 211,077.05 (156,655.33 to 274,398.86) | 1,795.88 (1,332.64 to 2,334.27) | -0.01 (-0.02 to 0.00) |
| Prevalence | Gabon | 8,806.21 (6,848.22 to 11,195.09) | 3,381.21 (2,628.91 to 4,296.71) | 17,191.89 (13,183.47 to 21,954.78) | 3,476.48 (2,664.97 to 4,439.22) | 0.09 (0.08 to 0.11) |
| Prevalence | Gambia | 8,081.65 (6,192.10 to 10,367.35) | 3,098.70 (2,373.17 to 3,975.69) | 21,879.84 (16,659.24 to 28,092.12) | 3,263.45 (2,483.66 to 4,186.96) | 0.17 (0.15 to 0.20) |
| Prevalence | Georgia | 60,544.60 (47,313.54 to 76,439.67) | 4,442.00 (3,470.80 to 5,609.49) | 28,046.25 (21,792.58 to 35,464.11) | 4,348.65 (3,380.69 to 5,499.77) | -0.07 (-0.08 to -0.06) |
| Prevalence | Germany | 387,209.05 (294,204.64 to 498,021.88) | 2,001.81 (1,522.79 to 2,577.15) | 283,999.66 (214,091.31 to 366,535.62) | 1,934.56 (1,459.37 to 2,498.04) | -0.11 (-0.13 to -0.09) |
| Prevalence | Ghana | 112,792.69 (85,304.19 to 146,856.77) | 2,949.21 (2,231.60 to 3,836.02) | 289,529.42 (217,193.51 to 374,159.39) | 3,074.54 (2,306.28 to 3,971.12) | 0.14 (0.13 to 0.15) |
| Prevalence | Greece | 52,235.11 (39,670.09 to 66,593.88) | 2,225.89 (1,690.66 to 2,837.24) | 34,440.95 (26,229.31 to 44,566.96) | 2,206.96 (1,680.83 to 2,856.48) | -0.02 (-0.04 to 0.00) |
| Prevalence | Greenland | 566.51 (431.42 to 729.94) | 3,076.51 (2,339.80 to 3,971.18) | 391.88 (296.05 to 502.87) | 3,134.43 (2,366.62 to 4,030.37) | 0.06 (0.04 to 0.08) |
| Prevalence | Grenada | 710.36 (550.56 to 901.20) | 3,234.62 (2,505.66 to 4,105.00) | 934.24 (719.74 to 1,180.91) | 3,403.29 (2,624.04 to 4,298.50) | 0.16 (0.13 to 0.19) |
| Prevalence | Guam | 1,538.50 (1,185.13 to 1,983.71) | 3,629.15 (2,796.53 to 4,676.29) | 1,436.97 (1,109.62 to 1,839.71) | 4,002.85 (3,091.42 to 5,124.45) | 0.31 (0.29 to 0.34) |
| Prevalence | Guatemala | 57,492.20 (44,518.56 to 72,708.01) | 2,950.25 (2,282.01 to 3,734.05) | 154,763.09 (120,022.18 to 194,777.88) | 3,374.90 (2,616.97 to 4,244.79) | 0.43 (0.39 to 0.47) |
| Prevalence | Guinea | 41,687.08 (32,433.16 to 52,804.41) | 3,126.45 (2,430.73 to 3,959.39) | 111,997.51 (86,369.72 to 143,680.50) | 3,277.71 (2,526.14 to 4,206.11) | 0.16 (0.14 to 0.18) |
| Prevalence | Guinea-Bissau | 8,057.02 (6,228.02 to 10,213.44) | 3,248.66 (2,508.88 to 4,118.91) | 18,458.20 (14,130.91 to 23,588.83) | 3,312.43 (2,535.81 to 4,231.84) | 0.07 (0.05 to 0.08) |
| Prevalence | Guyana | 7,131.49 (5,518.04 to 9,099.38) | 3,088.38 (2,389.16 to 3,938.27) | 7,024.93 (5,397.11 to 8,928.49) | 3,240.18 (2,490.58 to 4,115.39) | 0.15 (0.12 to 0.19) |
| Prevalence | Haiti | 49,342.31 (37,930.72 to 63,049.47) | 3,106.33 (2,385.43 to 3,969.54) | 111,578.35 (85,029.67 to 142,587.88) | 3,229.14 (2,460.38 to 4,126.10) | 0.12 (0.12 to 0.13) |
| Prevalence | Honduras | 35,337.93 (27,423.80 to 44,792.34) | 3,106.34 (2,408.80 to 3,936.10) | 90,962.90 (69,783.78 to 115,947.54) | 3,078.90 (2,362.14 to 3,920.84) | -0.03 (-0.05 to -0.01) |
| Prevalence | Hungary | 55,170.45 (42,482.08 to 70,087.88) | 2,724.51 (2,096.86 to 3,461.26) | 41,882.16 (32,370.20 to 53,342.32) | 2,607.52 (2,013.58 to 3,324.39) | -0.14 (-0.17 to -0.12) |
| Prevalence | Iceland | 1,255.03 (946.90 to 1,613.86) | 1,911.64 (1,442.37 to 2,459.12) | 1,448.04 (1,086.64 to 1,872.20) | 1,959.70 (1,471.52 to 2,536.30) | 0.09 (0.06 to 0.11) |
| Prevalence | India | 12,625,425.35 (9,728,663.22 to 16,069,346.52) | 5,599.83 (4,313.73 to 7,125.14) | 21,512,667.62 (16,400,009.71 to 27,556,531.58) | 5,536.00 (4,220.31 to 7,090.12) | 0.00 (-0.15 to 0.14) |
| Prevalence | Indonesia | 2,782,412.70 (2,127,011.21 to 3,577,312.86) | 5,386.90 (4,116.88 to 6,922.90) | 3,949,784.03 (2,979,078.33 to 5,095,712.22) | 5,563.54 (4,197.23 to 7,178.53) | 0.11 (0.09 to 0.12) |
| Prevalence | Iran (Islamic Republic of) | 643,115.32 (503,491.63 to 810,214.68) | 4,402.71 (3,443.17 to 5,544.78) | 772,199.95 (593,603.03 to 981,731.55) | 4,256.64 (3,272.80 to 5,419.14) | -0.11 (-0.12 to -0.09) |
| Prevalence | Iraq | 243,863.48 (186,577.16 to 311,893.20) | 5,013.54 (3,834.81 to 6,400.52) | 572,536.90 (433,247.99 to 737,781.90) | 4,995.00 (3,780.31 to 6,434.10) | -0.01 (-0.02 to 0.00) |
| Prevalence | Ireland | 22,430.06 (16,974.72 to 28,824.60) | 2,653.27 (2,006.98 to 3,407.76) | 24,339.44 (18,682.06 to 31,051.04) | 2,732.91 (2,096.95 to 3,485.17) | 0.10 (0.08 to 0.13) |
| Prevalence | Israel | 27,376.00 (21,016.62 to 34,795.42) | 2,328.23 (1,786.72 to 2,958.66) | 47,103.08 (35,855.18 to 60,674.93) | 2,295.16 (1,746.59 to 2,956.07) | -0.04 (-0.07 to -0.02) |
| Prevalence | Italy | 330,712.03 (254,030.39 to 420,098.89) | 2,392.94 (1,839.04 to 3,039.32) | 213,658.11 (164,516.94 to 271,987.88) | 2,322.62 (1,789.67 to 2,957.64) | -0.10 (-0.12 to -0.09) |
| Prevalence | Jamaica | 19,629.08 (15,282.30 to 25,014.47) | 2,914.55 (2,268.46 to 3,711.08) | 24,132.34 (18,439.23 to 30,816.86) | 3,072.68 (2,349.63 to 3,924.12) | 0.17 (0.15 to 0.19) |
| Prevalence | Japan | 948,605.19 (717,259.01 to 1,226,763.84) | 3,516.85 (2,659.16 to 4,546.76) | 626,101.11 (471,205.38 to 811,108.78) | 3,320.94 (2,499.43 to 4,303.32) | -0.18 (-0.20 to -0.16) |
| Prevalence | Jordan | 52,584.86 (40,227.54 to 67,446.51) | 4,809.64 (3,680.00 to 6,154.16) | 163,797.56 (123,977.17 to 211,147.52) | 4,710.89 (3,566.23 to 6,067.72) | -0.07 (-0.08 to -0.05) |
| Prevalence | Kazakhstan | 195,061.77 (153,708.54 to 245,078.22) | 4,462.76 (3,516.22 to 5,608.91) | 180,340.53 (141,183.76 to 229,215.69) | 4,443.65 (3,476.25 to 5,652.30) | -0.01 (-0.02 to 0.00) |
| Prevalence | Kenya | 176,160.31 (132,717.65 to 228,228.83) | 2,930.77 (2,207.82 to 3,792.12) | 424,448.57 (318,602.30 to 550,644.81) | 3,010.32 (2,259.63 to 3,901.80) | 0.09 (0.07 to 0.10) |
| Prevalence | Kiribati | 803.01 (621.43 to 1,024.41) | 3,824.14 (2,959.12 to 4,874.74) | 1,285.54 (995.26 to 1,640.08) | 4,005.29 (3,100.54 to 5,110.60) | 0.14 (0.13 to 0.15) |
| Prevalence | Kuwait | 25,739.68 (19,411.89 to 33,132.53) | 4,746.28 (3,581.36 to 6,128.22) | 50,182.16 (37,866.07 to 64,785.50) | 4,617.45 (3,480.49 to 5,986.41) | -0.09 (-0.10 to -0.08) |
| Prevalence | Kyrgyzstan | 54,194.79 (43,716.31 to 66,293.41) | 4,511.54 (3,637.31 to 5,522.28) | 72,791.61 (57,534.75 to 90,651.42) | 4,197.54 (3,316.91 to 5,229.61) | -0.24 (-0.26 to -0.22) |
| Prevalence | Lao People's Democratic Republic | 55,088.14 (42,099.70 to 70,224.96) | 5,411.32 (4,132.76 to 6,898.56) | 110,410.98 (84,326.04 to 141,676.61) | 5,328.71 (4,069.98 to 6,837.74) | -0.05 (-0.05 to -0.04) |
| Prevalence | Latvia | 25,642.57 (19,363.59 to 33,098.45) | 4,329.97 (3,269.61 to 5,595.13) | 12,520.49 (9,379.66 to 16,137.09) | 4,317.41 (3,231.57 to 5,584.25) | -0.01 (-0.03 to 0.01) |
| Prevalence | Lebanon | 36,046.68 (27,316.39 to 46,204.07) | 4,625.25 (3,506.18 to 5,924.95) | 67,084.68 (50,953.09 to 86,173.25) | 4,827.09 (3,659.27 to 6,226.96) | 0.14 (0.13 to 0.15) |
| Prevalence | Lesotho | 10,598.12 (8,125.47 to 13,642.83) | 3,052.75 (2,340.52 to 3,927.78) | 17,927.23 (13,589.54 to 23,235.61) | 3,266.48 (2,475.85 to 4,228.78) | 0.23 (0.22 to 0.24) |
| Prevalence | Liberia | 20,146.72 (15,512.58 to 25,554.62) | 3,256.38 (2,505.94 to 4,130.77) | 47,929.69 (36,484.95 to 61,315.20) | 3,301.54 (2,510.78 to 4,219.83) | 0.05 (0.03 to 0.06) |
| Prevalence | Libya | 53,095.95 (40,111.92 to 68,784.37) | 4,723.18 (3,571.12 to 6,107.17) | 87,777.00 (66,519.19 to 113,536.23) | 4,936.01 (3,739.90 to 6,388.61) | 0.14 (0.14 to 0.15) |
| Prevalence | Lithuania | 39,251.29 (29,691.53 to 51,106.66) | 4,391.13 (3,321.21 to 5,722.30) | 21,750.21 (16,383.93 to 28,326.27) | 4,401.14 (3,313.90 to 5,739.29) | 0.00 (0.00 to 0.01) |
| Prevalence | Luxembourg | 2,265.22 (1,715.88 to 2,891.95) | 2,484.23 (1,882.98 to 3,175.83) | 3,192.75 (2,418.61 to 4,096.21) | 2,513.89 (1,905.77 to 3,228.87) | 0.03 (0.00 to 0.06) |
| Prevalence | Madagascar | 88,069.94 (66,405.94 to 113,361.28) | 2,902.11 (2,187.17 to 3,731.85) | 230,015.04 (173,175.12 to 298,708.23) | 2,935.65 (2,211.74 to 3,809.36) | 0.04 (0.02 to 0.05) |
| Prevalence | Malawi | 79,531.91 (60,077.92 to 102,832.96) | 3,064.66 (2,313.65 to 3,956.75) | 172,145.13 (128,084.84 to 223,839.62) | 3,199.87 (2,378.42 to 4,151.54) | 0.14 (0.13 to 0.15) |
| Prevalence | Malaysia | 255,840.13 (193,253.47 to 329,716.63) | 5,278.28 (3,986.94 to 6,801.39) | 481,695.28 (363,169.05 to 626,748.34) | 5,479.79 (4,131.63 to 7,136.18) | 0.13 (0.10 to 0.15) |
| Prevalence | Maldives | 3,021.63 (2,292.06 to 3,873.83) | 5,279.55 (4,003.36 to 6,761.93) | 7,294.48 (5,488.95 to 9,449.52) | 5,263.02 (3,963.04 to 6,829.75) | -0.01 (-0.02 to 0.00) |
| Prevalence | Mali | 61,743.86 (48,491.60 to 78,002.06) | 3,122.64 (2,451.13 to 3,944.97) | 195,236.49 (150,551.48 to 248,509.29) | 3,214.34 (2,477.75 to 4,086.98) | 0.10 (0.09 to 0.12) |
| Prevalence | Malta | 1,868.15 (1,423.21 to 2,398.87) | 2,344.41 (1,787.61 to 3,012.95) | 1,747.61 (1,325.50 to 2,238.51) | 2,306.66 (1,751.78 to 2,956.15) | -0.05 (-0.06 to -0.04) |
| Prevalence | Marshall Islands | 411.10 (315.49 to 526.82) | 3,674.37 (2,818.08 to 4,705.63) | 572.81 (441.85 to 725.59) | 3,883.75 (2,994.93 to 4,917.34) | 0.18 (0.16 to 0.19) |
| Prevalence | Mauritania | 16,409.22 (12,673.78 to 20,792.45) | 3,208.60 (2,475.84 to 4,063.74) | 35,727.70 (27,256.23 to 45,734.50) | 3,153.39 (2,404.75 to 4,034.47) | -0.05 (-0.06 to -0.04) |
| Prevalence | Mauritius | 17,483.12 (13,242.70 to 22,669.73) | 5,350.64 (4,054.61 to 6,937.61) | 16,561.84 (12,750.54 to 21,432.16) | 5,758.15 (4,434.49 to 7,454.06) | 0.24 (0.23 to 0.25) |
| Prevalence | Mexico | 785,860.35 (615,259.32 to 990,062.33) | 3,293.90 (2,578.02 to 4,149.85) | 1,090,829.56 (857,141.62 to 1,363,124.33) | 3,365.34 (2,644.66 to 4,204.67) | 0.07 (0.05 to 0.09) |
| Prevalence | Micronesia (Federated States of) | 954.72 (736.02 to 1,212.70) | 3,745.31 (2,882.61 to 4,763.68) | 1,115.40 (863.96 to 1,419.28) | 3,995.12 (3,092.31 to 5,082.07) | 0.20 (0.18 to 0.22) |
| Prevalence | Monaco | 124.46 (94.28 to 160.20) | 2,251.14 (1,705.28 to 2,898.33) | 123.42 (93.50 to 158.72) | 2,265.15 (1,715.99 to 2,912.62) | 0.02 (0.01 to 0.03) |
| Prevalence | Mongolia | 28,556.37 (23,059.74 to 35,218.08) | 4,631.74 (3,740.12 to 5,712.15) | 32,356.93 (25,623.04 to 40,665.77) | 4,372.03 (3,461.71 to 5,498.16) | -0.19 (-0.20 to -0.17) |
| Prevalence | Montenegro | 4,445.55 (3,474.38 to 5,573.14) | 2,832.18 (2,214.03 to 3,551.00) | 3,484.18 (2,701.80 to 4,445.62) | 2,845.47 (2,207.52 to 3,630.85) | 0.01 (0.00 to 0.03) |
| Prevalence | Morocco | 335,411.11 (253,270.18 to 435,135.38) | 4,798.09 (3,624.33 to 6,215.17) | 436,563.93 (324,345.23 to 565,975.79) | 4,806.83 (3,570.85 to 6,233.56) | 0.01 (-0.01 to 0.03) |
| Prevalence | Mozambique | 93,772.52 (70,470.73 to 121,237.88) | 3,081.46 (2,310.18 to 3,982.99) | 267,380.60 (202,070.17 to 349,644.02) | 3,246.47 (2,452.03 to 4,241.61) | 0.17 (0.16 to 0.18) |
| Prevalence | Myanmar | 617,609.05 (471,640.02 to 787,705.80) | 5,365.46 (4,097.93 to 6,839.08) | 756,215.10 (574,545.90 to 968,797.68) | 5,276.23 (4,007.82 to 6,756.99) | -0.06 (-0.08 to -0.03) |
| Prevalence | Namibia | 12,256.77 (9,366.36 to 15,706.24) | 3,220.45 (2,462.52 to 4,123.20) | 21,994.70 (16,619.55 to 28,201.79) | 3,200.87 (2,418.73 to 4,103.29) | -0.02 (-0.03 to 0.00) |
| Prevalence | Nauru | 103.05 (79.48 to 131.48) | 3,974.05 (3,062.01 to 5,070.09) | 127.83 (98.97 to 163.20) | 4,254.19 (3,292.58 to 5,431.60) | 0.22 (0.20 to 0.23) |
| Prevalence | Nepal | 212,951.41 (170,238.70 to 263,533.77) | 4,351.57 (3,477.62 to 5,386.17) | 397,038.55 (315,745.78 to 490,182.80) | 4,406.30 (3,504.58 to 5,440.55) | 0.04 (0.02 to 0.07) |
| Prevalence | Netherlands | 104,542.67 (80,068.71 to 134,144.46) | 2,740.74 (2,099.47 to 3,519.67) | 89,753.84 (68,178.17 to 115,768.14) | 2,754.94 (2,093.22 to 3,553.49) | 0.02 (0.00 to 0.04) |
| Prevalence | New Zealand | 21,691.82 (16,556.77 to 27,777.33) | 2,508.50 (1,914.74 to 3,213.05) | 27,769.77 (21,084.49 to 35,610.88) | 2,472.90 (1,877.47 to 3,173.94) | -0.05 (-0.07 to -0.03) |
| Prevalence | Nicaragua | 34,546.55 (27,360.48 to 43,141.04) | 3,466.07 (2,742.74 to 4,327.52) | 66,422.31 (52,213.55 to 82,445.24) | 3,616.94 (2,843.55 to 4,488.67) | 0.14 (0.11 to 0.17) |
| Prevalence | Niger | 52,751.68 (41,018.12 to 67,199.87) | 2,843.65 (2,208.71 to 3,621.97) | 176,417.64 (134,375.18 to 228,133.31) | 2,890.13 (2,202.97 to 3,732.13) | 0.07 (0.05 to 0.09) |
| Prevalence | Nigeria | 747,591.71 (590,717.75 to 937,784.94) | 3,261.94 (2,576.48 to 4,091.91) | 2,091,183.02 (1,627,439.06 to 2,649,760.63) | 3,473.81 (2,703.09 to 4,401.40) | 0.21 (0.17 to 0.25) |
| Prevalence | Niue | 20.34 (15.68 to 26.40) | 3,976.69 (3,060.84 to 5,161.55) | 14.84 (11.47 to 19.03) | 4,197.62 (3,241.23 to 5,382.91) | 0.17 (0.16 to 0.18) |
| Prevalence | North Macedonia | 14,320.44 (11,372.07 to 17,791.39) | 2,885.64 (2,291.80 to 3,585.15) | 12,834.36 (10,132.49 to 16,049.70) | 2,900.16 (2,292.25 to 3,629.37) | 0.02 (-0.04 to 0.07) |
| Prevalence | Northern Mariana Islands | 593.52 (462.46 to 749.39) | 4,017.48 (3,133.80 to 5,068.42) | 405.02 (315.95 to 515.54) | 4,186.80 (3,264.93 to 5,326.17) | 0.13 (0.05 to 0.21) |
| Prevalence | Norway | 23,577.82 (17,725.99 to 30,486.41) | 2,367.42 (1,779.43 to 3,060.54) | 26,935.69 (20,311.64 to 34,622.49) | 2,519.12 (1,898.68 to 3,240.33) | 0.20 (0.18 to 0.22) |
| Prevalence | Oman | 22,857.24 (17,246.66 to 29,580.60) | 4,362.91 (3,289.88 to 5,660.34) | 60,354.50 (45,318.57 to 77,162.00) | 4,760.59 (3,572.90 to 6,135.40) | 0.28 (0.26 to 0.30) |
| Prevalence | Pakistan | 1,269,842.22 (976,127.01 to 1,605,492.90) | 4,583.79 (3,520.59 to 5,791.94) | 3,172,389.10 (2,457,579.15 to 3,999,449.67) | 4,841.62 (3,750.05 to 6,103.11) | 0.17 (0.14 to 0.21) |
| Prevalence | Palau | 175.01 (135.47 to 224.67) | 3,879.46 (3,003.08 to 4,979.40) | 140.48 (108.47 to 179.83) | 4,113.72 (3,176.81 to 5,265.66) | 0.19 (0.17 to 0.20) |
| Prevalence | Palestine | 26,159.39 (20,042.04 to 33,590.45) | 4,828.75 (3,700.19 to 6,191.18) | 70,051.39 (53,480.84 to 89,894.95) | 4,691.63 (3,582.20 to 6,017.57) | -0.09 (-0.11 to -0.08) |
| Prevalence | Panama | 21,851.38 (16,823.56 to 27,756.10) | 3,215.62 (2,475.28 to 4,082.38) | 35,527.35 (27,173.95 to 45,166.86) | 3,340.43 (2,555.71 to 4,244.66) | 0.12 (0.11 to 0.14) |
| Prevalence | Papua New Guinea | 37,222.93 (28,388.45 to 47,659.16) | 3,313.99 (2,527.42 to 4,240.68) | 99,355.88 (75,593.79 to 127,702.52) | 3,524.18 (2,681.55 to 4,527.87) | 0.19 (0.17 to 0.22) |
| Prevalence | Paraguay | 30,531.09 (23,426.76 to 39,024.28) | 2,954.85 (2,266.30 to 3,775.81) | 59,016.37 (45,340.55 to 75,658.82) | 3,026.96 (2,325.75 to 3,880.14) | 0.08 (0.05 to 0.10) |
| Prevalence | Peru | 158,369.48 (124,000.39 to 198,242.17) | 2,649.86 (2,071.75 to 3,315.37) | 259,836.57 (197,527.63 to 334,697.82) | 2,717.26 (2,067.24 to 3,499.99) | 0.08 (0.07 to 0.10) |
| Prevalence | Philippines | 913,858.41 (696,313.07 to 1,176,369.63) | 5,296.86 (4,036.07 to 6,813.34) | 1,632,030.45 (1,250,162.62 to 2,087,566.64) | 5,286.07 (4,049.41 to 6,760.26) | -0.01 (-0.02 to 0.00) |
| Prevalence | Poland | 243,842.09 (193,018.83 to 303,353.38) | 3,070.21 (2,430.22 to 3,821.17) | 182,875.75 (141,084.38 to 233,140.33) | 2,814.51 (2,174.48 to 3,589.97) | -0.28 (-0.32 to -0.23) |
| Prevalence | Portugal | 55,362.04 (42,158.99 to 71,338.47) | 2,324.89 (1,769.93 to 2,995.62) | 38,390.79 (28,984.87 to 49,958.49) | 2,223.78 (1,678.76 to 2,894.02) | -0.14 (-0.16 to -0.13) |
| Prevalence | Puerto Rico | 27,370.44 (20,839.39 to 34,853.35) | 3,063.54 (2,331.32 to 3,900.57) | 20,415.30 (15,594.18 to 25,988.31) | 3,200.15 (2,445.25 to 4,072.51) | 0.14 (0.13 to 0.15) |
| Prevalence | Qatar | 6,560.93 (4,947.38 to 8,394.88) | 4,833.88 (3,645.93 to 6,212.45) | 40,464.63 (30,483.86 to 52,246.42) | 4,769.70 (3,586.35 to 6,218.88) | -0.04 (-0.07 to 0.00) |
| Prevalence | Republic of Korea | 417,379.04 (315,135.79 to 539,707.12) | 3,065.10 (2,314.82 to 3,963.31) | 277,782.98 (207,588.84 to 362,297.44) | 2,849.85 (2,131.22 to 3,720.44) | -0.23 (-0.34 to -0.12) |
| Prevalence | Republic of Moldova | 46,421.31 (35,492.07 to 59,395.44) | 4,477.56 (3,423.61 to 5,734.39) | 30,565.00 (23,525.40 to 39,145.44) | 4,665.72 (3,591.31 to 5,981.19) | 0.13 (0.11 to 0.15) |
| Prevalence | Romania | 121,285.32 (94,702.20 to 152,648.06) | 2,431.35 (1,899.03 to 3,055.28) | 68,958.40 (53,431.24 to 87,964.40) | 2,327.76 (1,803.68 to 2,969.20) | -0.14 (-0.18 to -0.10) |
| Prevalence | Russian Federation | 1,510,393.43 (1,162,738.32 to 1,927,963.96) | 4,385.17 (3,376.15 to 5,604.72) | 1,001,189.49 (760,772.40 to 1,291,495.18) | 4,352.54 (3,306.38 to 5,620.90) | -0.03 (-0.04 to -0.02) |
| Prevalence | Rwanda | 53,970.47 (41,030.01 to 69,689.26) | 3,002.87 (2,281.51 to 3,878.50) | 106,691.62 (79,588.51 to 138,564.51) | 2,926.02 (2,181.12 to 3,794.91) | -0.09 (-0.12 to -0.05) |
| Prevalence | Saint Kitts and Nevis | 363.91 (284.78 to 461.61) | 3,228.58 (2,525.53 to 4,094.76) | 446.66 (344.26 to 565.90) | 3,281.26 (2,530.97 to 4,157.21) | 0.05 (0.03 to 0.07) |
| Prevalence | Saint Lucia | 1,224.61 (953.70 to 1,561.16) | 3,171.57 (2,469.58 to 4,040.82) | 1,343.27 (1,043.81 to 1,702.92) | 3,255.20 (2,530.04 to 4,127.47) | 0.08 (0.04 to 0.13) |
| Prevalence | Saint Vincent and the Grenadines | 938.79 (728.61 to 1,193.49) | 2,991.76 (2,321.11 to 3,803.13) | 833.85 (638.97 to 1,066.96) | 3,189.19 (2,444.54 to 4,078.94) | 0.20 (0.17 to 0.24) |
| Prevalence | Samoa | 1,731.15 (1,340.60 to 2,219.84) | 3,762.86 (2,911.52 to 4,828.43) | 2,090.25 (1,610.87 to 2,678.40) | 3,994.60 (3,077.76 to 5,116.51) | 0.19 (0.17 to 0.21) |
| Prevalence | San Marino | 138.52 (104.68 to 177.53) | 2,251.06 (1,701.62 to 2,884.81) | 123.10 (93.29 to 159.68) | 2,249.27 (1,704.53 to 2,917.75) | 0.00 (-0.02 to 0.03) |
| Prevalence | Sao Tome and Principe | 1,038.38 (809.88 to 1,316.28) | 3,501.80 (2,727.91 to 4,435.62) | 2,122.27 (1,644.76 to 2,694.19) | 3,651.40 (2,829.79 to 4,634.65) | 0.14 (0.13 to 0.16) |
| Prevalence | Saudi Arabia | 206,102.16 (154,795.93 to 267,572.18) | 4,670.26 (3,507.55 to 6,056.68) | 489,110.84 (372,370.76 to 628,533.99) | 4,706.91 (3,577.12 to 6,069.04) | 0.03 (0.01 to 0.04) |
| Prevalence | Senegal | 61,350.99 (46,943.95 to 79,016.74) | 3,289.43 (2,514.93 to 4,236.55) | 137,954.85 (104,463.95 to 179,775.67) | 3,205.43 (2,426.73 to 4,176.22) | -0.08 (-0.11 to -0.06) |
| Prevalence | Serbia | 60,067.92 (45,678.73 to 76,895.79) | 2,774.73 (2,109.80 to 3,553.99) | 47,178.21 (35,813.08 to 61,324.33) | 2,731.57 (2,073.40 to 3,555.72) | -0.07 (-0.09 to -0.05) |
| Prevalence | Seychelles | 1,143.50 (856.44 to 1,471.21) | 5,385.11 (4,033.10 to 6,928.07) | 1,269.96 (949.19 to 1,633.82) | 5,574.93 (4,165.15 to 7,176.60) | 0.11 (0.10 to 0.11) |
| Prevalence | Sierra Leone | 35,309.51 (27,088.39 to 45,166.89) | 3,284.32 (2,519.87 to 4,199.88) | 84,901.06 (64,639.52 to 109,247.29) | 3,363.96 (2,559.89 to 4,323.67) | 0.09 (0.07 to 0.11) |
| Prevalence | Singapore | 42,953.10 (32,195.86 to 55,435.61) | 4,428.82 (3,320.40 to 5,719.10) | 41,469.56 (31,352.37 to 53,432.96) | 4,273.71 (3,238.11 to 5,512.43) | -0.11 (-0.15 to -0.07) |
| Prevalence | Slovakia | 32,421.54 (25,336.53 to 40,632.67) | 2,709.87 (2,117.98 to 3,397.70) | 24,418.15 (18,789.76 to 31,182.59) | 2,609.16 (2,009.29 to 3,335.31) | -0.12 (-0.16 to -0.09) |
| Prevalence | Slovenia | 12,287.47 (9,386.20 to 15,728.03) | 2,674.44 (2,043.77 to 3,423.68) | 8,167.50 (6,221.95 to 10,456.57) | 2,670.91 (2,035.26 to 3,423.00) | -0.01 (-0.03 to 0.00) |
| Prevalence | Solomon Islands | 3,219.49 (2,512.91 to 4,106.56) | 3,697.57 (2,887.70 to 4,712.60) | 6,842.44 (5,325.32 to 8,680.11) | 3,850.45 (2,994.65 to 4,880.83) | 0.13 (0.10 to 0.16) |
| Prevalence | Somalia | 49,412.70 (37,608.24 to 63,698.90) | 2,758.66 (2,097.84 to 3,556.12) | 156,649.54 (117,445.94 to 203,474.50) | 2,808.72 (2,105.95 to 3,644.47) | 0.06 (0.05 to 0.07) |
| Prevalence | South Africa | 368,385.38 (285,094.91 to 469,386.10) | 3,478.54 (2,692.41 to 4,430.81) | 502,663.16 (385,501.28 to 638,613.97) | 3,423.29 (2,624.93 to 4,353.80) | -0.05 (-0.07 to -0.03) |
| Prevalence | South Sudan | 45,867.34 (34,691.27 to 59,872.75) | 2,879.05 (2,175.08 to 3,754.87) | 65,497.99 (48,711.20 to 85,003.78) | 2,962.15 (2,200.61 to 3,838.33) | 0.10 (0.07 to 0.13) |
| Prevalence | Spain | 189,640.86 (144,987.56 to 240,672.08) | 1,973.98 (1,509.24 to 2,504.77) | 136,934.57 (103,086.59 to 175,658.64) | 1,972.09 (1,485.17 to 2,530.27) | -0.01 (-0.02 to 0.01) |
| Prevalence | Sri Lanka | 251,821.40 (189,948.96 to 324,632.83) | 5,240.98 (3,953.40 to 6,755.01) | 268,207.95 (199,598.59 to 349,027.99) | 5,403.35 (4,021.05 to 7,032.70) | 0.10 (0.08 to 0.11) |
| Prevalence | Sudan | 234,889.58 (176,810.65 to 305,619.51) | 4,596.80 (3,464.09 to 5,963.83) | 601,696.28 (448,471.18 to 787,334.95) | 4,896.20 (3,650.94 to 6,401.11) | 0.20 (0.19 to 0.21) |
| Prevalence | Suriname | 3,456.10 (2,701.05 to 4,376.94) | 3,031.14 (2,368.97 to 3,835.75) | 4,189.96 (3,282.35 to 5,279.26) | 3,160.51 (2,476.10 to 3,979.56) | 0.13 (0.11 to 0.16) |
| Prevalence | Sweden | 54,841.07 (41,001.16 to 71,043.68) | 3,000.07 (2,242.21 to 3,887.60) | 57,916.02 (43,490.00 to 74,683.78) | 3,055.20 (2,296.41 to 3,945.59) | 0.06 (0.04 to 0.08) |
| Prevalence | Switzerland | 44,517.45 (33,623.99 to 57,367.83) | 2,664.00 (2,013.44 to 3,437.04) | 41,295.78 (31,077.24 to 53,630.34) | 2,605.45 (1,962.94 to 3,387.18) | -0.07 (-0.08 to -0.06) |
| Prevalence | Syrian Arab Republic | 160,415.52 (123,812.24 to 205,553.44) | 4,867.20 (3,753.55 to 6,223.71) | 138,072.16 (104,611.45 to 180,077.63) | 4,773.61 (3,608.85 to 6,155.82) | -0.06 (-0.08 to -0.04) |
| Prevalence | Taiwan (Province of China) | 160,260.56 (124,477.14 to 203,952.73) | 2,734.71 (2,125.48 to 3,479.62) | 118,032.38 (90,667.91 to 151,732.81) | 2,614.49 (2,009.78 to 3,361.36) | -0.14 (-0.16 to -0.13) |
| Prevalence | Tajikistan | 60,408.29 (47,189.07 to 76,309.59) | 4,105.26 (3,206.18 to 5,187.23) | 109,203.56 (85,026.57 to 139,569.47) | 4,078.07 (3,175.61 to 5,212.30) | -0.02 (-0.03 to 0.00) |
| Prevalence | Thailand | 878,344.69 (674,661.68 to 1,122,001.33) | 5,122.88 (3,934.15 to 6,541.18) | 652,566.80 (489,923.32 to 844,327.22) | 5,093.40 (3,823.12 to 6,593.10) | -0.02 (-0.04 to 0.00) |
| Prevalence | Timor-Leste | 10,848.24 (8,228.76 to 13,988.03) | 5,002.62 (3,794.61 to 6,447.68) | 21,007.88 (15,579.66 to 27,411.33) | 5,320.11 (3,945.08 to 6,927.93) | 0.20 (0.18 to 0.22) |
| Prevalence | Togo | 29,455.22 (22,508.83 to 37,687.52) | 3,150.11 (2,406.84 to 4,027.29) | 69,093.24 (52,453.90 to 88,329.54) | 3,163.26 (2,400.84 to 4,040.53) | 0.02 (0.00 to 0.04) |
| Prevalence | Tokelau | 13.60 (10.43 to 17.37) | 3,647.85 (2,794.49 to 4,656.43) | 12.28 (9.37 to 15.93) | 3,910.51 (2,983.54 to 5,071.72) | 0.22 (0.20 to 0.25) |
| Prevalence | Tonga | 965.73 (739.66 to 1,237.22) | 3,840.22 (2,936.29 to 4,914.77) | 1,030.06 (785.35 to 1,320.71) | 4,063.20 (3,097.11 to 5,203.16) | 0.18 (0.17 to 0.19) |
| Prevalence | Trinidad and Tobago | 10,072.18 (7,767.21 to 12,840.41) | 3,047.23 (2,350.25 to 3,886.82) | 8,895.04 (6,836.92 to 11,394.80) | 3,246.31 (2,495.23 to 4,161.74) | 0.20 (0.18 to 0.23) |
| Prevalence | Tunisia | 106,362.32 (79,675.65 to 136,592.07) | 4,557.85 (3,414.94 to 5,847.68) | 115,328.16 (87,170.73 to 149,474.39) | 4,608.28 (3,481.65 to 5,980.90) | 0.04 (0.02 to 0.05) |
| Prevalence | Turkey | 826,430.54 (629,953.11 to 1,071,646.54) | 5,236.94 (3,994.84 to 6,781.36) | 1,002,692.95 (747,647.02 to 1,300,647.00) | 5,111.22 (3,808.58 to 6,634.40) | -0.06 (-0.12 to 0.00) |
| Prevalence | Turkmenistan | 48,003.41 (38,010.14 to 60,126.11) | 4,586.45 (3,630.35 to 5,747.75) | 61,205.77 (48,351.49 to 76,085.86) | 4,633.18 (3,661.57 to 5,758.95) | 0.04 (0.00 to 0.08) |
| Prevalence | Tuvalu | 86.48 (66.86 to 110.08) | 3,740.24 (2,891.98 to 4,758.17) | 128.72 (99.18 to 164.09) | 3,971.04 (3,059.24 to 5,059.94) | 0.19 (0.17 to 0.21) |
| Prevalence | Uganda | 135,970.34 (103,222.21 to 175,341.58) | 3,042.61 (2,309.14 to 3,919.13) | 357,266.15 (268,011.71 to 464,811.08) | 3,054.71 (2,292.48 to 3,971.84) | 0.01 (0.00 to 0.03) |
| Prevalence | Ukraine | 493,684.07 (372,641.50 to 633,746.80) | 4,334.10 (3,270.70 to 5,569.88) | 310,703.46 (233,781.94 to 399,922.60) | 4,359.31 (3,283.92 to 5,623.71) | 0.02 (0.01 to 0.02) |
| Prevalence | United Arab Emirates | 28,875.71 (21,830.42 to 37,081.45) | 5,097.71 (3,848.59 to 6,579.80) | 47,972.43 (36,616.01 to 61,441.61) | 4,916.85 (3,754.52 to 6,288.06) | -0.12 (-0.14 to -0.10) |
| Prevalence | United Kingdom | 291,057.13 (225,529.58 to 367,808.27) | 2,140.07 (1,659.96 to 2,704.99) | 259,220.56 (199,395.52 to 328,190.44) | 2,004.06 (1,542.62 to 2,538.82) | -0.22 (-0.26 to -0.18) |
| Prevalence | United Republic of Tanzania | 186,484.30 (141,796.09 to 240,789.64) | 2,840.16 (2,156.48 to 3,662.62) | 492,846.87 (371,955.57 to 635,379.23) | 3,172.60 (2,394.00 to 4,085.12) | 0.35 (0.33 to 0.38) |
| Prevalence | United States Virgin Islands | 1,883,229.54 (1,443,279.92 to 2,402,012.85) | 3,026.01 (2,321.06 to 3,862.05) | 1,977,606.32 (1,512,012.56 to 2,524,069.26) | 2,926.67 (2,238.42 to 3,736.32) | -0.10 (-0.15 to -0.05) |
| Prevalence | United States of America | 705.54 (540.40 to 898.82) | 2,979.60 (2,279.83 to 3,796.15) | 413.93 (313.95 to 532.31) | 3,072.79 (2,329.40 to 3,956.92) | 0.09 (0.06 to 0.13) |
| Prevalence | Uruguay | 20,442.91 (15,510.58 to 26,160.25) | 2,862.78 (2,171.81 to 3,663.34) | 22,338.56 (16,882.12 to 28,717.65) | 2,987.59 (2,258.20 to 3,841.52) | 0.14 (0.13 to 0.15) |
| Prevalence | Uzbekistan | 259,083.98 (207,899.01 to 320,559.83) | 4,430.94 (3,554.82 to 5,484.49) | 404,956.21 (323,755.61 to 496,978.29) | 4,782.83 (3,822.67 to 5,868.63) | 0.25 (0.23 to 0.26) |
| Prevalence | Vanuatu | 1,527.27 (1,159.64 to 1,968.97) | 3,906.93 (2,967.37 to 5,033.84) | 3,425.80 (2,616.33 to 4,384.28) | 4,141.94 (3,163.39 to 5,297.43) | 0.19 (0.18 to 0.20) |
| Prevalence | Venezuela (Bolivarian Republic of) | 170,455.87 (130,420.21 to 217,664.64) | 3,230.12 (2,471.10 to 4,122.60) | 162,156.80 (124,256.76 to 206,964.54) | 3,132.94 (2,399.75 to 4,000.67) | -0.10 (-0.13 to -0.07) |
| Prevalence | Viet Nam | 957,192.49 (720,103.09 to 1,242,784.08) | 4,973.20 (3,740.87 to 6,455.23) | 1,153,486.25 (858,360.13 to 1,497,748.51) | 5,162.80 (3,838.45 to 6,713.00) | 0.12 (0.11 to 0.14) |
| Prevalence | Yemen | 124,979.60 (94,616.40 to 162,256.98) | 4,205.93 (3,186.93 to 5,447.85) | 380,520.71 (284,508.80 to 499,171.70) | 4,408.23 (3,298.45 to 5,770.88) | 0.15 (0.13 to 0.17) |
| Prevalence | Zambia | 58,685.39 (44,770.34 to 75,424.12) | 2,804.85 (2,136.02 to 3,598.58) | 161,500.33 (122,184.01 to 208,966.27) | 2,968.73 (2,245.31 to 3,838.72) | 0.18 (0.17 to 0.20) |
| Prevalence | Zimbabwe | 83,994.20 (63,805.12 to 108,006.19) | 3,160.39 (2,401.57 to 4,059.85) | 136,750.87 (104,001.14 to 177,112.89) | 3,310.42 (2,520.93 to 4,284.20) | 0.16 (0.14 to 0.18) |
