# Supplementary material for: Global, regional, and national burden of chronic kidney disease among adolescents and emerging adults from 1990 to 2021
Source: Ren Fail. 2025 May 22;47(1):2508296. doi: 10.1080/0886022X.2025.2508296 (PMC12101043; doi:10.1080/0886022X.2025.2508296)
Supplement: Supplementary Table S4.docx [file IRNF_A_2508296_SM3055.docx]

Supplementary Table S4 Incidence of CKD among Adolescents and Emerging adults in 204 Countries or Territories from 1990 to 2021 and Their AAPC.

| **Measure** | **location** | 1990 | | 2021 | | AAPC (95% UI) |
| --- | --- | --- | --- | --- | --- | --- |
|  |  | Number (95% UI) | ASR (95% UI) | Number (95% UI) | ASR (95% UI) |  |
| Incidence | Afghanistan | 667.59 (319.65 to 1,073.21) | 28.15 (13.25 to 45.72) | 3,592.77 (1,944.18 to 5,453.98) | 39.31 (21.16 to 59.89) | 1.07 (0.99 to 1.14) |
| Incidence | Albania | 170.61 (65.70 to 298.01) | 18.10 (6.97 to 31.61) | 202.86 (103.01 to 314.63) | 34.33 (17.49 to 53.16) | 2.10 (2.00 to 2.20) |
| Incidence | Algeria | 1,280.14 (553.43 to 2,190.53) | 17.90 (7.69 to 30.65) | 2,399.62 (1,093.24 to 4,062.98) | 24.39 (11.14 to 41.15) | 1.00 (0.95 to 1.04) |
| Incidence | American Samoa | 5.78 (3.15 to 8.82) | 41.55 (22.57 to 63.45) | 7.31 (4.42 to 10.46) | 65.43 (39.37 to 93.88) | 1.47 (1.43 to 1.52) |
| Incidence | Andorra | 0.81 (0.19 to 1.69) | 5.37 (1.28 to 11.39) | 0.80 (0.18 to 1.71) | 5.78 (1.31 to 12.47) | 0.25 (0.20 to 0.29) |
| Incidence | Angola | 375.78 (159.43 to 636.82) | 13.66 (5.71 to 23.33) | 1,456.45 (664.41 to 2,334.11) | 16.78 (7.48 to 27.19) | 0.66 (0.59 to 0.74) |
| Incidence | Antigua and Barbuda | 4.53 (2.10 to 7.38) | 26.66 (12.36 to 43.44) | 9.34 (5.18 to 14.28) | 45.02 (25.05 to 68.72) | 1.71 (1.64 to 1.78) |
| Incidence | Argentina | 588.26 (142.62 to 1,259.76) | 7.62 (1.86 to 16.30) | 901.21 (267.84 to 1,716.29) | 8.30 (2.45 to 15.82) | 0.30 (0.22 to 0.38) |
| Incidence | Armenia | 258.03 (126.54 to 412.99) | 29.41 (14.61 to 46.90) | 213.36 (119.74 to 326.89) | 38.87 (22.18 to 58.98) | 0.92 (0.89 to 0.96) |
| Incidence | Australia | 258.30 (54.75 to 547.96) | 6.27 (1.33 to 13.29) | 398.14 (98.62 to 799.72) | 7.96 (1.97 to 16.03) | 0.77 (0.67 to 0.87) |
| Incidence | Austria | 116.45 (28.17 to 244.62) | 6.04 (1.42 to 12.70) | 110.65 (27.96 to 232.39) | 6.75 (1.67 to 14.21) | 0.40 (0.31 to 0.49) |
| Incidence | Azerbaijan | 829.14 (450.05 to 1,267.35) | 39.26 (21.44 to 59.84) | 1,256.17 (738.97 to 1,818.56) | 55.95 (33.50 to 79.81) | 1.17 (1.09 to 1.24) |
| Incidence | Bahamas | 22.08 (10.72 to 35.38) | 27.81 (13.50 to 44.58) | 44.10 (25.19 to 66.56) | 45.83 (26.18 to 69.18) | 1.64 (1.59 to 1.70) |
| Incidence | Bahrain | 20.59 (7.48 to 38.05) | 13.49 (4.86 to 24.80) | 78.17 (33.09 to 135.76) | 20.01 (8.48 to 34.66) | 1.28 (1.22 to 1.34) |
| Incidence | Bangladesh | 2,651.50 (872.92 to 4,888.55) | 8.99 (2.96 to 16.63) | 5,841.01 (2,349.66 to 10,068.78) | 13.26 (5.33 to 22.86) | 1.28 (1.15 to 1.42) |
| Incidence | Barbados | 17.21 (7.99 to 28.32) | 25.18 (11.69 to 41.45) | 22.63 (12.42 to 34.35) | 38.54 (21.17 to 58.46) | 1.38 (1.30 to 1.46) |
| Incidence | Belarus | 425.67 (163.17 to 751.22) | 18.27 (7.03 to 32.15) | 428.71 (210.72 to 685.60) | 29.56 (14.68 to 47.12) | 1.56 (1.48 to 1.65) |
| Incidence | Belgium | 134.57 (28.14 to 283.52) | 5.95 (1.23 to 12.60) | 132.42 (31.57 to 278.86) | 6.47 (1.53 to 13.62) | 0.27 (0.22 to 0.32) |
| Incidence | Belize | 14.02 (6.56 to 22.93) | 27.14 (12.68 to 44.45) | 64.22 (37.76 to 95.47) | 51.34 (30.08 to 76.43) | 2.13 (2.02 to 2.24) |
| Incidence | Benin | 297.30 (145.80 to 467.77) | 24.92 (12.11 to 39.32) | 1,193.06 (635.77 to 1,796.37) | 31.81 (16.76 to 48.19) | 0.77 (0.70 to 0.85) |
| Incidence | Bermuda | 2.80 (1.18 to 4.70) | 18.66 (7.70 to 31.31) | 2.71 (1.34 to 4.44) | 28.92 (14.34 to 47.37) | 1.49 (1.43 to 1.56) |
| Incidence | Bhutan | 45.73 (21.77 to 72.00) | 23.35 (11.00 to 36.94) | 70.36 (37.27 to 107.34) | 32.84 (17.49 to 49.93) | 1.11 (1.08 to 1.14) |
| Incidence | Bolivia (Plurinational State of) | 267.95 (107.95 to 459.06) | 15.78 (6.33 to 27.08) | 629.82 (287.98 to 1,074.76) | 20.00 (9.18 to 34.09) | 0.77 (0.68 to 0.86) |
| Incidence | Bosnia and Herzegovina | 180.38 (66.20 to 318.10) | 15.26 (5.59 to 26.91) | 138.23 (64.40 to 225.68) | 24.04 (11.21 to 39.20) | 1.50 (1.38 to 1.63) |
| Incidence | Botswana | 78.07 (36.56 to 126.24) | 22.10 (10.50 to 35.67) | 177.92 (88.12 to 284.33) | 27.45 (13.61 to 43.87) | 0.71 (0.59 to 0.83) |
| Incidence | Brazil | 7,023.07 (2,916.05 to 12,356.87) | 16.88 (7.03 to 29.69) | 8,895.79 (3,758.18 to 15,274.32) | 17.30 (7.26 to 29.72) | 0.08 (0.05 to 0.11) |
| Incidence | Brunei Darussalam | 12.61 (4.57 to 23.29) | 16.03 (5.81 to 29.62) | 23.20 (8.87 to 41.09) | 19.46 (7.43 to 34.46) | 0.64 (0.54 to 0.74) |
| Incidence | Bulgaria | 337.03 (134.39 to 583.37) | 19.07 (7.61 to 33.00) | 388.96 (208.50 to 586.73) | 40.00 (21.52 to 60.25) | 2.41 (2.35 to 2.46) |
| Incidence | Burkina Faso | 454.92 (205.13 to 738.54) | 19.88 (8.84 to 32.48) | 1,470.19 (777.48 to 2,318.75) | 24.21 (12.54 to 38.39) | 0.64 (0.58 to 0.70) |
| Incidence | Burundi | 212.83 (89.79 to 352.00) | 14.79 (6.16 to 24.55) | 450.77 (195.68 to 755.37) | 12.34 (5.24 to 20.85) | -0.59 (-0.65 to -0.54) |
| Incidence | Cabo Verde | 19.65 (9.00 to 32.41) | 20.25 (9.18 to 33.45) | 46.81 (24.12 to 73.30) | 30.96 (16.04 to 48.35) | 1.34 (1.22 to 1.46) |
| Incidence | Cambodia | 594.09 (275.09 to 971.25) | 21.88 (10.07 to 35.82) | 1,125.26 (564.81 to 1,780.99) | 24.92 (12.56 to 39.37) | 0.41 (0.38 to 0.45) |
| Incidence | Cameroon | 1,000.19 (525.04 to 1,557.65) | 36.80 (19.13 to 57.59) | 3,998.66 (2,393.63 to 5,834.79) | 44.65 (26.37 to 65.69) | 0.60 (0.50 to 0.70) |
| Incidence | Canada | 425.56 (100.61 to 894.77) | 6.53 (1.53 to 13.75) | 470.77 (119.77 to 984.72) | 6.88 (1.75 to 14.38) | 0.21 (0.11 to 0.31) |
| Incidence | Central African Republic | 126.62 (57.76 to 210.37) | 17.20 (7.80 to 28.69) | 355.51 (186.18 to 550.97) | 23.19 (11.86 to 36.17) | 0.94 (0.90 to 0.98) |
| Incidence | Chad | 340.43 (168.26 to 534.94) | 22.75 (11.06 to 36.04) | 1,296.27 (697.12 to 1,965.92) | 27.69 (14.57 to 42.43) | 0.63 (0.51 to 0.75) |
| Incidence | Chile | 273.53 (57.28 to 572.28) | 7.24 (1.52 to 15.16) | 380.49 (98.01 to 745.89) | 8.82 (2.25 to 17.34) | 0.64 (0.58 to 0.70) |
| Incidence | China | 50,819.09 (21,004.49 to 89,429.31) | 13.88 (5.76 to 24.43) | 29,442.84 (12,098.61 to 51,532.71) | 12.29 (5.03 to 21.53) | -0.38 (-0.44 to -0.31) |
| Incidence | Colombia | 2,097.17 (946.91 to 3,449.56) | 21.99 (9.93 to 36.19) | 2,894.66 (1,376.13 to 4,734.69) | 22.70 (10.82 to 37.05) | 0.12 (0.07 to 0.17) |
| Incidence | Comoros | 21.77 (10.52 to 36.63) | 16.81 (7.89 to 28.51) | 36.92 (18.17 to 58.27) | 17.90 (8.75 to 28.33) | 0.20 (0.15 to 0.26) |
| Incidence | Congo | 136.31 (64.61 to 221.23) | 19.72 (9.20 to 32.20) | 331.16 (169.30 to 525.38) | 22.58 (11.41 to 35.97) | 0.44 (0.39 to 0.48) |
| Incidence | Cook Islands | 1.49 (0.73 to 2.38) | 28.29 (13.96 to 45.20) | 2.02 (1.17 to 2.93) | 53.00 (30.82 to 76.96) | 2.06 (2.03 to 2.09) |
| Incidence | Costa Rica | 252.20 (119.63 to 410.93) | 29.38 (13.92 to 47.86) | 436.65 (239.77 to 682.67) | 38.38 (21.15 to 59.87) | 0.87 (0.84 to 0.89) |
| Incidence | Coted'Ivoire | 1,023.30 (519.22 to 1,597.46) | 30.58 (15.43 to 47.89) | 2,749.18 (1,582.30 to 4,108.89) | 36.59 (20.90 to 54.97) | 0.58 (0.55 to 0.61) |
| Incidence | Croatia | 139.62 (45.93 to 253.75) | 13.19 (4.31 to 23.99) | 138.05 (59.10 to 231.06) | 19.54 (8.33 to 32.68) | 1.30 (1.25 to 1.35) |
| Incidence | Cuba | 719.30 (302.06 to 1,231.21) | 21.09 (8.85 to 36.09) | 635.08 (318.42 to 1,021.58) | 30.60 (15.37 to 49.24) | 1.22 (1.15 to 1.29) |
| Incidence | Cyprus | 12.18 (2.79 to 26.21) | 6.34 (1.44 to 13.66) | 19.04 (4.45 to 38.90) | 7.34 (1.71 to 14.92) | 0.50 (0.32 to 0.68) |
| Incidence | Czechia | 270.91 (81.60 to 507.00) | 12.47 (3.77 to 23.27) | 284.71 (120.68 to 481.69) | 18.31 (7.75 to 30.99) | 1.24 (1.22 to 1.27) |
| Incidence | Democratic People's Republic of Korea | 1,161.11 (478.95 to 1,971.53) | 20.74 (8.54 to 35.19) | 1,482.26 (683.94 to 2,380.38) | 24.93 (11.55 to 39.92) | 0.58 (0.55 to 0.62) |
| Incidence | Democratic Republic of the Congo | 1,307.84 (543.56 to 2,199.60) | 12.77 (5.23 to 21.61) | 4,074.27 (1,711.22 to 6,695.17) | 15.94 (6.60 to 26.37) | 0.69 (0.60 to 0.78) |
| Incidence | Denmark | 71.48 (17.37 to 153.74) | 6.05 (1.47 to 13.03) | 73.54 (17.52 to 156.17) | 6.41 (1.51 to 13.68) | 0.21 (0.16 to 0.26) |
| Incidence | Djibouti | 16.33 (7.30 to 27.60) | 12.66 (5.51 to 21.67) | 53.63 (25.93 to 85.34) | 16.13 (7.81 to 25.64) | 0.77 (0.70 to 0.84) |
| Incidence | Dominica | 8.00 (4.11 to 12.53) | 39.34 (20.16 to 61.74) | 8.59 (4.85 to 12.75) | 53.40 (30.17 to 79.30) | 0.99 (0.94 to 1.05) |
| Incidence | Dominican Republic | 410.05 (175.54 to 694.55) | 19.15 (8.19 to 32.40) | 1,018.14 (548.88 to 1,567.09) | 35.21 (19.00 to 54.16) | 2.03 (1.97 to 2.09) |
| Incidence | Ecuador | 546.74 (223.12 to 931.44) | 19.16 (7.78 to 32.66) | 1,369.04 (674.40 to 2,200.80) | 29.24 (14.45 to 46.90) | 1.37 (1.35 to 1.39) |
| Incidence | Egypt | 2,513.88 (1,081.78 to 4,340.47) | 16.90 (7.27 to 29.23) | 6,850.19 (3,251.80 to 11,357.27) | 25.58 (12.13 to 42.44) | 1.35 (1.26 to 1.44) |
| Incidence | El Salvador | 444.21 (222.42 to 700.35) | 30.07 (14.99 to 47.61) | 1,250.66 (753.59 to 1,826.92) | 72.02 (43.47 to 105.06) | 2.87 (2.79 to 2.96) |
| Incidence | Equatorial Guinea | 14.05 (5.73 to 23.71) | 12.98 (5.17 to 22.14) | 89.97 (43.56 to 147.81) | 18.24 (8.67 to 30.20) | 1.11 (1.02 to 1.20) |
| Incidence | Eritrea | 158.20 (71.79 to 258.94) | 16.78 (7.44 to 27.66) | 360.75 (181.19 to 571.53) | 18.94 (9.42 to 30.14) | 0.38 (0.32 to 0.44) |
| Incidence | Estonia | 65.28 (24.33 to 113.75) | 19.51 (7.29 to 33.99) | 63.09 (30.44 to 103.43) | 30.93 (14.94 to 50.50) | 1.50 (1.40 to 1.60) |
| Incidence | Eswatini | 62.12 (30.51 to 99.53) | 29.61 (14.66 to 47.47) | 129.33 (69.48 to 198.76) | 38.68 (20.78 to 59.42) | 0.88 (0.72 to 1.05) |
| Incidence | Ethiopia | 1,905.76 (858.29 to 3,170.98) | 14.69 (6.58 to 24.52) | 4,204.38 (1,832.64 to 6,764.86) | 12.69 (5.47 to 20.54) | -0.48 (-0.52 to -0.44) |
| Incidence | Fiji | 77.41 (41.58 to 120.06) | 36.09 (19.37 to 55.99) | 99.45 (60.27 to 149.66) | 44.84 (27.17 to 67.48) | 0.69 (0.59 to 0.80) |
| Incidence | Finland | 44.39 (11.41 to 94.65) | 4.22 (1.07 to 9.05) | 59.37 (17.66 to 117.89) | 6.12 (1.80 to 12.21) | 1.20 (1.15 to 1.24) |
| Incidence | France | 789.70 (180.38 to 1,709.93) | 5.94 (1.35 to 12.87) | 755.26 (184.26 to 1,623.28) | 6.42 (1.57 to 13.79) | 0.26 (0.22 to 0.30) |
| Incidence | Gabon | 45.13 (19.74 to 74.52) | 16.68 (7.21 to 27.68) | 121.20 (61.03 to 191.27) | 23.73 (11.82 to 37.62) | 1.14 (1.03 to 1.25) |
| Incidence | Gambia | 61.77 (29.46 to 99.92) | 22.91 (10.83 to 37.22) | 226.52 (129.01 to 340.29) | 31.86 (17.92 to 48.21) | 1.06 (1.01 to 1.11) |
| Incidence | Georgia | 454.84 (241.69 to 710.57) | 34.88 (18.67 to 54.30) | 295.92 (174.47 to 433.92) | 48.48 (28.91 to 70.64) | 1.08 (1.03 to 1.12) |
| Incidence | Germany | 1,330.45 (314.19 to 2,790.18) | 7.23 (1.66 to 15.11) | 982.67 (231.50 to 2,074.83) | 6.93 (1.63 to 14.63) | -0.16 (-0.30 to -0.03) |
| Incidence | Ghana | 986.75 (520.61 to 1,540.87) | 24.66 (12.89 to 38.74) | 3,266.69 (1,839.45 to 4,887.91) | 34.16 (19.17 to 51.18) | 1.04 (0.98 to 1.10) |
| Incidence | Greece | 165.67 (35.58 to 353.86) | 7.09 (1.52 to 15.16) | 117.51 (31.62 to 235.05) | 7.59 (2.04 to 15.19) | 0.22 (0.19 to 0.25) |
| Incidence | Greenland | 0.92 (0.24 to 1.93) | 5.42 (1.36 to 11.45) | 0.69 (0.18 to 1.42) | 5.73 (1.48 to 11.80) | 0.16 (0.06 to 0.27) |
| Incidence | Grenada | 7.09 (3.64 to 11.30) | 31.64 (16.22 to 50.50) | 14.29 (8.23 to 21.67) | 53.57 (30.94 to 81.09) | 1.72 (1.67 to 1.78) |
| Incidence | Guam | 12.58 (6.20 to 19.62) | 30.21 (14.82 to 47.17) | 17.86 (10.20 to 27.30) | 50.11 (28.60 to 76.59) | 1.69 (1.61 to 1.77) |
| Incidence | Guatemala | 567.72 (272.02 to 918.53) | 27.64 (13.19 to 44.88) | 1,981.97 (1,081.28 to 3,124.65) | 42.44 (23.07 to 67.03) | 1.40 (1.39 to 1.42) |
| Incidence | Guinea | 350.08 (174.72 to 547.83) | 25.16 (12.44 to 39.51) | 1,212.27 (670.74 to 1,805.78) | 33.37 (18.16 to 50.03) | 0.91 (0.86 to 0.96) |
| Incidence | Guinea-Bissau | 92.80 (49.28 to 142.27) | 35.09 (18.38 to 54.10) | 252.06 (152.43 to 367.15) | 43.38 (25.96 to 63.53) | 0.69 (0.65 to 0.72) |
| Incidence | Guyana | 66.48 (31.98 to 105.47) | 28.35 (13.60 to 45.03) | 92.85 (51.94 to 138.43) | 43.70 (24.47 to 65.09) | 1.40 (1.32 to 1.48) |
| Incidence | Haiti | 469.97 (232.64 to 743.67) | 28.84 (14.24 to 45.69) | 1,190.50 (624.69 to 1,856.09) | 34.19 (17.92 to 53.31) | 0.56 (0.48 to 0.64) |
| Incidence | Honduras | 328.64 (158.08 to 520.74) | 27.39 (13.08 to 43.53) | 1,016.78 (546.44 to 1,581.92) | 34.00 (18.22 to 52.97) | 0.71 (0.67 to 0.75) |
| Incidence | Hungary | 245.33 (81.25 to 464.97) | 11.96 (3.97 to 22.66) | 288.75 (120.57 to 493.82) | 18.39 (7.68 to 31.46) | 1.40 (1.37 to 1.43) |
| Incidence | Iceland | 3.60 (0.85 to 7.74) | 5.55 (1.30 to 11.94) | 4.13 (0.92 to 8.81) | 5.73 (1.27 to 12.22) | 0.08 (0.02 to 0.14) |
| Incidence | India | 50,890.00 (24,260.79 to 82,378.77) | 22.37 (10.66 to 36.25) | 92,241.61 (48,235.17 to 141,395.67) | 23.76 (12.41 to 36.44) | 0.18 (0.08 to 0.28) |
| Incidence | Indonesia | 12,717.41 (6,368.42 to 20,311.17) | 24.09 (12.05 to 38.50) | 23,464.17 (13,330.33 to 35,319.99) | 33.72 (19.24 to 50.69) | 1.08 (1.04 to 1.12) |
| Incidence | Iran (Islamic Republic of) | 3,094.34 (1,339.94 to 5,348.42) | 20.68 (9.01 to 35.74) | 4,402.65 (2,006.42 to 7,466.50) | 24.56 (11.13 to 41.65) | 0.54 (0.42 to 0.66) |
| Incidence | Iraq | 1,194.63 (517.55 to 2,003.93) | 23.54 (10.11 to 39.60) | 3,883.20 (1,944.29 to 6,332.40) | 33.57 (16.79 to 54.78) | 1.15 (1.10 to 1.21) |
| Incidence | Ireland | 83.56 (23.39 to 159.30) | 9.65 (2.71 to 18.40) | 92.96 (30.08 to 166.68) | 10.35 (3.34 to 18.56) | 0.23 (0.20 to 0.26) |
| Incidence | Israel | 80.51 (15.24 to 166.80) | 6.74 (1.29 to 13.98) | 162.16 (40.66 to 334.26) | 7.87 (1.98 to 16.22) | 0.51 (0.47 to 0.55) |
| Incidence | Italy | 854.26 (240.34 to 1,794.97) | 6.25 (1.75 to 13.14) | 505.43 (145.76 to 1,059.56) | 5.57 (1.60 to 11.69) | -0.35 (-0.42 to -0.29) |
| Incidence | Jamaica | 188.18 (84.08 to 311.91) | 27.47 (12.29 to 45.55) | 318.69 (172.79 to 497.33) | 41.72 (22.64 to 65.08) | 1.35 (1.28 to 1.42) |
| Incidence | Japan | 3,227.22 (1,118.44 to 6,094.51) | 11.84 (4.12 to 22.34) | 2,177.40 (772.13 to 4,041.74) | 11.72 (4.14 to 21.77) | -0.21 (-0.42 to 0.01) |
| Incidence | Jordan | 213.56 (92.99 to 362.66) | 18.76 (8.15 to 32.10) | 912.15 (401.37 to 1,532.49) | 25.93 (11.41 to 43.61) | 1.05 (1.01 to 1.10) |
| Incidence | Kazakhstan | 1,854.80 (1,025.96 to 2,815.99) | 43.82 (24.40 to 66.23) | 1,973.48 (1,143.18 to 2,970.67) | 51.08 (29.87 to 76.38) | 0.52 (0.44 to 0.61) |
| Incidence | Kenya | 694.42 (288.75 to 1,198.88) | 10.80 (4.40 to 18.75) | 2,357.75 (1,182.98 to 3,716.68) | 15.66 (7.71 to 24.89) | 1.19 (1.12 to 1.27) |
| Incidence | Kiribati | 12.71 (7.57 to 18.64) | 60.39 (35.88 to 88.67) | 26.64 (16.54 to 37.45) | 82.84 (51.44 to 116.45) | 1.03 (0.96 to 1.11) |
| Incidence | Kuwait | 104.27 (43.94 to 178.28) | 20.52 (8.69 to 34.87) | 235.59 (99.84 to 395.18) | 23.46 (9.95 to 39.31) | 0.43 (0.40 to 0.47) |
| Incidence | Kyrgyzstan | 790.91 (497.96 to 1,117.14) | 65.64 (41.30 to 92.76) | 1,113.52 (683.06 to 1,594.45) | 67.02 (41.46 to 95.49) | 0.08 (0.01 to 0.15) |
| Incidence | Lao People's Democratic Republic | 325.92 (160.27 to 514.00) | 30.52 (14.91 to 48.26) | 684.61 (356.96 to 1,062.17) | 33.41 (17.44 to 51.79) | 0.30 (0.25 to 0.34) |
| Incidence | Latvia | 105.13 (39.94 to 181.24) | 18.31 (6.95 to 31.56) | 84.75 (41.96 to 136.31) | 30.48 (15.20 to 48.82) | 1.66 (1.58 to 1.73) |
| Incidence | Lebanon | 136.97 (57.66 to 239.26) | 17.34 (7.30 to 30.34) | 319.47 (139.30 to 546.12) | 24.13 (10.54 to 41.00) | 1.06 (0.97 to 1.15) |
| Incidence | Lesotho | 73.47 (33.37 to 119.66) | 20.69 (9.59 to 33.60) | 195.04 (105.02 to 295.11) | 35.35 (19.10 to 53.42) | 1.75 (1.70 to 1.80) |
| Incidence | Liberia | 150.76 (72.96 to 242.21) | 23.78 (11.45 to 38.26) | 500.53 (281.18 to 756.17) | 32.43 (17.89 to 49.29) | 0.99 (0.90 to 1.08) |
| Incidence | Libya | 215.84 (89.21 to 372.61) | 18.32 (7.64 to 31.73) | 524.06 (246.88 to 869.39) | 29.75 (14.02 to 49.29) | 1.57 (1.48 to 1.65) |
| Incidence | Lithuania | 170.45 (65.55 to 297.33) | 19.63 (7.56 to 34.20) | 129.39 (63.00 to 206.07) | 27.92 (13.69 to 44.29) | 1.15 (1.05 to 1.26) |
| Incidence | Luxembourg | 5.45 (1.38 to 11.41) | 6.22 (1.54 to 13.10) | 8.76 (2.13 to 17.55) | 7.14 (1.71 to 14.30) | 0.46 (0.39 to 0.53) |
| Incidence | Madagascar | 433.46 (187.85 to 727.76) | 13.35 (5.65 to 22.57) | 1,303.96 (585.02 to 2,043.30) | 15.57 (6.91 to 24.58) | 0.43 (0.33 to 0.52) |
| Incidence | Malawi | 321.21 (131.31 to 565.53) | 11.75 (4.72 to 20.80) | 902.05 (422.02 to 1,445.15) | 14.75 (6.60 to 24.07) | 0.74 (0.66 to 0.82) |
| Incidence | Malaysia | 821.13 (321.92 to 1,419.87) | 16.77 (6.57 to 29.03) | 1,836.93 (851.47 to 3,088.16) | 21.62 (10.05 to 36.20) | 0.83 (0.76 to 0.89) |
| Incidence | Maldives | 12.58 (5.49 to 21.19) | 21.13 (9.18 to 35.68) | 23.86 (9.96 to 40.02) | 19.06 (7.96 to 31.80) | -0.30 (-0.44 to -0.17) |
| Incidence | Mali | 499.91 (244.48 to 800.95) | 23.97 (11.60 to 38.60) | 1,765.10 (875.20 to 2,754.70) | 26.98 (13.14 to 42.40) | 0.37 (0.35 to 0.39) |
| Incidence | Malta | 4.70 (1.09 to 10.20) | 5.91 (1.37 to 12.82) | 5.50 (1.51 to 11.27) | 7.51 (2.01 to 15.42) | 0.79 (0.76 to 0.82) |
| Incidence | Marshall Islands | 5.22 (3.01 to 7.81) | 45.25 (26.06 to 67.69) | 9.33 (5.62 to 13.69) | 62.26 (37.39 to 91.36) | 1.03 (0.98 to 1.07) |
| Incidence | Mauritania | 157.45 (80.26 to 246.05) | 29.15 (14.71 to 45.78) | 389.99 (209.96 to 594.79) | 32.20 (17.11 to 49.46) | 0.31 (0.27 to 0.35) |
| Incidence | Mauritius | 101.91 (49.50 to 169.21) | 32.02 (15.61 to 53.01) | 150.48 (86.19 to 222.14) | 54.00 (31.17 to 79.48) | 1.70 (1.66 to 1.75) |
| Incidence | Mexico | 7,000.29 (3,345.22 to 11,375.81) | 28.27 (13.52 to 45.94) | 18,414.97 (11,726.98 to 26,169.89) | 57.05 (36.33 to 81.05) | 2.29 (2.24 to 2.34) |
| Incidence | Micronesia (Federated States of) | 13.92 (7.88 to 21.11) | 52.64 (29.84 to 79.69) | 23.11 (14.65 to 32.67) | 80.88 (51.19 to 114.49) | 1.41 (1.32 to 1.49) |
| Incidence | Monaco | 0.30 (0.07 to 0.66) | 5.74 (1.27 to 12.53) | 0.35 (0.08 to 0.73) | 6.55 (1.53 to 13.52) | 0.43 (0.39 to 0.46) |
| Incidence | Mongolia | 346.69 (198.66 to 520.40) | 54.57 (31.14 to 82.15) | 454.75 (290.92 to 655.71) | 64.83 (41.85 to 92.88) | 0.58 (0.49 to 0.66) |
| Incidence | Montenegro | 37.44 (16.10 to 62.28) | 23.98 (10.30 to 39.89) | 41.93 (21.69 to 64.81) | 34.77 (18.00 to 53.72) | 1.20 (1.16 to 1.24) |
| Incidence | Morocco | 1,050.30 (435.33 to 1,833.82) | 14.72 (6.11 to 25.73) | 2,433.29 (1,162.23 to 4,015.45) | 26.90 (12.86 to 44.38) | 1.97 (1.90 to 2.03) |
| Incidence | Mozambique | 373.45 (140.51 to 641.27) | 11.11 (4.02 to 19.37) | 1,504.48 (798.89 to 2,322.11) | 16.65 (8.59 to 26.12) | 1.31 (1.25 to 1.38) |
| Incidence | Myanmar | 4,242.22 (2,115.96 to 6,741.33) | 35.93 (17.83 to 57.17) | 6,219.27 (3,580.26 to 9,230.40) | 42.85 (24.58 to 63.71) | 0.58 (0.55 to 0.60) |
| Incidence | Namibia | 79.15 (36.76 to 128.60) | 20.41 (9.54 to 33.05) | 140.31 (65.34 to 232.01) | 20.37 (9.50 to 33.68) | -0.03 (-0.21 to 0.15) |
| Incidence | Nauru | 1.37 (0.79 to 2.07) | 51.83 (29.77 to 78.55) | 2.15 (1.26 to 3.14) | 70.10 (41.09 to 102.54) | 0.98 (0.90 to 1.06) |
| Incidence | Nepal | 1,009.43 (445.16 to 1,666.36) | 20.19 (8.87 to 33.43) | 3,431.36 (1,818.52 to 5,397.35) | 37.80 (19.95 to 59.59) | 2.04 (1.96 to 2.11) |
| Incidence | Netherlands | 172.74 (39.42 to 376.87) | 4.61 (1.04 to 10.13) | 163.82 (38.30 to 357.80) | 5.09 (1.18 to 11.14) | 0.33 (0.28 to 0.38) |
| Incidence | New Zealand | 77.30 (24.67 to 155.10) | 8.95 (2.85 to 17.96) | 125.11 (47.78 to 226.17) | 11.35 (4.28 to 20.58) | 0.76 (0.69 to 0.84) |
| Incidence | Nicaragua | 438.33 (231.41 to 685.91) | 42.04 (22.20 to 65.95) | 1,474.68 (922.08 to 2,107.18) | 80.74 (50.51 to 115.36) | 2.12 (2.04 to 2.20) |
| Incidence | Niger | 461.85 (231.89 to 738.27) | 23.31 (11.51 to 37.48) | 1,795.03 (965.29 to 2,748.77) | 26.58 (13.97 to 41.27) | 0.41 (0.34 to 0.47) |
| Incidence | Nigeria | 5,089.95 (2,478.89 to 8,236.97) | 21.26 (10.32 to 34.46) | 15,320.39 (7,814.83 to 24,245.97) | 24.05 (12.18 to 38.23) | 0.36 (0.28 to 0.45) |
| Incidence | Niue | 0.20 (0.10 to 0.30) | 37.13 (19.85 to 57.27) | 0.19 (0.11 to 0.28) | 53.22 (31.27 to 77.84) | 1.19 (1.12 to 1.27) |
| Incidence | North Macedonia | 77.12 (29.28 to 137.21) | 15.58 (5.91 to 27.72) | 117.16 (53.97 to 189.96) | 27.42 (12.65 to 44.42) | 1.86 (1.81 to 1.92) |
| Incidence | Northern Mariana Islands | 7.97 (4.31 to 11.98) | 55.31 (29.91 to 83.27) | 6.78 (4.07 to 9.81) | 68.61 (41.04 to 99.47) | 0.68 (0.39 to 0.98) |
| Incidence | Norway | 50.92 (13.53 to 105.57) | 5.18 (1.37 to 10.77) | 54.06 (15.60 to 114.11) | 5.17 (1.48 to 10.92) | -0.02 (-0.08 to 0.05) |
| Incidence | Oman | 56.98 (21.07 to 106.17) | 11.21 (4.16 to 20.82) | 208.41 (83.88 to 367.22) | 18.25 (7.45 to 32.02) | 1.57 (1.44 to 1.70) |
| Incidence | Pakistan | 7,688.63 (3,983.02 to 11,965.63) | 26.71 (13.77 to 41.66) | 26,564.17 (15,266.35 to 39,254.07) | 40.19 (23.06 to 59.44) | 1.34 (1.31 to 1.37) |
| Incidence | Palau | 2.34 (1.28 to 3.60) | 51.61 (28.25 to 79.51) | 2.91 (1.71 to 4.27) | 85.45 (50.24 to 125.46) | 1.63 (1.58 to 1.68) |
| Incidence | Palestine | 129.26 (58.30 to 220.78) | 22.87 (10.26 to 39.09) | 405.36 (184.09 to 675.14) | 26.92 (12.21 to 44.89) | 0.52 (0.48 to 0.56) |
| Incidence | Panama | 181.13 (86.71 to 291.76) | 26.23 (12.53 to 42.30) | 383.25 (207.04 to 584.11) | 36.29 (19.58 to 55.29) | 1.06 (1.03 to 1.09) |
| Incidence | Papua New Guinea | 295.25 (148.75 to 463.65) | 25.88 (13.02 to 40.63) | 905.19 (504.01 to 1,362.48) | 32.06 (17.86 to 48.26) | 0.70 (0.65 to 0.76) |
| Incidence | Paraguay | 159.10 (57.62 to 287.51) | 15.20 (5.54 to 27.43) | 433.37 (186.17 to 740.18) | 22.32 (9.59 to 38.11) | 1.25 (1.18 to 1.32) |
| Incidence | Peru | 794.53 (291.88 to 1,431.34) | 12.90 (4.69 to 23.32) | 1,883.45 (821.70 to 3,217.10) | 20.27 (8.89 to 34.56) | 1.51 (1.43 to 1.59) |
| Incidence | Philippines | 3,962.35 (1,857.19 to 6,459.53) | 22.36 (10.49 to 36.49) | 13,404.14 (8,063.96 to 19,412.38) | 43.37 (26.08 to 62.82) | 2.15 (2.07 to 2.23) |
| Incidence | Poland | 1,153.56 (404.46 to 2,158.70) | 14.50 (5.09 to 27.13) | 798.96 (285.29 to 1,453.99) | 12.52 (4.39 to 22.94) | -0.48 (-0.55 to -0.41) |
| Incidence | Portugal | 139.66 (27.78 to 306.03) | 5.84 (1.16 to 12.80) | 100.23 (22.23 to 218.37) | 5.86 (1.30 to 12.77) | 0.03 (-0.06 to 0.11) |
| Incidence | Puerto Rico | 193.50 (83.51 to 326.00) | 21.47 (9.27 to 36.17) | 197.91 (94.71 to 312.53) | 31.26 (14.94 to 49.39) | 1.23 (1.17 to 1.29) |
| Incidence | Qatar | 17.20 (6.67 to 31.25) | 13.86 (5.43 to 25.10) | 134.05 (53.09 to 241.06) | 18.55 (7.43 to 33.06) | 0.95 (0.89 to 1.00) |
| Incidence | Republic of Korea | 1,465.77 (455.49 to 2,723.12) | 10.83 (3.36 to 20.13) | 727.39 (199.77 to 1,365.79) | 7.89 (2.20 to 14.85) | -1.03 (-1.22 to -0.84) |
| Incidence | Republic of Moldova | 154.48 (66.00 to 269.82) | 15.24 (6.51 to 26.61) | 178.41 (94.04 to 279.61) | 29.36 (15.60 to 45.88) | 2.15 (2.09 to 2.21) |
| Incidence | Romania | 875.05 (347.09 to 1,535.38) | 17.09 (6.78 to 30.06) | 664.07 (295.60 to 1,092.40) | 22.42 (9.98 to 36.89) | 0.90 (0.84 to 0.97) |
| Incidence | Russian Federation | 10,045.02 (4,725.36 to 16,607.97) | 30.08 (14.09 to 49.62) | 8,547.60 (4,302.10 to 13,722.48) | 37.62 (18.93 to 60.31) | 0.72 (0.70 to 0.75) |
| Incidence | Rwanda | 299.03 (130.24 to 504.42) | 15.60 (6.65 to 26.47) | 471.32 (185.47 to 820.15) | 12.17 (4.67 to 21.39) | -0.83 (-0.95 to -0.70) |
| Incidence | Saint Kitts and Nevis | 3.59 (1.75 to 5.62) | 31.56 (15.33 to 49.41) | 5.47 (2.97 to 8.20) | 41.17 (22.37 to 61.53) | 0.85 (0.82 to 0.88) |
| Incidence | Saint Lucia | 11.19 (5.24 to 18.07) | 28.44 (13.37 to 45.94) | 18.39 (10.44 to 27.25) | 45.97 (26.15 to 68.12) | 1.58 (1.52 to 1.64) |
| Incidence | Saint Vincent and the Grenadines | 9.04 (4.63 to 14.44) | 28.19 (14.48 to 45.03) | 11.67 (6.21 to 17.43) | 44.96 (23.86 to 67.12) | 1.54 (1.47 to 1.60) |
| Incidence | Samoa | 18.58 (10.16 to 27.85) | 38.87 (21.23 to 58.30) | 30.27 (17.97 to 43.71) | 56.31 (33.26 to 81.43) | 1.23 (1.17 to 1.29) |
| Incidence | San Marino | 0.33 (0.08 to 0.72) | 5.52 (1.35 to 11.82) | 0.33 (0.07 to 0.71) | 6.08 (1.31 to 12.99) | 0.30 (0.22 to 0.38) |
| Incidence | Sao Tome and Principe | 11.34 (5.79 to 17.97) | 35.28 (17.78 to 56.21) | 32.61 (19.42 to 47.89) | 52.71 (30.98 to 77.90) | 1.30 (1.27 to 1.33) |
| Incidence | Saudi Arabia | 893.14 (391.49 to 1,519.57) | 19.91 (8.73 to 33.95) | 3,935.13 (2,041.49 to 6,156.73) | 41.06 (21.46 to 63.72) | 2.34 (2.25 to 2.43) |
| Incidence | Senegal | 530.59 (269.42 to 817.58) | 26.48 (13.22 to 41.13) | 1,338.61 (732.08 to 2,063.72) | 29.34 (15.86 to 45.52) | 0.31 (0.22 to 0.41) |
| Incidence | Serbia | 274.63 (93.43 to 498.96) | 12.73 (4.32 to 23.13) | 326.77 (135.81 to 543.01) | 19.22 (7.97 to 31.96) | 1.35 (1.30 to 1.39) |
| Incidence | Seychelles | 4.76 (2.07 to 7.89) | 22.34 (9.74 to 37.06) | 6.96 (3.58 to 11.17) | 31.37 (16.17 to 50.21) | 1.14 (1.11 to 1.17) |
| Incidence | Sierra Leone | 249.64 (118.39 to 399.77) | 22.84 (10.80 to 36.66) | 785.43 (439.77 to 1,202.21) | 29.95 (16.64 to 46.07) | 0.87 (0.82 to 0.92) |
| Incidence | Singapore | 124.87 (41.77 to 231.33) | 13.27 (4.39 to 24.57) | 95.82 (30.17 to 179.18) | 10.41 (3.13 to 19.60) | -0.72 (-0.84 to -0.60) |
| Incidence | Slovakia | 157.33 (51.19 to 298.81) | 13.14 (4.27 to 24.94) | 160.72 (65.59 to 278.19) | 17.63 (7.15 to 30.49) | 0.95 (0.88 to 1.02) |
| Incidence | Slovenia | 51.55 (15.17 to 96.72) | 11.41 (3.34 to 21.45) | 53.60 (22.03 to 91.23) | 17.95 (7.35 to 30.58) | 1.47 (1.42 to 1.51) |
| Incidence | Solomon Islands | 92.86 (60.27 to 128.83) | 100.75 (64.93 to 140.24) | 154.96 (96.75 to 213.16) | 84.72 (52.68 to 116.83) | -0.56 (-0.62 to -0.50) |
| Incidence | Somalia | 316.40 (145.07 to 521.07) | 15.89 (6.99 to 26.43) | 1,128.04 (576.79 to 1,742.07) | 18.24 (8.97 to 28.51) | 0.41 (0.34 to 0.49) |
| Incidence | South Africa | 3,082.87 (1,602.79 to 4,820.51) | 28.91 (15.09 to 45.17) | 3,970.57 (2,088.16 to 6,114.02) | 27.25 (14.28 to 42.01) | -0.22 (-0.31 to -0.13) |
| Incidence | South Sudan | 193.08 (77.44 to 330.98) | 11.34 (4.46 to 19.59) | 372.21 (177.49 to 606.18) | 13.84 (6.14 to 23.22) | 0.64 (0.55 to 0.73) |
| Incidence | Spain | 605.50 (138.40 to 1,272.00) | 6.31 (1.44 to 13.26) | 296.02 (71.36 to 629.37) | 4.28 (1.03 to 9.12) | -1.25 (-1.37 to -1.13) |
| Incidence | Sri Lanka | 998.44 (438.64 to 1,659.32) | 20.69 (9.09 to 34.39) | 1,300.97 (629.68 to 2,121.72) | 26.02 (12.59 to 42.46) | 0.79 (0.70 to 0.87) |
| Incidence | Sudan | 863.47 (347.13 to 1,478.42) | 16.22 (6.51 to 27.87) | 2,717.33 (1,218.90 to 4,449.70) | 21.60 (9.66 to 35.42) | 0.95 (0.84 to 1.05) |
| Incidence | Suriname | 31.48 (14.04 to 51.60) | 27.44 (12.22 to 45.00) | 55.21 (30.28 to 84.72) | 41.52 (22.74 to 63.75) | 1.35 (1.27 to 1.43) |
| Incidence | Sweden | 95.89 (25.53 to 204.75) | 5.33 (1.42 to 11.41) | 89.42 (23.86 to 187.01) | 4.72 (1.25 to 9.94) | -0.38 (-0.43 to -0.32) |
| Incidence | Switzerland | 113.79 (33.62 to 224.67) | 7.07 (2.03 to 14.04) | 107.12 (29.03 to 225.31) | 7.02 (1.84 to 14.77) | 0.00 (-0.04 to 0.03) |
| Incidence | Syrian Arab Republic | 849.50 (358.71 to 1,438.89) | 24.33 (10.20 to 41.25) | 996.84 (469.09 to 1,611.68) | 28.22 (13.08 to 46.39) | 0.49 (0.42 to 0.56) |
| Incidence | Taiwan (Province of China) | 830.49 (321.59 to 1,429.60) | 14.48 (5.55 to 24.91) | 775.30 (355.08 to 1,269.37) | 18.24 (8.28 to 29.79) | 0.75 (0.72 to 0.79) |
| Incidence | Tajikistan | 463.41 (241.48 to 733.56) | 30.75 (15.93 to 48.75) | 1,018.73 (584.22 to 1,537.88) | 39.03 (22.49 to 58.79) | 0.80 (0.75 to 0.85) |
| Incidence | Thailand | 5,742.08 (2,887.01 to 9,108.53) | 33.41 (16.77 to 53.08) | 3,978.43 (2,060.54 to 6,188.44) | 32.07 (16.71 to 49.81) | -0.10 (-0.18 to -0.03) |
| Incidence | Timor-Leste | 43.92 (19.87 to 70.35) | 20.31 (9.19 to 32.55) | 105.29 (52.18 to 163.23) | 25.08 (12.29 to 39.14) | 0.68 (0.65 to 0.70) |
| Incidence | Togo | 257.85 (130.61 to 400.29) | 25.92 (12.98 to 40.46) | 754.30 (425.47 to 1,122.12) | 33.01 (18.44 to 49.32) | 0.78 (0.75 to 0.82) |
| Incidence | Tokelau | 0.13 (0.07 to 0.19) | 32.82 (17.79 to 49.88) | 0.17 (0.10 to 0.25) | 52.94 (31.41 to 78.33) | 1.57 (1.51 to 1.64) |
| Incidence | Tonga | 8.73 (4.57 to 13.50) | 33.39 (17.53 to 51.79) | 12.47 (6.99 to 18.83) | 47.58 (26.54 to 71.84) | 1.16 (1.11 to 1.21) |
| Incidence | Trinidad and Tobago | 81.19 (36.33 to 133.45) | 24.68 (11.04 to 40.54) | 105.19 (58.39 to 159.88) | 38.82 (21.60 to 58.98) | 1.49 (1.44 to 1.53) |
| Incidence | Tunisia | 329.23 (128.08 to 592.05) | 13.90 (5.40 to 25.01) | 519.46 (223.96 to 908.23) | 21.04 (9.08 to 36.75) | 1.35 (1.25 to 1.45) |
| Incidence | Turkey | 2,344.82 (918.69 to 4,122.92) | 14.55 (5.71 to 25.59) | 3,978.52 (1,662.87 to 6,868.36) | 20.66 (8.66 to 35.62) | 1.11 (1.03 to 1.18) |
| Incidence | Turkmenistan | 533.19 (307.48 to 787.69) | 50.24 (28.91 to 74.33) | 1,030.55 (717.39 to 1,416.35) | 80.01 (55.94 to 109.66) | 1.52 (1.43 to 1.62) |
| Incidence | Tuvalu | 0.81 (0.44 to 1.26) | 35.22 (18.87 to 54.34) | 1.65 (0.98 to 2.44) | 50.46 (29.91 to 74.74) | 1.18 (1.14 to 1.22) |
| Incidence | Uganda | 427.40 (153.29 to 751.87) | 8.91 (3.12 to 15.82) | 1,382.13 (588.81 to 2,283.12) | 10.70 (4.44 to 17.91) | 0.58 (0.45 to 0.70) |
| Incidence | Ukraine | 1,732.42 (609.08 to 3,135.98) | 15.56 (5.45 to 28.18) | 2,110.59 (1,034.69 to 3,384.27) | 31.03 (15.31 to 49.58) | 2.27 (2.22 to 2.31) |
| Incidence | United Arab Emirates | 93.64 (35.52 to 163.44) | 18.39 (6.99 to 31.92) | 304.04 (144.00 to 501.35) | 30.73 (14.52 to 50.80) | 1.68 (1.59 to 1.77) |
| Incidence | United Kingdom | 887.76 (249.85 to 1,844.76) | 6.69 (1.87 to 13.89) | 899.87 (292.19 to 1,788.73) | 7.11 (2.28 to 14.12) | 0.22 (0.14 to 0.30) |
| Incidence | United Republic of Tanzania | 744.62 (281.63 to 1,313.81) | 10.50 (3.89 to 18.71) | 1,763.30 (655.36 to 3,081.24) | 10.66 (3.92 to 18.78) | 0.03 (-0.16 to 0.23) |
| Incidence | United States Virgin Islands | 6,478.58 (2,250.76 to 12,789.85) | 10.74 (3.69 to 21.17) | 5,990.84 (2,213.89 to 11,708.15) | 8.95 (3.28 to 17.48) | 1.47 (1.38 to 1.55) |
| Incidence | United States of America | 6.60 (3.03 to 10.66) | 26.98 (12.40 to 43.70) | 5.69 (3.10 to 8.68) | 42.27 (23.06 to 64.46) | -0.57 (-0.62 to -0.52) |
| Incidence | Uruguay | 42.67 (10.20 to 88.45) | 5.95 (1.42 to 12.33) | 56.16 (14.76 to 111.31) | 7.58 (1.98 to 15.02) | 0.80 (0.73 to 0.87) |
| Incidence | Uzbekistan | 3,582.72 (2,144.65 to 5,246.76) | 60.66 (36.22 to 88.92) | 6,256.32 (3,964.56 to 8,816.79) | 79.52 (50.94 to 110.87) | 0.89 (0.84 to 0.94) |
| Incidence | Vanuatu | 14.11 (7.79 to 21.15) | 35.44 (19.57 to 53.13) | 40.06 (24.49 to 58.28) | 47.74 (29.14 to 69.46) | 0.97 (0.89 to 1.05) |
| Incidence | Venezuela (Bolivarian Republic of) | 1,325.63 (623.30 to 2,187.28) | 24.73 (11.59 to 40.84) | 1,840.09 (992.68 to 2,907.91) | 34.41 (18.55 to 54.46) | 1.08 (1.02 to 1.15) |
| Incidence | Viet Nam | 3,454.62 (1,502.84 to 5,659.49) | 17.67 (7.67 to 28.97) | 5,042.21 (2,547.38 to 8,186.49) | 23.74 (12.14 to 38.21) | 0.98 (0.94 to 1.02) |
| Incidence | Yemen | 466.69 (187.91 to 811.68) | 15.00 (6.03 to 26.12) | 1,631.34 (698.08 to 2,770.34) | 18.16 (7.72 to 30.90) | 0.60 (0.53 to 0.67) |
| Incidence | Zambia | 395.96 (178.01 to 650.12) | 17.06 (7.46 to 28.39) | 1,099.27 (561.30 to 1,732.47) | 19.02 (9.52 to 30.25) | 0.33 (0.24 to 0.41) |
| Incidence | Zimbabwe | 557.47 (243.84 to 908.03) | 20.43 (9.05 to 33.11) | 1,081.32 (529.85 to 1,695.44) | 25.76 (12.71 to 40.27) | 0.73 (0.66 to 0.81) |
